# Alkane Dehydrogenation and H/D Exchange by a Cationic Pincer-Ir(III) Hydride: Cooperative C–H Addition and β‑H Elimination Modes Induce Anomalous Selectivity

**DOI:** 10.1021/jacs.4c16699

**Published:** 2025-03-13

**Authors:** Ashish Parihar, Thomas J. Emge, Faraj Hasanayn, Alan S. Goldman

**Affiliations:** † Department of Chemistry and Chemical Biology, 242612 Rutgers, The State University of New Jersey, New Brunswick, New Jersey 08903, United States; ‡ Department of Chemistry, 11238 American University of Beirut, Beirut 1107 2020, Lebanon

## Abstract

We report that the cationic iridium complex (^iPr^PCP)­IrH^+^ catalyzes the transfer-dehydrogenation of alkanes to give alkenes and hydrogen isotope exchange (HIE) of alkanes and arenes. Contrary to established selectivity trends found for C–H activation by transition metal complexes, strained cycloalkanes, including cyclopentane, cycloheptane, and cyclooctane, undergo C–H addition much more readily than *n*-alkanes, which in turn are much more reactive than cyclohexane. Aromatic C–H bonds also undergo H/D exchange much less rapidly than those of the strained cycloalkanes, but much more favorably than cyclohexane. The order of reactivity toward dehydrogenation correlates qualitatively with the reaction thermodynamics, but the magnitude is much greater than can be explained by thermodynamics. Accordingly, the cycloalkenes corresponding to the strained cycloalkanes undergo hydrogenation much more readily than cyclohexene, despite the less favorable thermodynamics of such hydrogenations. Computational (DFT) studies allow rationalization of the origin of reactivity and the unusual selectivity. Specifically, the initial C–H addition is strongly assisted by β-agostic interactions, which are particularly favorable for the strained cycloalkanes. Subsequent to α-C–H addition, the H atom of the β-agostic C–H bond is transferred directly to the hydride ligand of (^iPr^PCP)­IrH^+^ to give a dihydrogen ligand. The overall processes, C–H addition and β-H-transfer to hydride, are calculated to generally have minima on the IRC surface although not necessarily on the enthalpy or free energy surfaces; these minima are extremely shallow such that the 1,2-dehydrogenations are effectively concerted although asynchronous.

## Graphical abstract

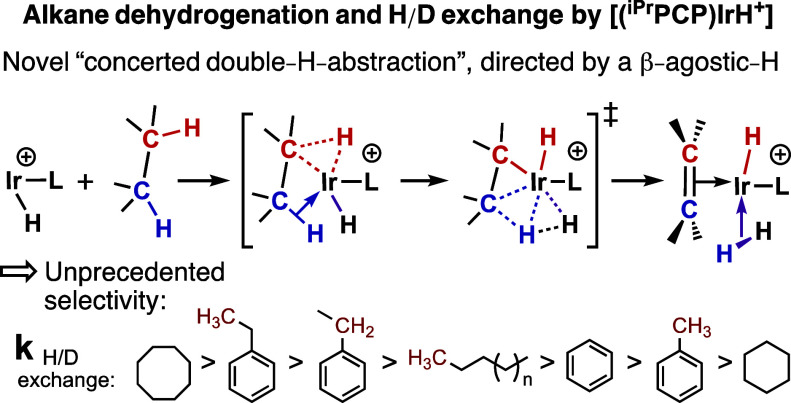

## Introduction

Iridium has played a prominent role in the chemistry of C–H activation and functionalization, largely through C–H activation by low oxidation state (specifically, Ir^I^) centers,^1–5^ although examples of C–H activation by high-valent complexes have long been known as well.^6–11^ With respect to catalysis, alkane dehydrogenation by pincer-ligated low-valent iridium complexes has been particularly well studied.^12,13^ In the past decade, however, there has been significant progress in the development of pincer-iridium-based catalysts believed to operate entirely via high-valent oxidation states.^14–18^ Recently, we reported that the (*p*-pyridyl-^tBu^PCP)­IrCl^+^ cation (*p*-pyridyl-^tBu^PCP = 3,5-bis­(di-*tert*-butylphosphinomethyl)-2,6-dimethylpyridin-4-yl) undergoes facile intramolecular C­(sp^3^)–H activation (cyclometalation).^19^ We computationally investigated C–H activation by the parent Ir^III^ complex (^tBu^PCP)­IrCl^+^ (^R^PCP = 2,6-C_6_H_3_(CH_2_PR_2_)) and the less crowded analog (^iPr^PCP)­IrCl^+^. DFT calculations indicated that (^iPr^PCP)­IrCl^+^ would more favorably undergo intermolecular rather than intramolecular C–H activation (Scheme 1). Moreover, intermolecular C–H addition of alkanes by this fragment was predicted to lead to dehydrogenation to yield olefins.^19^

**Scheme 1:**
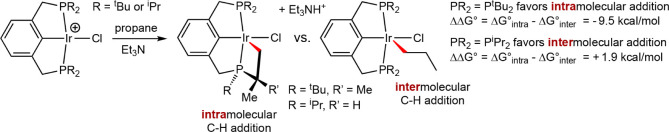
Relative Free Energies for Cyclometalation (Intramolecular C­(sp^3^)–H Activation) versus Intermolecular C­(sp^3^)–H Activation (Propane Used as Model Alkane; from ref 19)

High-valent systems for dehydrogenation of alkanes or alkyl groups hold the intriguing possibility that they might tolerate functional groups or reagents not compatible with low-valent catalysts, or that they might offer complementary selectivity. Electron-poor catalysts could potentially function under, or be generated by, oxidizing conditions incompatible with low-valent catalysts, perhaps in electrochemical systems.^20^ In consideration of these points, we attempted to pursue an experimental investigation of the predicted catalytically active fragment (^iPr^PCP)­IrCl^+^. While we were unsuccessful in this effort, we found that the isoelectronic fragment (^iPr^PCP)­IrH^+^ is an active catalyst for alkane dehydrogenation and H/D exchange. Surprisingly, it shows selectivity that is dramatically different from that of low-valent species (most notably, (^iPr^PCP)Ir and related fragments). Calculations indicate this selectivity can be rationalized based on a novel pathway, one that is very distinct from pathways that have previously^12,13,21^ been reported for (^R^PCP)­Ir-based and other low-valent transition-metal-based catalysts.^22^

## Experimental Results and Discussion

### Synthesis of (^iPr^PCP)­IrH^+^

Motivated by the experimental and computational studies noted above, we attempted to synthesize (^iPr^PCP)­IrCl^+^ via the reaction of (^iPr^PCP)­IrHCl (**1-HCl**) with [H­(Et_2_O)_2_]­[BArF^24^][Bibr ref23] (Scheme 2), as well as with H­[PF_6_] and HCl.^24^ These efforts, however, were unsuccessful.

**Scheme 2:**
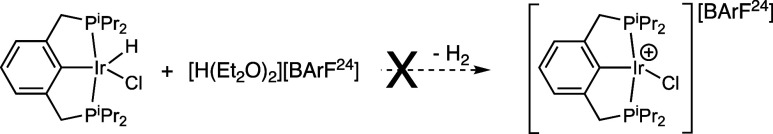
Attempted Generation of (^iPr^PCP)­IrCl^+^ by Reaction of **1-HCl** with [H­(Et_2_O)_2_]­[BArF^24^]

We therefore considered the investigation of the isoelectronic fragment (^iPr^PCP)­IrH^+^ (**1-H** ^**+**^), an analog of (^tBu^POCOP)­IrH^+^ (^tBu^POCOP = 2,6-C_6_H_3_(OP^t^Bu_2_)_2_), the chemistry of which has been extensively developed by Brookhart and coworkers.^25–31^ Toward this end, **1-HCl** was treated with M­[BArF^20^] or M­[BArF^24^] (M = Na, Li, K; BArF^20^ = B­(C_6_F_5_)_4_; BArF^24^ = B­[3,5-C_6_H_3_(CF_3_)_2_)]_4_; Scheme 3). The room-temperature ^31^P­{^1^H} NMR spectrum of a light orange toluene-*d*_8_ solution resulting from the reaction with Na­[BArF^24^] predominantly featured a very broad peak at δ 55 with several minor sharp peaks at δ 36 to δ 55. At 100 °C, however, the ^31^P­{^1^H} NMR spectrum showed only a single fairly sharp peak at δ 59.7. The ^1^H NMR spectrum at 100 °C showed only one, fairly broad, peak upfield of δ 0 (i.e., the hydride region), specifically at δ −41.59, integrating as 1 H relative to the characteristic pincer-ligand and BArF^24^ signals, indicative of a hydride ligand positioned trans to a vacant coordination site.^32^ When the temperature was lowered, this peak broadened further, and at room temperature, it was not observable, nor were any other signals seen in the upfield region. We attribute these observations to the presence of only one species in solution at 100 °C, best described as (^iPr^PCP)­IrH^+^ (**1-H** ^**+**^), although weak and dynamic interactions with solvent, anion, or other species in solution seem likely. At room temperature, such interactions, perhaps including the binding of solvent impurities, are presumably less dynamic.

**Scheme 3:**
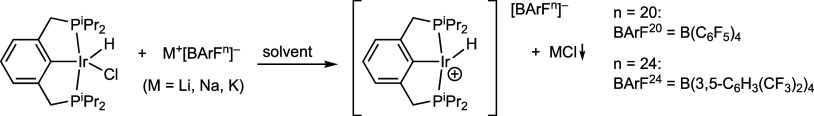
Generation of [**1-H** ^**+**^]­[BArF^n^] by the Reaction of **1-HCl** with M­[BArF^n^]

### (^iPr^PCP)­IrH^+^: Addition of Small Molecules and Further Characterization

When a CO atmosphere was added to a dichloromethane solution of [**1-H** ^**+**^]­[BArF^24^] followed by the removal of solvent and dissolution in CDCl_3_, the ^31^P­{^1^H} NMR spectrum showed a single sharp signal at δ 51.11, while a signal at δ −10.42 (t, ^3^ *J*_PH_ = 12.8 Hz) was found in the ^1^H NMR spectrum, suggestive of the formation of [**1-H­(CO)**_**2**_^**+**^]­[BArF^24^] (Scheme 4). Crystals were obtained by vapor diffusion of pentane into a benzene solution, and characterization as [*cis-* **1-H­(CO)**_**2**_^**+**^]­[BArF^24^] was confirmed by single-crystal X-ray diffraction (SCXRD) (Figure 1).

**Scheme 4:**
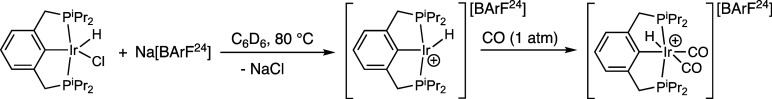
Reaction of **1-HCl** with Na­[BArF^24^] to Generate Putative **1-H** ^**+**^ under a CO Atmosphere Leading to **1-H­(CO)**_**2**_^**+**^

**Figure 1:**
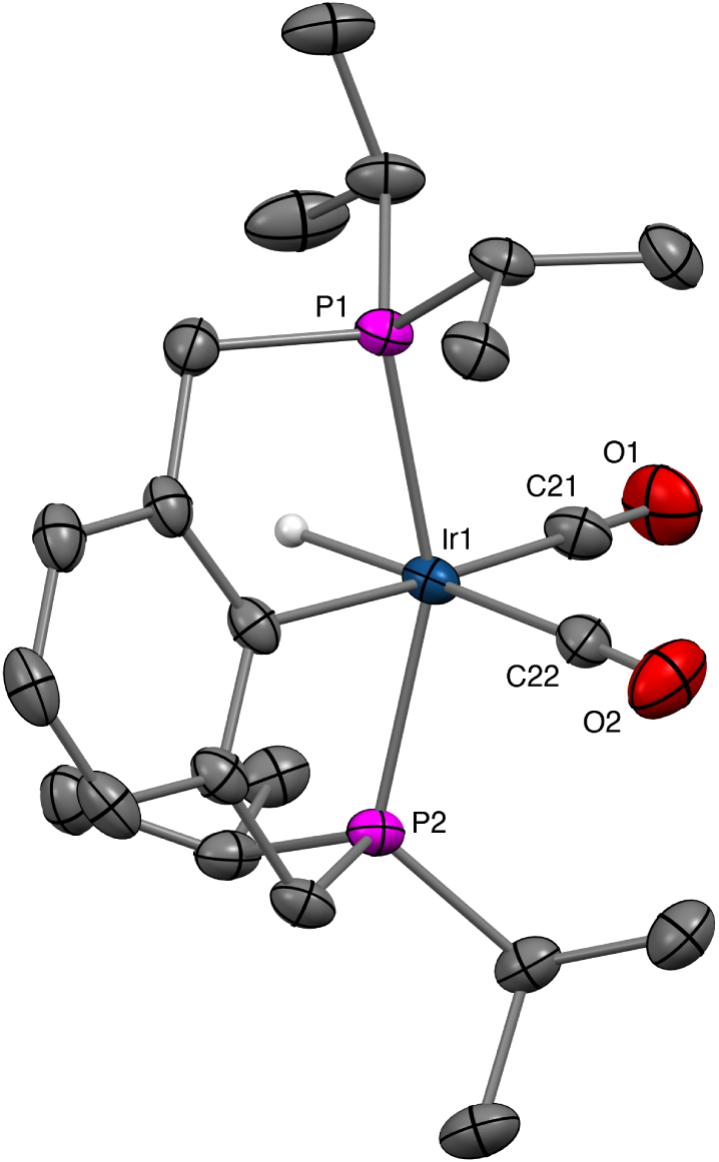
Molecular structure of the [*cis-*(^iPr^PCP)­IrH­(CO)_2_] cation determined by SCXRD. Hydrogen atoms, except for the hydride ligand, and BArF^24^ anion omitted for clarity.

Addition of the H_2_ atmosphere to a toluene-d_8_ solution of **1-H** ^**+**^ resulted in the formation of two signals in the ^31^P­{^1^H} NMR spectrum at approximately δ 55 and δ 57 ppm in a ratio of ca. 70:30. In the ^1^H NMR spectrum (toluene-d_8_), two peaks in the metal-hydride region were observed at approximately δ −3.99 ppm and δ −26.88, in a ratio of 2:1, consistent with an assignment of [(^iPr^PCP)­IrH­(H_2_)]­[BArF^24^] ([**1-H­(H**_**2**_ **)**]­[BArF^24^]); the ^tBu^POCOP analog of this species has been reported by Brookhart and coworkers.^30^ HMBC spectroscopy revealed that the hydride signals correlated with the major signal in the ^31^P­{^1^H} NMR spectrum.

Propene, *t-*butylethylene (TBE), and cyclopentene (CPE) were added to separate toluene-d_8_ solutions of [**1-H** ^**+**^]­[BArF^24^]. In all three cases, an upfield signal was observed in the ^1^H NMR spectrum (−34.2 ppm, −30.7 ppm, and −33.4 ppm, respectively), indicative of an olefin complex with a hydride ligand positioned trans to a vacant coordination site, and therefore presumably 1-H­(alkene)^+^. In the cases of TBE and CPE, the ^1^H NMR signals due to the free olefin in solution were broad, indicating exchange between free and coordinated olefin (signals attributable to coordinated olefin were not observed). With propene, the free olefin signal was sharp at room temperature but broadened at higher temperature, ca. 320 K. A single peak was observed in the ^31^P­{^1^H} NMR spectrum with each alkene: propene, 46.3 ppm; TBE, 51.2 ppm (broad); and CPE, 38.8 ppm (details are found in the Supporting Information).

When 1 atm ethylene was added to a toluene-d_8_ solution of [**1-H** ^**+**^]­[BArF^24^] at room temperature, the ^1^H NMR signal attributable to free ethylene was very broad at 5.1 ppm (cf. 5.25 ppm in the absence of a metal complex).^33^ In the ^31^P­{^1^H} NMR spectrum, an extremely broad signal is observed at 29.6 ppm. No hydride signal was observed at room temperature, but at 233 K, a signal appears at −13.3 ppm, attributable, in contrast to the complexes discussed above, to a hydride positioned trans to a strong trans-influence ligand. At this temperature, the free ethylene signal in the ^1^H NMR spectrum was relatively sharp, at 5.24 ppm. In the ^31^P­{^1^H} NMR spectrum, an extremely broad signal is observed at 29.6 ppm at room temperature, while at 233 K, a relatively sharp signal is found at 27.18 ppm. Under 1.6 atm ethylene, the signal in the room-temperature ^31^P­{^1^H} NMR spectrum was sharper than observed under 1.0 atm and shifted to 28.6 ppm. These results are all consistent with rapid exchange between coordinated and free ethylene, and an equilibrium between the mono- and bis-ethylene complexes, **1-H­(C_2_H_4_)^+^** and **1-H­(C_2_H_4_)_2_^+^**. Thus, ethylene, propene, CPE, and TBE are all found to bind to **1-H** ^**+**^ and to undergo exchange between free and coordinated alkene on the NMR time scale. Only in the case of the least sterically demanding alkene, ethylene, is there evidence for a significant equilibrium concentration of bis-alkene complex, which is apparently the major species in solution at 233 K.

### Alkane Dehydrogenation by **1-H** ^**+**^: Initial Screenings

The addition of Li­[BArF^20^] to cyclooctane (COA) solutions containing TBE and **1-HCl** was found to result in high rates of catalytic COA-to-TBE transfer dehydrogenation (Figure 2a). No dehydrogenation was observed under the same conditions when no alkali metal salt was added to the **1-HCl**. Addition of 3 equiv Li­[BArF^20^] gave somewhat greater activity than with 1 equiv Li­[BArF^20^], but the use of 6 equiv Li­[BArF^20^] gave no more activity than with 3 equiv; this seems consistent with incomplete halide removal by 1 equiv Li­[BArF^20^], and essentially complete removal with the use of 3 equiv.

**Figure 2:**
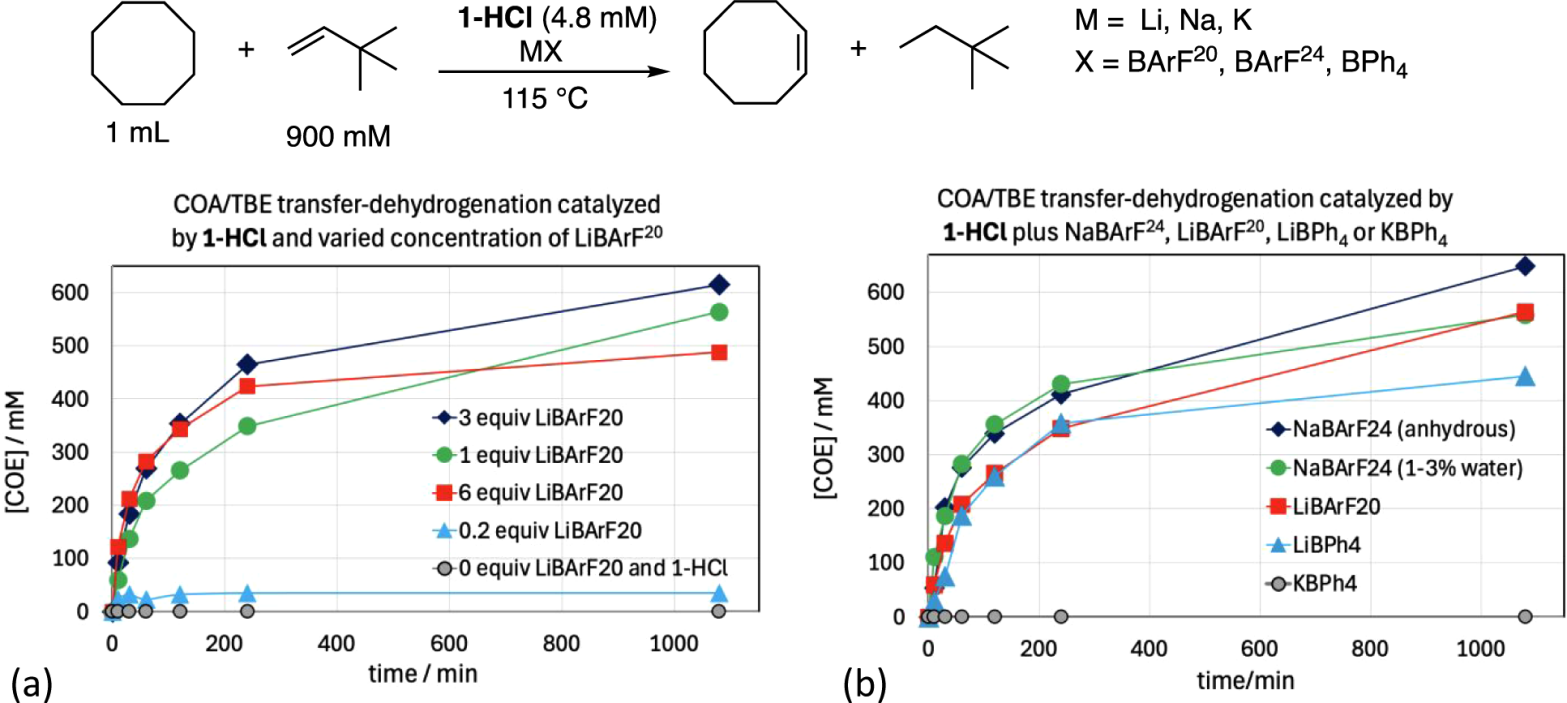
(a) COA/TBE transfer-dehydrogenation catalyzed by **1-HCl** and various quantities of Li­[BArF^20^]. (b) COA/TBE transfer-dehydrogenation catalyzed by **1-HCl** and Li­[BPh_4_], Na­[BArF^24^], or KBPh_4_.

Salts of Li^+^, Na^+^, and K^+^ were investigated, with noncoordinating anions including BPh_4_, BArF^20^, and BArF^24^ (Figure 2b). No dehydrogenation was obtained with K­[BPh_4_] presumably due to very poor solubility. 1.0 equiv Na­[BArF^24^] (either anhydrous or 1–3% H_2_O) afforded a level of catalytic activity very comparable to 3 equiv Li­[BArF^20^]. Additional Na­[BArF^24^] had no significant effect. These results suggest that chloride was fully removed with the use of 1.0 equiv Na­[BArF^24^]. Subsequently, for purposes described below, unless indicated otherwise, **1-H** ^**+**^ was prepared in situ by the reaction of **1-HCl** with Na­[BArF^24^] at 115 °C.^34^

Several hydrogen acceptors were investigated for COA transfer-dehydrogenation. 1-Hexene and *t*-butylpropene (TBPE; 4,4-dimethylpent-1-ene) were found to undergo rapid isomerization to form internal olefins, affording low rates and turnover numbers (TONs) for transfer-dehydrogenation^35,36^ (Figure 3a). TBE proved significantly more effective than other acceptors and was used as the standard acceptor for further transfer-dehydrogenation experiments in this study.^1^

**Figure 3:**
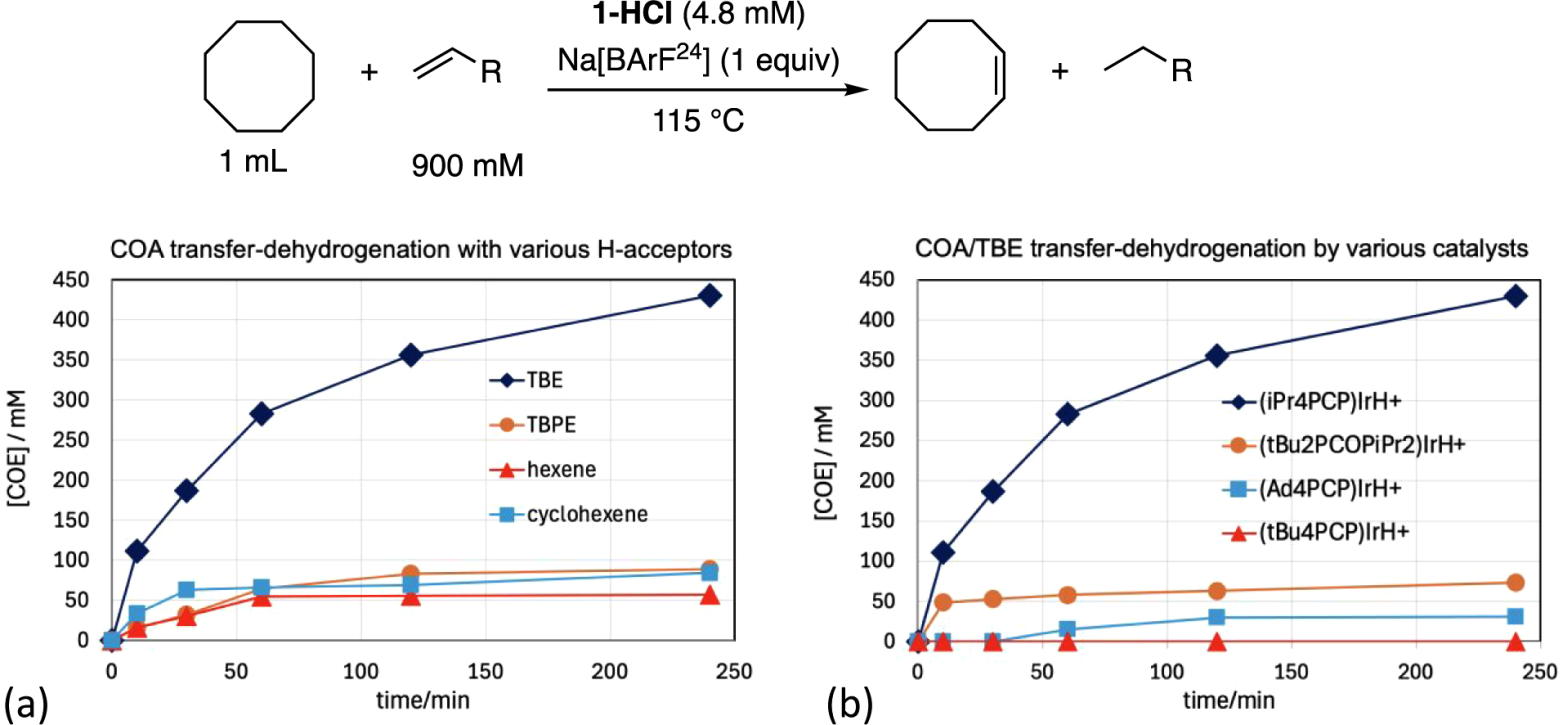
(a) COA transfer-dehydrogenation by **1-H** ^**+**^ with the use of various hydrogen acceptors. (b) COA/TBE transfer-dehydrogenation by various complexes (pincer)­IrHCl and MX (presumed to generate the corresponding species (pincer)­IrH^+^).

Iridium complexes of other PCP-type pincer ligands were investigated for catalytic activity under the same conditions as described above (Figure 3b). The archetypal (^tBu^PCP)­IrHCl gave no observable COA/TBE transfer-dehydrogenation activity; the contrast with **1-HCl** is particularly striking considering that the corresponding (^tBu^PCP)­Ir^I^ fragment is generally highly effective for transfer-dehydrogenation (albeit less so than (^iPr^PCP)­Ir^I^).^35^ This may be attributable to purely steric factors; we have calculated in the case of the (^R^PCP)­IrCl^+^ cation that intermolecular C–H bond addition (a terminal C–H bond of propane) has a barrier, Δ*G* ^‡^, 7.5 kcal/mol higher for ^tBu^PCP than for ^iPr^PCP.^19^ Alternatively, the negligible activity of (^tBu^PCP)­IrHCl may be attributable to cyclometalation; both kinetically (ΔΔ*G* ^‡^ = −2.0 kcal/mol) and thermodynamically (ΔΔ*G*° = −4.7 kcal/mol), (^tBu^PCP)­IrCl^+^ is calculated to be much more prone to cyclometalation than (^iPr^PCP)­IrCl^+^.^19^

The tetra-adamantyl substituted complex (^Ad^PCP)­IrHCl showed activity slightly higher than (^tBu^PCP)­IrHCl, but still much less than (^iPr^PCP)­IrHCl; the increase vis-à-vis (^tBu^PCP)­IrHCl may be due to ^Ad^PCP being slightly less sterically demanding or its greater resistance to cyclometalation (Figure 3b).^37^ The mixed di-*t*-butylphosphino/di-*i*-propylphosphino complex (^tBu2^PCOP^iPr2^)­IrHCl was found to be more active than (^Ad^PCP)­IrHCl or (^tBu^PCP)­IrHCl, but still much less so than (^iPr^PCP)­IrHCl. Accordingly, all further work in this study utilized only the ^iPr^PCP pincer ligand.

### Chemoselectivity of Dehydrogenation: Comparison Between (PCP)­Ir^I^ and (PCP)­Ir^III^H^+^

Precursors of (pincer)­Ir^I^, including (^iPr^PCP)­Ir^I^, are among the most effective and well-known catalysts for alkane dehydrogenation, typically operating via an Ir^I^/Ir^III^ cycle.^13,38,39^ Given that the present system shares the same (^iPr^PCP)­Ir unit, we felt it was critical to investigate the possibility that the two systems operated via the same catalytically active species (even though there were no obvious bases or reductants present in the above-described examples of catalysis achieved with Ir^III^ precursors). To this end, we conducted selectivity competition experiments with sources of (^iPr^PCP)­Ir^I^ and with the putative fragment (^iPr^PCP)­Ir^III^H^+^. These experiments not only demonstrated that the systems operated via different catalytically active species, but they also revealed remarkable and unexpected differences in selectivity.

Addition of KO^t^Bu to **1-HCl** is known to generate the active species (^iPr^PCP)­Ir^I^,^40,41^ while the addition of Li­[BArF^20^] is presumed, based on the experiments described above, to generate (^iPr^PCP)­IrH^+^ (Scheme 3). Experiments were conducted in which a competition for dehydrogenation of COA versus *n*-decane was established using TBE as the hydrogen acceptor, with **1-HCl** and 1 equiv of either KO^t^Bu or LiBArF^20^. In the case of KO^t^Bu addition, greater activity, by a factor of ca. 2 on a per mol basis, was observed for the dehydrogenation of *n-*decane versus COA (Figure 4a). In contrast, the use of LiBArF^20^ resulted in much greater (>200-fold) activity toward COA relative to *n*-decane (Figure 4b). These strongly contrasting selectivities support the conclusion of fundamental differences in the respective catalytic cycles of these two systems.

**Figure 4:**
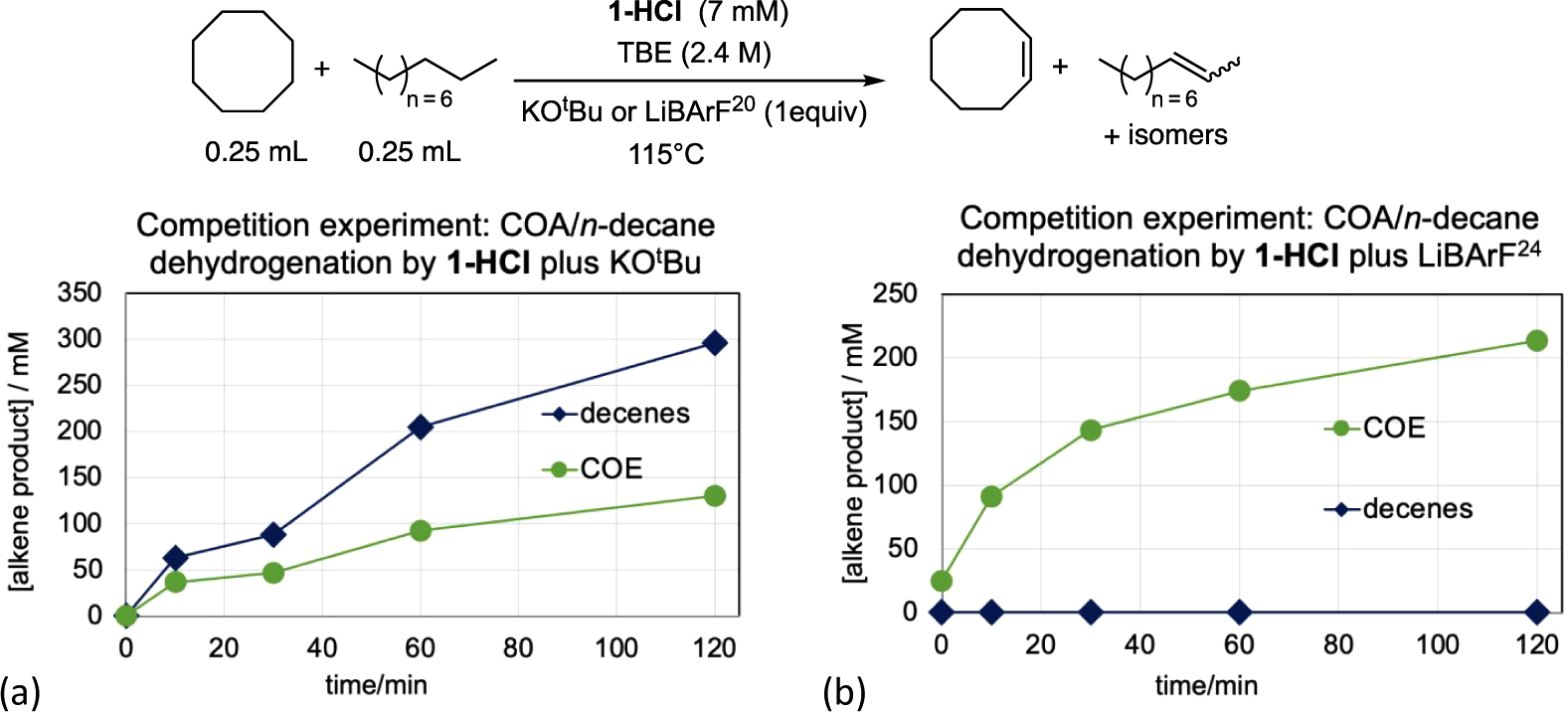
Competition experiments: transfer dehydrogenation of COA versus *n*-decane by (a) **1-HCl** and KO^t^Bu (presumably generating (^iPr^PCP)­Ir^I^) and (b) by **1-HCl** and Li­[BArF^20^] (presumably generating (PCP)­Ir^III^H^+^).

The same competition was conducted but catalyzed by (^iPr^PCP)­Ir­(ethylene) plus a proton source, [H­(Et_2_O)_2_]­[BArF^24^]. The results (Figure 5) were effectively the same as obtained with (^iPr^PCP)­IrHCl and Na­[BArF^24^], indicating a common, catalytically active fragment, presumably (^iPr^PCP)­IrH^+^.

**Figure 5:**
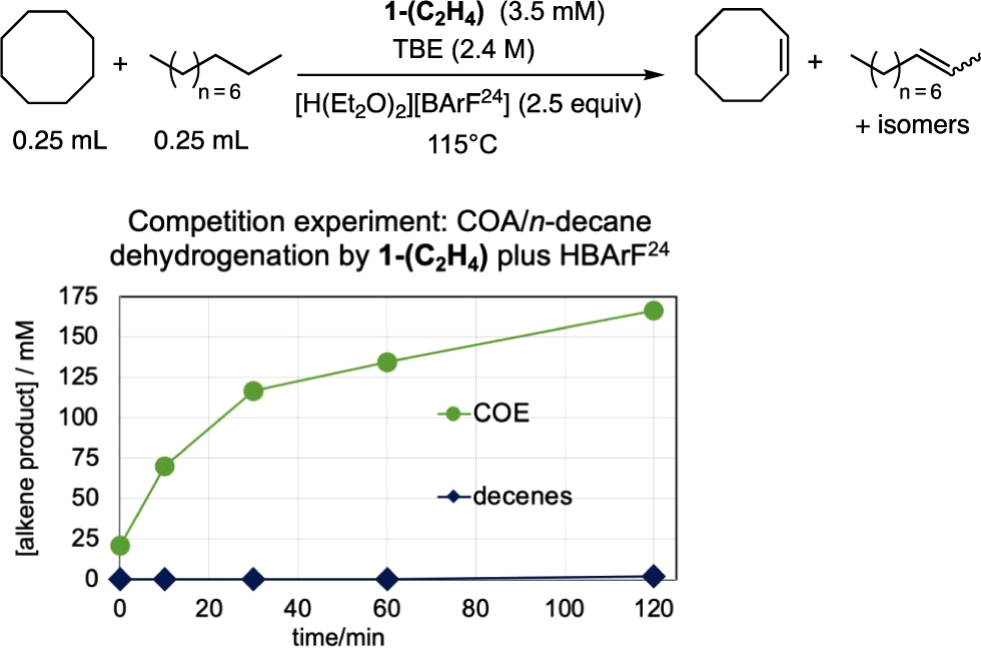
Competition experiment: transfer-dehydrogenation of COA versus *n*-decane catalyzed by (^iPr^PCP)­Ir­(ethylene) and [H­(Et_2_O)_2_]­[BArF^24^] (cf. Figure 4b).

### Further Transfer-Dehydrogenation Competition Experiments Using **1-H** ^**+**^

Intrigued by the remarkably high selectivity for dehydrogenation of COA versus an *n*-alkane, we investigated the competition of other cycloalkanes with *n*-decane. We note that the results of competition experiments are determined solely by energy differences between the rate-determining TSs for different substrates.^42^ In contrast, rates obtained from individual experiments are also controlled by relative resting state energies, which likely vary depending upon the binding energies of the respective alkene product, and may be further complicated by many factors such as allyl or diene formation, as well as any impurities present in the substrate sources.

**1-H** ^**+**^ (generated by the reaction of **1-HCl** with Na­[BArF^24^]) showed high selectivity for the dehydrogenation of cyclopentane (CPA) versus *n*-decane, as was observed for COA versus *n*-decane, although “only” by a factor of ca. 13 (Figure 6a). Cyclododecane (CDA), however, was found to be much *less* reactive than *n*-decane (by a factor of ca. 20; Figure 6b). Thus, the relative activity of the cycloalkanes (values relative to *n*-decane) is as follows: COA (>100:1) > CPA (ca. 10:1) > CDA (ca. 0.05:1). This order among the cycloalkanes may be noted to correlate with their ring strain (Table 1): COA (9.6 kcal/mol) > CPA (6.5 kcal/mol) > CDA (4.8 kcal/mol).

**Figure 6:**
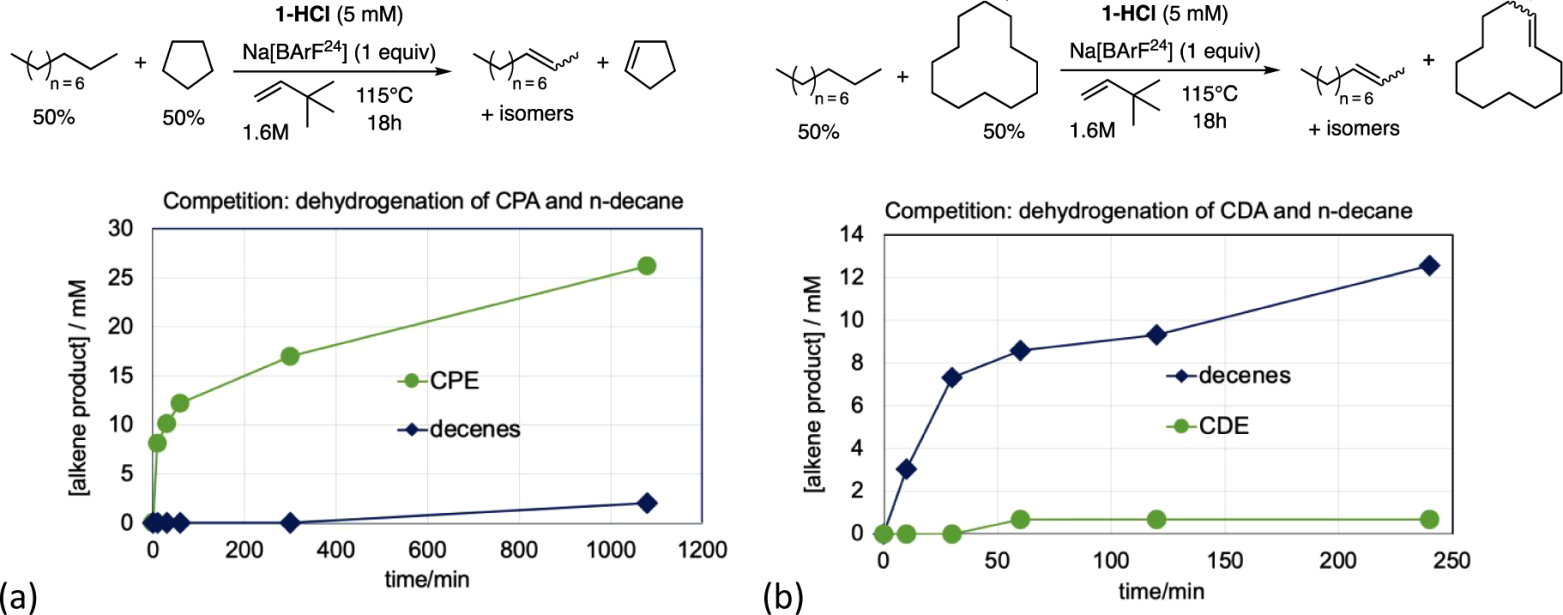
Transfer dehydrogenation catalyzed by **1-H** ^**+**^ with TBE as the hydrogen acceptor. Competition experiment of (a) cyclopentane (CPA) versus *n*-decane and (b) cyclododecane (CDA) versus *n*-decane.

**Table 1:** Ring strain and dehydrogenation enthalpies of relevant alkanes

Alkane substrate	Dehydrogenation product	Ring strain (cycloalkane)^a^	Ring strain/CH_2_ (cycloalkane)	Dehydrogenation enthalpy relative to C_6_H_12_ ^b^
cyclopentane (CPA)	cyclopentene (CPE)	6.2	1.2	–1.2
cyclohexane (CHxA)	cyclohexene (CHxE)	0.1	0.0	0.0
cycloheptane (CHpA)	cycloheptene (CHpE)	6.2	0.9	–2.0
cyclooctane (COA)	cyclooctene (COE)	9.7	1.2	–3.5
cyclododecene (CDA)	cyclododecene (CDE)	4.1	0.34	–1.7
*n*-decane	1-decene	--	--	+2.0
*n*-decane	trans-2-decene	--	--	–0.2
t-butylethane (TBA)	TBE	--	--	+2.1

a From ref 43.

b From ref 44. Negative values indicate that dehydrogenation of the specified alkane is less endothermic than dehydrogenation of cyclohexane.

Thus, CDA was found to be far less reactive than *n*-alkanes toward dehydrogenation by **1-H** ^**+**^ and *n*-alkanes in turn far less reactive than COA or CPA. Nevertheless, cyclohexane (CHxA) was found to be even much less reactive than CDA (Figure 7). Indeed, no cyclohexene was observed in a CDA/CHxA dehydrogenation competition experiment; thus, the ratio could not be quantified, but the extremely low level of susceptibility of CHxA toward dehydrogenation by **1-H** ^**+**^ can clearly be inferred.

**Figure 7:**
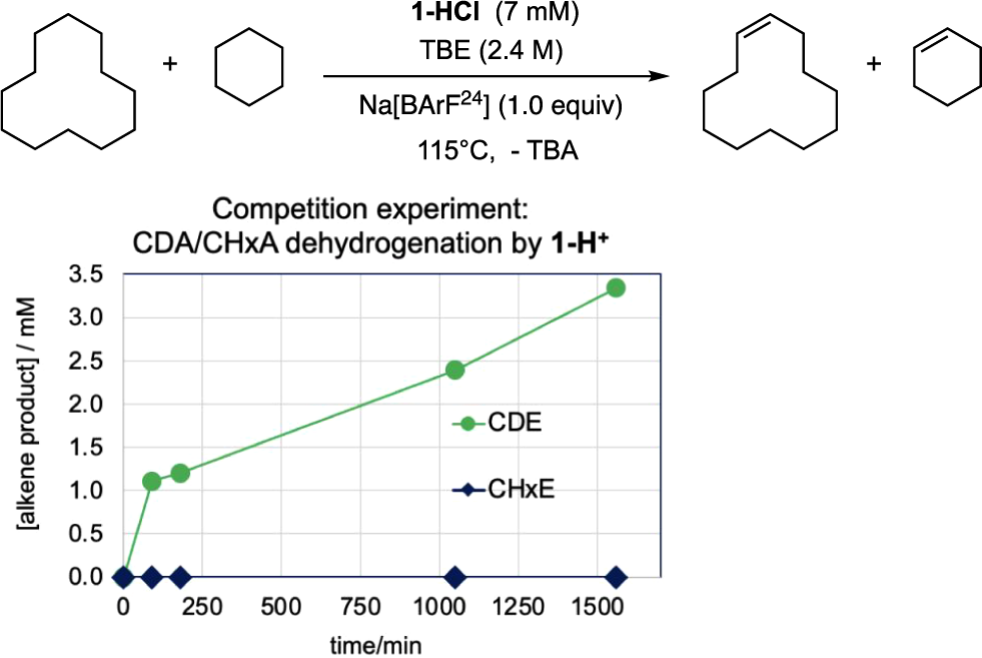
Transfer dehydrogenation catalyzed by **1-H** ^**+**^ with TBE as the hydrogen acceptor. Competition experiment of cyclododecane (CDA) versus cyclohexane (CHxA).

### Hydrogenation by (PCP)­IrH^+^

A correlation of dehydrogenation rates with ring strain among cycloalkanes is not in itself surprising. Ring strain in medium-sized rings generally correlates with the thermodynamics of dehydrogenation (Table 1 shows ring strain and dehydrogenation enthalpies), and the dehydrogenation thermodynamics should in turn correlate with rates if the rate-determining transition state has some cycloalkene character. However, we found the large magnitude of the dehydrogenation selectivity to be surprising.

While the very high reactivities of COA and CPA relative to *n*-decane are consistent with their lower enthalpy (lesser endothermicity) of dehydrogenation, the very low relative reactivity of CDA does not seem to correlate with its dehydrogenation enthalpy. (The dehydrogenation enthalpy of CDA is slightly less than that of CPA, for example, and significantly less than dehydrogenation of *n*-decane to give either terminal or internal olefins; Table 1). In order to further investigate the relative reactivity of these substrates, we studied the reverse reaction, hydrogenation of the corresponding olefins.

Hydrogenation (with H_2_) is not the microscopic reverse of transfer-dehydrogenation. Nevertheless, if the rate-determining step follows the addition of olefin and net addition of 2 H to the metal center in pathways for both transfer-hydrogenation and for hydrogenation using H_2_, the selectivity in competition experiments should be the same regardless of the hydrogen source. In accord with this point, the very high reactivity of (^iPr^PCP)­IrH^+^ for the *dehydrogenation* of COA versus linear alkanes was also reflected in *hydrogenation* competition experiments. Competition experiments between hydrogenation of COE versus TBE were performed using (^iPr^PCP)­Ir^I^ and using (^iPr^PCP)­Ir^III^H^+^. The rapid rate of the hydrogenation reaction, with either catalyst, precluded direct determination of the reaction kinetics. Instead, hydrogen gas (H_2_) was incrementally added to the reaction vessel and concentrations were determined by ^1^H NMR spectroscopy, allowing the determination of relative rates. When catalyzed by (PCP)­Ir^I^ (**1-HCl**/KO^t^Bu) hydrogenation was essentially completely selective for TBE, with negligible COE hydrogenation detected (Figure 8a), consistent with the much more favorable thermodynamics of TBE hydrogenation. In striking contrast, when catalyzed by **1-H** ^**+**^ (**1-HCl**/Na­[BArF^24^]), hydrogenation of COE and TBE proceeded at virtually identical rates (Figure 8b), highlighting the point that the Ir^I^ and Ir^III^ catalysts operate through very different mechanisms and, in particular, the far greater relative effectiveness of the IrH^+^ system for COA/COE dehydrogenation/hydrogenation.

**Figure 8:**
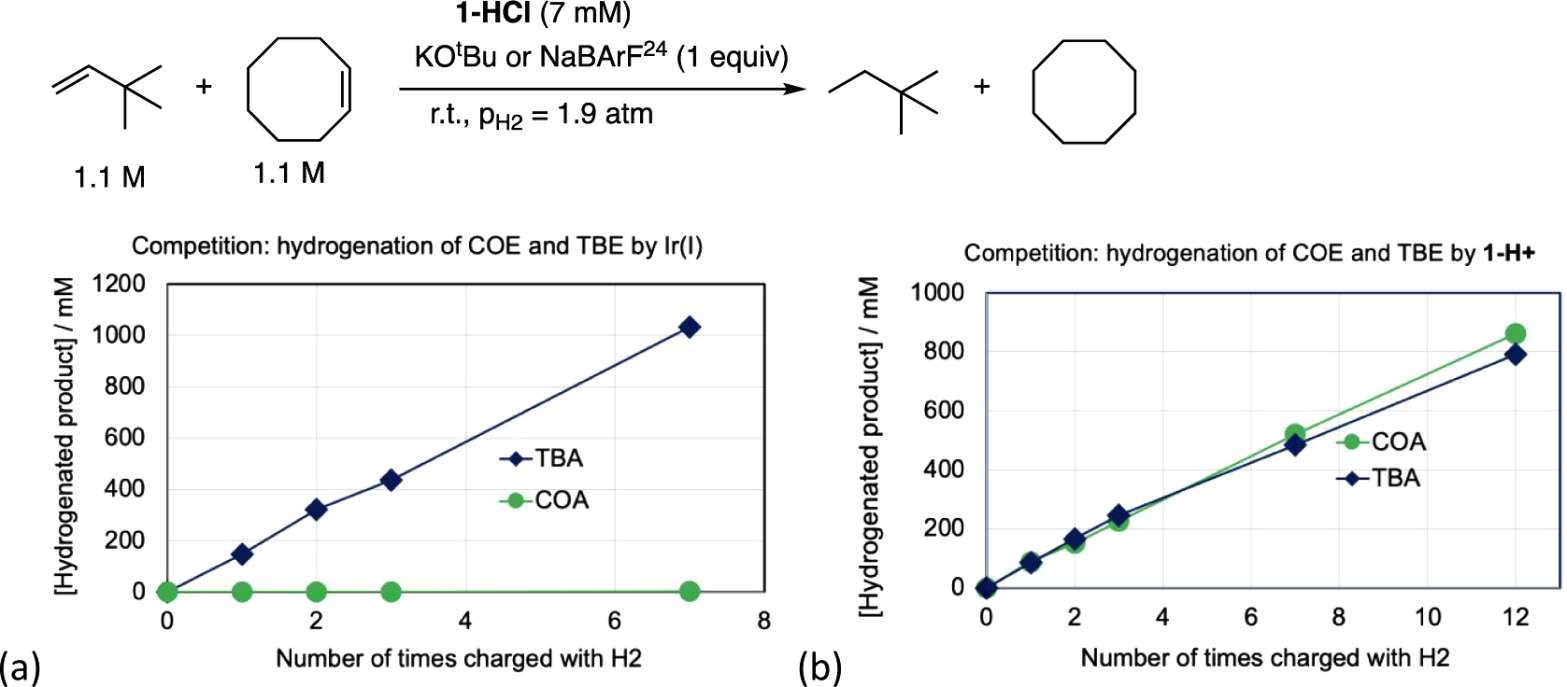
(a) Competition hydrogenation experiments: (a) COE versus TBE catalyzed by (^iPr^PCP)­Ir­(I) and (b) COE versus TBE hydrogenation catalyzed by (^iPr^PCP)­IrH^+^.

The reaction enthalpy of TBE hydrogenation is 5.6 kcal/mol more negative than that of COE hydrogenation (Table 1). Given that and considering that COE is a disubstituted olefin while TBE is monosubstituted, the extremely high selectivity for hydrogenation of TBE shown by (^iPr^PCP)­Ir^I^ is not surprising. Rather, the approximately equal barriers for hydrogenation of COE and TBE when catalyzed by **1-H** ^**+**^ are very noteworthy.

The reaction enthalpy of hydrogenation of CPE is 2.3 kcal/mol more favorable than for COE hydrogenation, but still 3.3 kcal/mol less favorable than for TBE hydrogenation (Table 1). Nevertheless, in a competition experiment, CPE was hydrogenated more rapidly than TBE (Figure 9a). Likewise, cycloheptene (CHpE) has a hydrogenation enthalpy 4.1 kcal/mol less negative than TBE, but is hydrogenated ca. 4 times more rapidly (Figure 9b).

**Figure 9:**
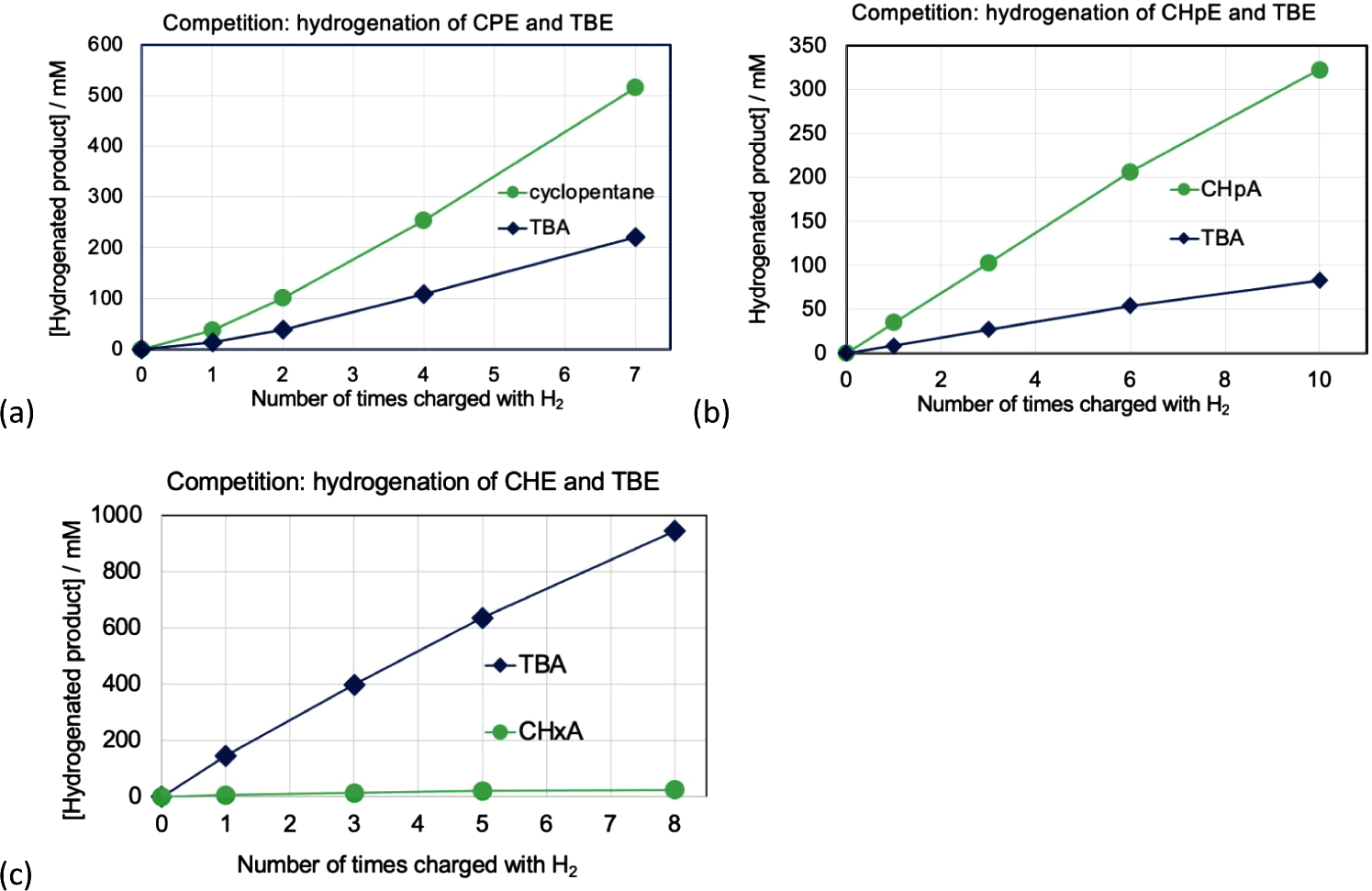
(a) Competition experiments: hydrogenation catalyzed by (^iPr^PCP)­IrH^+^: (a) CPE versus TBE, (b) CHpE versus TBE, and (c) CHxE versus TBE.

CHxE has a thermodynamic driving force for hydrogenation *greater* than that of COE, CHpE, and CPE by 3.5, 2.0, and 1.2 kcal/mol, respectively (Table 2). As noted above, CHpE and CPE are hydrogenated by (^iPr^PCP)­IrH^+^ faster than TBE, while hydrogenation of COE and TBE proceed at essentially the same rate. Yet, despite the significantly more favorable thermodynamics of hydrogenation of CHxE, the rate of CHxE hydrogenation was ca. 300-fold *slower* than that of TBE, corresponding to ΔΔ*G* ^‡^ ≈ 3.4 kcal/mol favoring the hydrogenation of TBE over CHxE (and therefore also ΔΔ*G* ^‡^ ≈ 3.4 kcal/mol favoring hydrogenation of COE over CHxE; Figure 10). Expressed in terms of dehydrogenation, this implies that the barrier to COA dehydrogenation is ≈6.9 kcal/mol less than for CHxA dehydrogenation, although the thermodynamics of COA dehydrogenation are only 3.5 kcal/mol more favorable (Figure 10).

**Table 2:** Relative Thermodynamics of Hydrogenation, Activation Free Energies of Hydrogenation (Kcal/mol) of Cycloalkenes/Cycloalkanes Catalyzed by **1-H** ^**+**^ (Expressed Relative to TBE/TBA) and Inferred Relative Activation Free Energies and Rates of Dehydrogenation

Alkene	ΔΔ*G*° hydrogenation	Relative rate of hydrogenation	ΔΔ*G* ^‡^ hydrogenation	ΔΔ*G* ^‡^ dehydrogenation (inferred)	Relative rate of dehydrogenation of alkane (inferred)
TBE	(0.0)	(1.0)	(0.0)	(0.0)	(1.0)
cyclopentene	+3.3	2	–0.4	–3.7	520
cyclohexene	+2.1	0.003	+3.4	+1.3	0.11
cycloheptene	+4.1	4	–0.8	–4.9	3900
cyclooctene	+5.6	1.0	0.0	–5.6	13000

**Figure 10:**
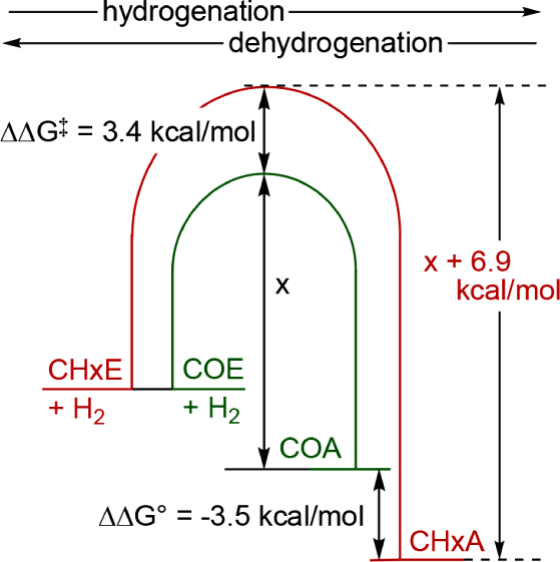
Schematic illustration of relative kinetic barriers of dehydrogenation of CHxA and COA catalyzed by **1-H** ^**+**^ inferred from the kinetics of hydrogenation of CHxE and COE by **1-H** ^**+**^ and the known relative thermodynamics.

### Competition H/D Exchange Catalysis Using **1-H** ^**+**^

Alkane dehydrogenation by molecular transition metal catalysts is generally accepted to proceed via C–H oxidative addition followed by β-H transfer.^13,45,46^ With the goal of probing the C–H activation step, we conducted H/D exchange experiments, again relying on competition experiments to avoid confounding effects due to varying the resting state (particularly as a result of varying the nature of olefinic products) or due to the possible presence of impurities in any particular reagent/substrate.

H/D exchange experiments were based on competitions between various deuterated alkanes versus benzene-d_6_. With H_2_ as the source of hydrogen and the reaction monitored by ^1^H NMR spectroscopy, **1-H** ^**+**^ (generated from **1-HCl** and Na­[BArF^24^]) was found to catalyze H/D exchange with benzene ca. 3-fold more rapidly on a per mol basis than with CHxA (Figure 11a). H/D exchange with *n*-octane proceeded about 2.4 times more rapidly on a per mol basis, with a slight regioselectivity for the terminal position (per C–D bond; Figure 11b).

**Figure 11:**
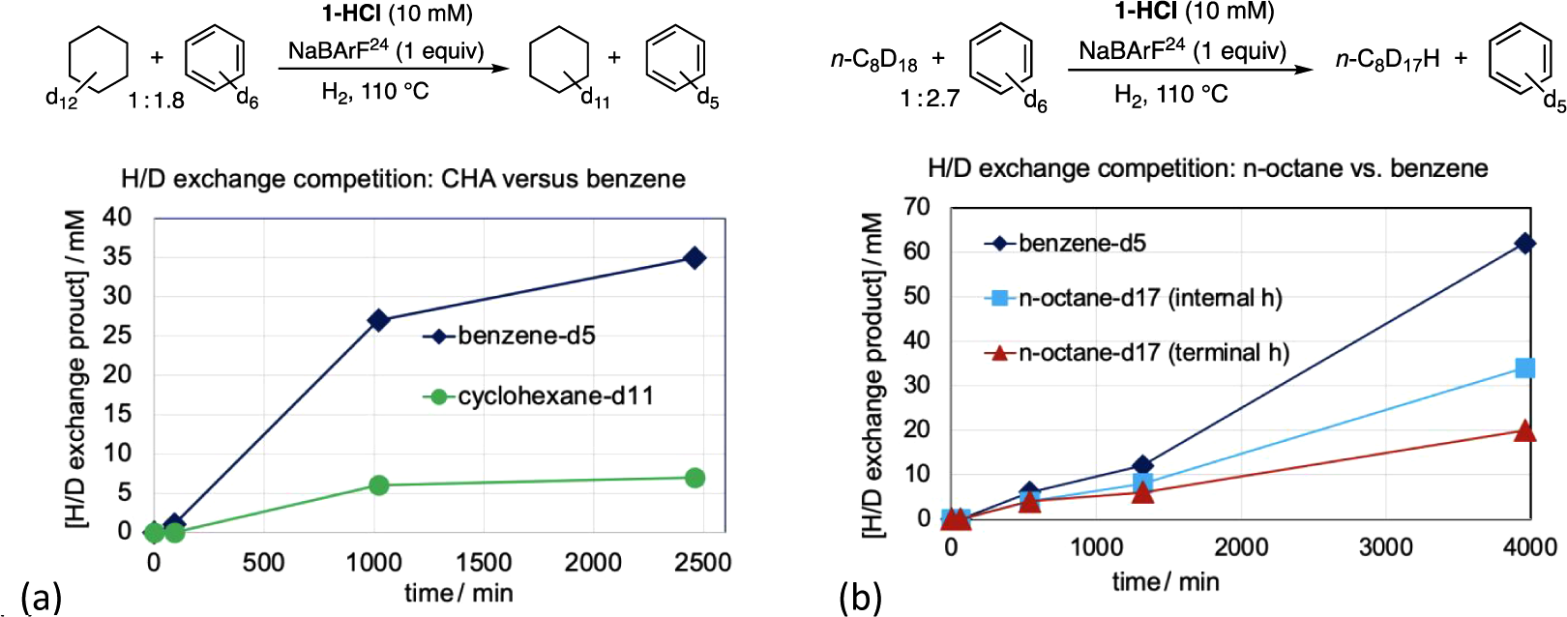
H/D exchange using H_2_ as the source of hydrogen; competitions versus benzene-d_6_: (a) cyclohexane-d_12_ and (b) *n*-octane.

In a striking contrast to the selectivity for H/D exchange of benzene over CHxA, an analogous competition experiment with COA instead of CHxA revealed very high selectivity for COA, over 50:1 on a per mol basis (Figure 12a). Similar results were obtained when dioxane-h_8_ was used instead of H_2_ as the hydrogen source (Figure 12b).

**Figure 12:**
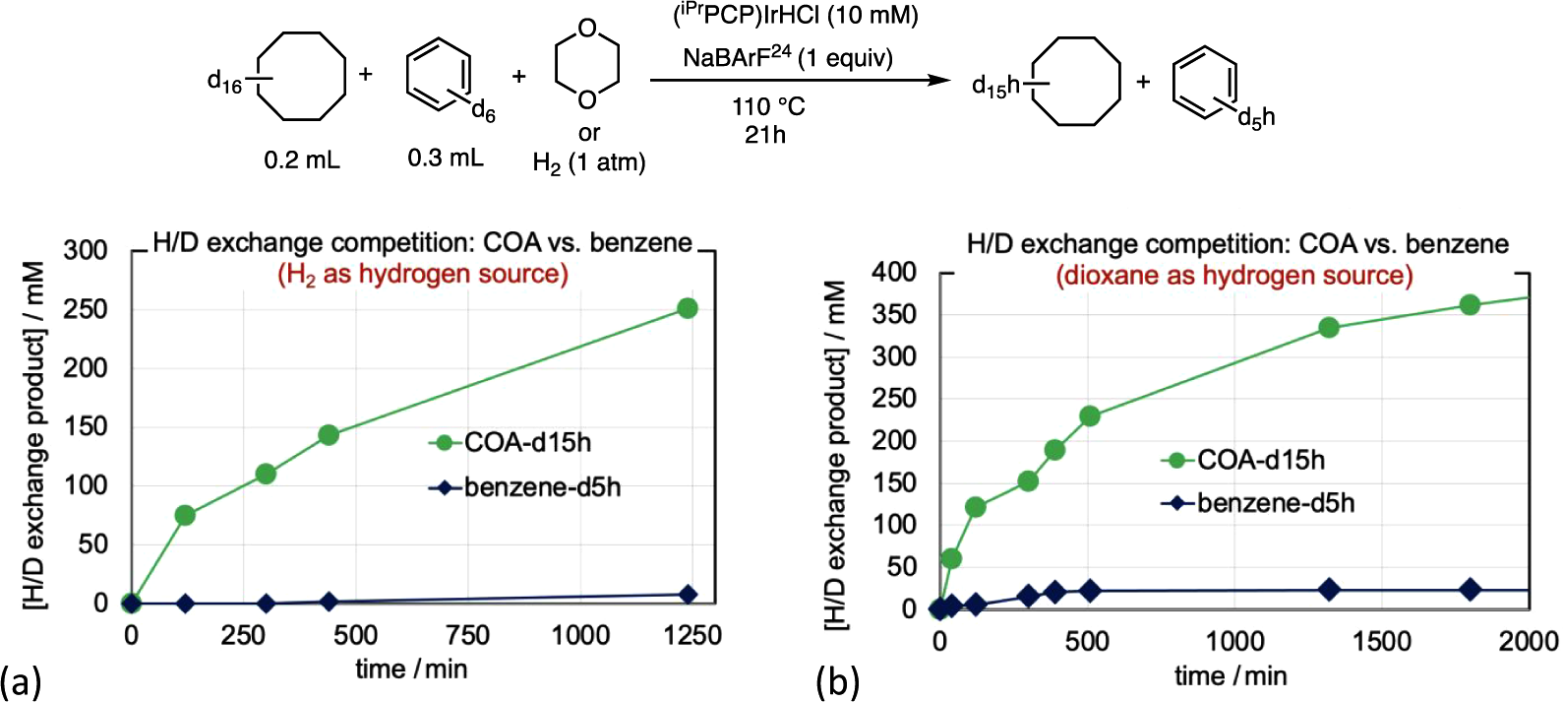
H/D exchange competition studies for cyclooctane-d_16_ using as the source of hydrogen (a) H_2_ and (b) dioxane-h_8_.

Intramolecular H/D exchange competition experiments were also conducted. When H/D exchange of toluene was effected with **1-H** ^**+**^, it was found that the meta- and para-positions were equally reactive on a per-bond basis, while no exchange was observed at the ortho-position presumably due to steric crowding (Figure 13a). The meta- and para-positions are significantly (ca. 8-fold) more reactive than the benzylic position of toluene. In surprising contrast with toluene, however, the benzylic position of ethylbenzene is ca. 1.5-fold more reactive than its meta- and para-positions (Figure 13b); the benzylic position of ethylbenzene is thus about 12-fold more reactive than the toluene benzylic position. Moreover, also surprisingly, the methyl group of ethylbenzene is about 1.5-fold more reactive than the benzylic group of ethylbenzene; the ethylbenzene methyl group is therefore more reactive by a factor of ca. 18 than the toluene (benzylic) methyl group (these comparisons assume that the respective aromatic C–D bonds of toluene and ethylbenzene are approximately equally reactive).

**Figure 13:**
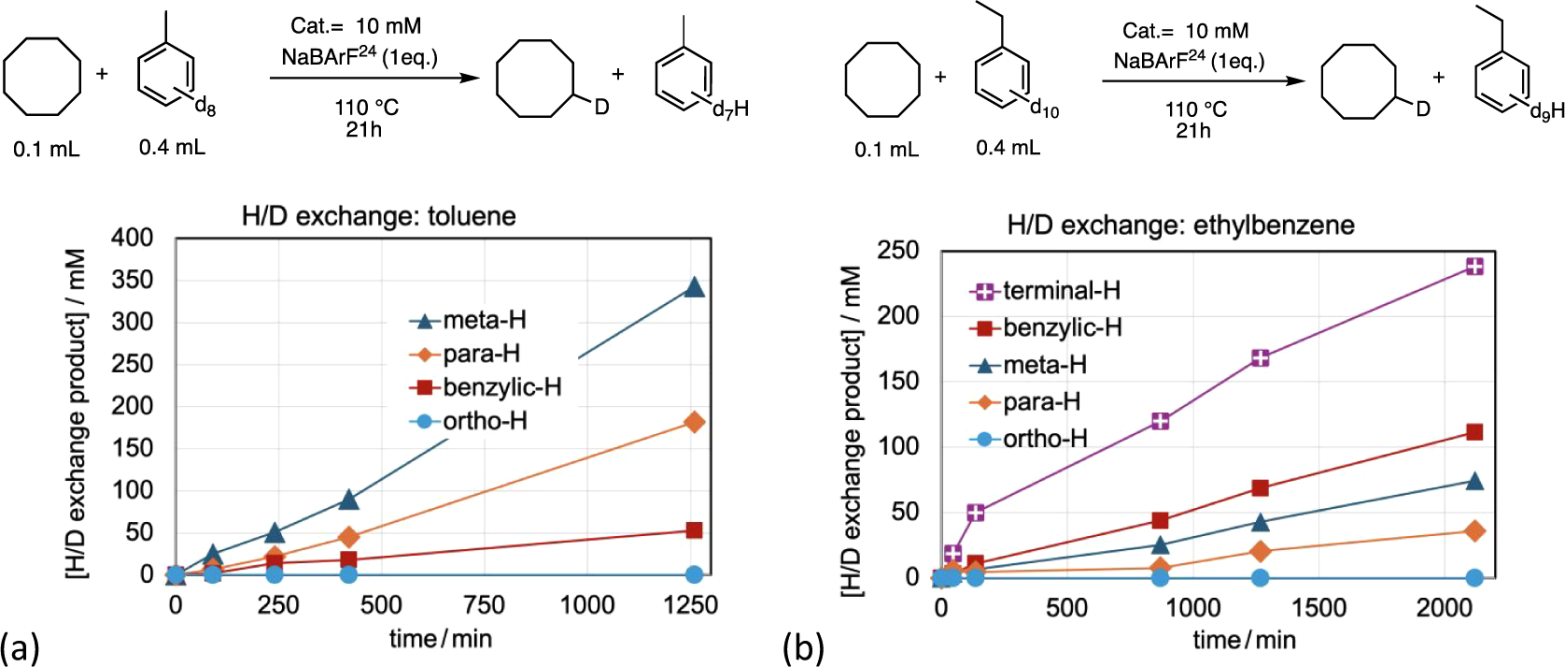
H/D exchange intramolecular competition studies for toluene-d_8_ and ethylbenzene-d_10_ using COA as the source of hydrogen.

### Speciation under Catalytic Conditions

Small crystals were obtained from the NMR tube in which hydrogenation of COE and TBE (Figure 8a) was catalyzed by [**1-H** ^**+**^]­[BArF^24^]. SCXRD afforded the molecular structure of [(^iPr^PCP)­IrH­(COE)­(OH_2_)]­[BArF^24^] (Figure 14), suggestive of a resting state [(^iPr^PCP)­IrH­(COE)^+^] (**1-H­(COE)** ^**+**^). The source of water observed in the crystal structure was not determined, but we suspect it to be the Na­[BArF^24^] used to generate [**1-H** ^**+**^]­[BArF^24^] from **1-HCl**.

**Figure 14:**
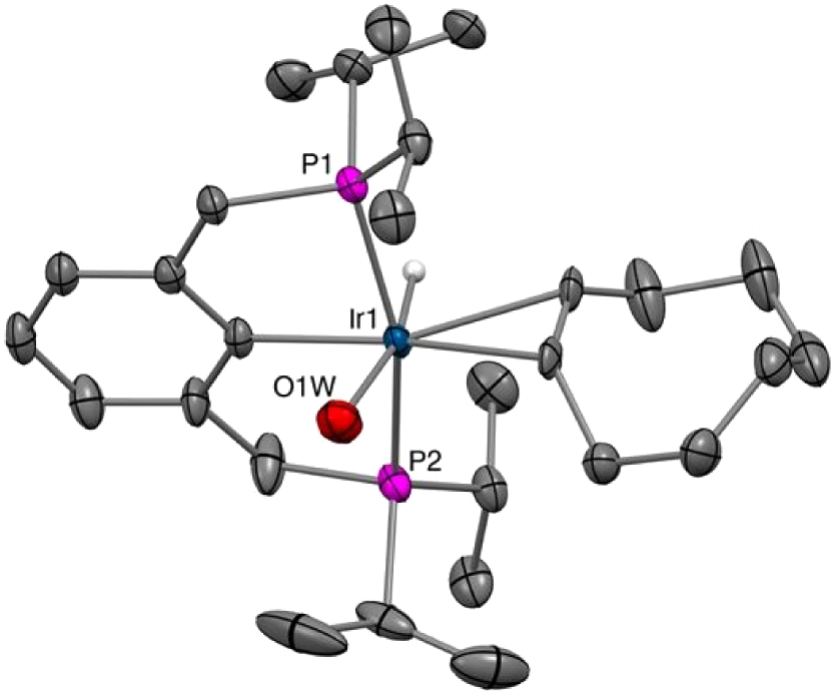
Molecular structure of cationic component of [**1-H­(COE)­(OH**_**2**_ **)**]­[BArF^24^] determined by SCXRD. BArF^24^ anion and hydrogen atoms, except for the hydride ligand, omitted for clarity.

## DFT Computations and Mechanistic Discussion

The behavior of the present system contrasts dramatically with trends generally seen in studies of C–H activation by transition metal complexes. For example, in Bergman’s seminal reports on oxidative addition of alkane C–H bonds, the following relative order of reactivity was found: benzene (4.7) > *n*-alkane 1° (2.7) > CPA (1.6) > CHxA (1.0) > COA (0.09).^4,5,47,48^ Since that time, many systems have been reported in accord with this general trend.^49–62^ By contrast, we find that COA undergoes H/D exchange at least 30-fold faster than benzene in the present system and over 150-fold faster than CHxA. The observation that dehydrogenations of COA and CPA are more facile than that of CHxA is perhaps not surprising as it is consistent with the relative thermodynamics of dehydrogenation – but the magnitude of the differences is much too high to be explained primarily in terms of thermodynamics. This point is reflected in the observation that COE, CHpE, and CPE also undergo hydrogenation much faster than CHxE, or even TBE, in spite of the fact that hydrogenations of the former olefins are thermodynamically less favorable. This very unusual selectivity, observed for both H/D exchange and for dehydrogenation/hydrogenation, has been investigated by computational (DFT) methods.

Geometry optimization and vibrational analyses were carried out in the gas phase using the M06L density functional as implemented in Gaussian-16.^63,64^ For this purpose, the 6-311G­(d,p) basis set was used for the main group elements,^65^ while iridium carried the SDD relativistic effective core potential and associated basis set augmented with one *f* polarization function.^66,67^ All final and solvation energies were obtained in a polarizable continuum representing toluene as a solvent^68^ via single-point calculations on the gas-phase geometries using the M06L, B3LYP-D3BJ,^69^ ωB97X-D,^70^ and PBE0-D3BJ^71,72^ density functionals, employing this time the def2-tzvp basis set on the main group elements and the def2-qzvp basis set with associated ECP on Ir.^73,74^ The enthalpy and Gibbs free energy terms were obtained from the gas-phase calculations^75^ at 298.15 K and adjusted to 1 M for hydrocarbon reactants and products.^76,77^ The magnitudes of barriers calculated using B3LYP-D3BJ and ωB97X-D were comparable, and intermediate between the M06L and PBE0-D3BJ values (Tables S26–S39), but the trends were the same for the entire range of alkanes investigated with all four functionals. We base the discussion in the text on the B3LYP-D3BJ results, and we give the full results including the solvation effects in the Supporting Information. The ^iPr^PCP ligand can define several conformations; for practical considerations, we limited the calculations to the conformer observed for [**1-H­(COE)­(OH**_**2**_]­[BArF^24^] in Figure 14.

### C–H Bond Addition

The d^6^ **1-H** ^**+**^ fragment is calculated to have a closed-shell electronic state and a bent (Ir–C–H angle = 96.8°) geometry (Scheme 5) in accord with previous reports^78–82^ of four-coordinate d^6^ complexes. Alkane addition to **1-H** ^**+**^ can proceed with the hydrogen atom of the C–H bond undergoing cleavage oriented either away from or toward the hydride ligand of **1-H** ^**+**^ (leading toward trans or cis hydrides, respectively). The two orientations are illustrated in Scheme 5 and designated to be part of mechanisms **A** and **B**, respectively. We first discuss mechanism **A**.

**Scheme 5:**
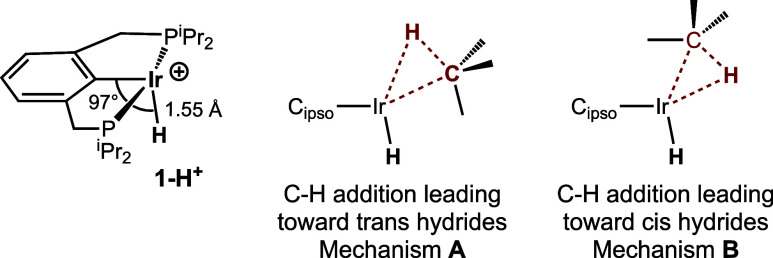
C–H Addition Pathways Defining Mechanisms **A** and **B**

### Mechanism A: C–H Bond Addition

The calculated transition states (TSs) for C–H addition to **1-H** ^**+**^ on pathway **A** (**TS1**) for some of the alkanes investigated are illustrated in Figure 15, and their energies relative to the separated reactants are given in Table 3. In all cases, the incipient Ir–H and Ir–C bonds are found to be nearly fully formed in the TS (C–H atoms shown in red). The incipient Ir–H bond distances are essentially equal (within 0.02 Å) to the Ir–H bond distance of the hydride ligand already present prior to C–H addition. Likewise, the incipient Ir–C bond is quite short, ca. 2.2 Å, within the range of a typical iridium alkyl C–H bond length (ca. 2.2 Å). Accordingly, the C–H distance (>1.75 Å) of the bond undergoing cleavage in the TS is far greater than that of an actual C–H bond. Thus, the TSs for C–H addition to **1-H** ^**+**^ are quite “late”, and have substantial Ir­(V) character.

**Figure 15:**
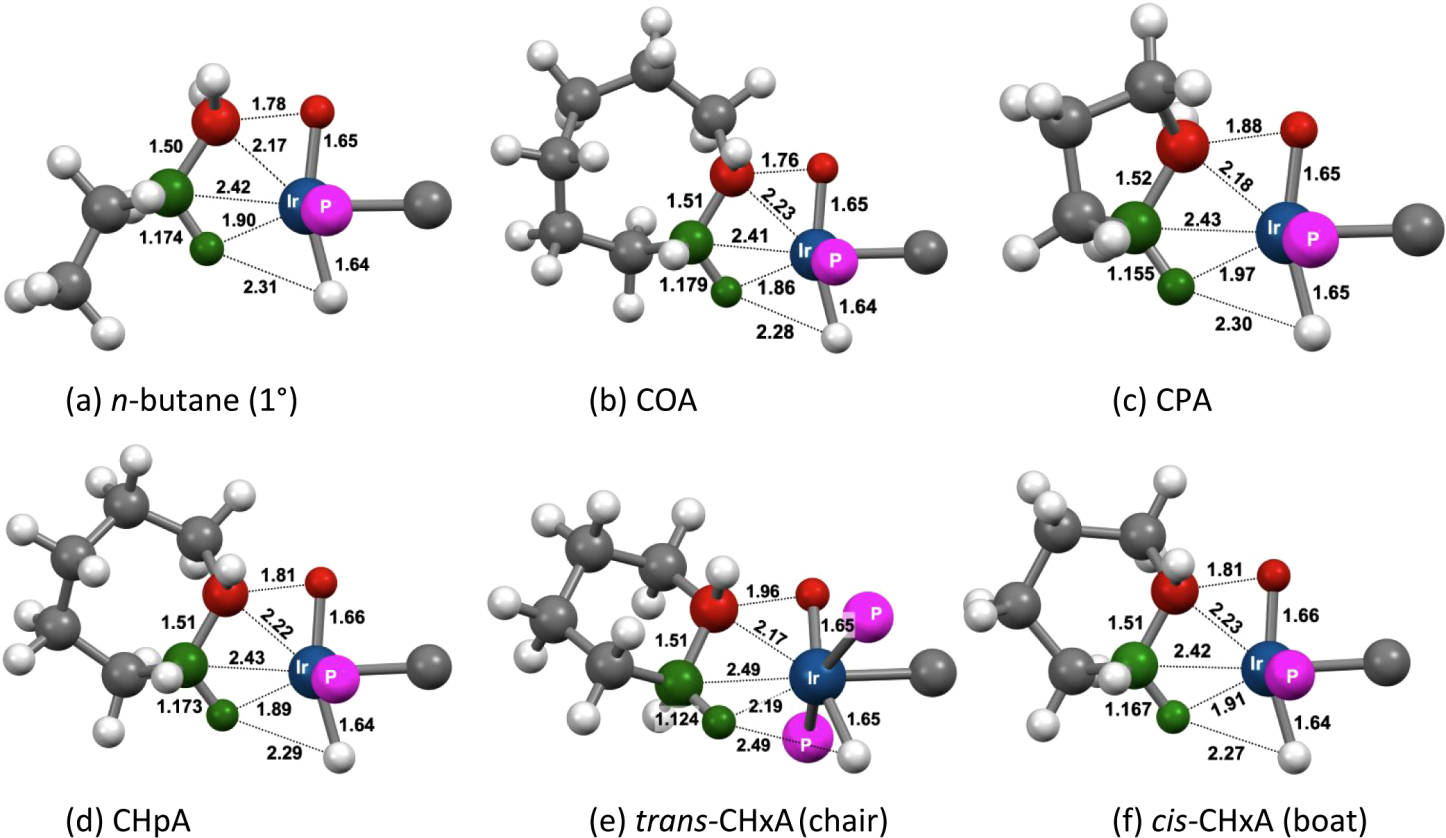
Calculated geometries of **TS1** for C–H bond addition of various alkanes to **1-H** ^**+**^. Atoms of C–H bond undergoing addition in red and the atoms of the β-agostic C–H bond in green.

**Table 3:** Calculated^a^ Free Energies of TSs and Products of C–H Addition to **1-H** ^**+**^ and Subsequent β-C–H Transfer to Hydride Ligand (Mechanism **A**) in kcal/mol Relative to Free Alkane and **1-H** ^**+**^

	C–H Addition		Transfer of β-H to hydride		Free alkane	
Alkane	**TS1** (C–H addition)	**1-(H)_2_R^+^** (C–H addition prod.)	**TS2** (β-H-transfer)	**1-H(H_2_)(alkene)^+^** (β-H transfer prod.)	Ring strain^b^	Δ*H* Eclipsed Conformer^c^
TBA	28.0	27.8	31.2	12.3	--	2.0
*n*-butane	27.6	26.8	29.9	9.0	--	2.4
CPA	30.6	29.9	32.7	9.2	6.2	0.0
CHxA (cis)	32.9	31.8	34.0	13.0	0.1	6.9
CHpA	26.8	25.2	26.8	4.7	6.2	0.3
COA	27.4	24.9	25.2	6.4	9.7	1.9
CDA (cis)	31.4	29.5	30.0	4.9	4.1	
CDA (trans)	31.7	29.4	28.0	5.0		
CHxA (trans)	35.6	35.5	49.5	38.8^d^	0.1	0.2

a B3LYP-D3BJ (toluene solvent continuum).

b Free cycloalkane.^43^

c Calculated values for free (unbound) alkane constrained with one eclipsed interaction; cis-C–H bonds eclipsed for all cycloalkanes except the value given for “CHxA (trans)”.

d Ir-bound *trans*-cyclohexene.

The most noteworthy aspect of the TSs for C–H addition is an agostic interaction with the β-C–H bond (corresponding atoms shown in green in Figure 15). This interaction appears to be very strong, for both linear and cyclic substrates, as indicated by the pronounced elongation of the β-C–H bonds as well as the short β–H–Ir bond distance. Respective values, for example, in the case of *n*-butane (Figure 15a), are 1.174 Å (versus 1.09 Å for a typical alkyl C–H bond) and 1.90 Å (M–H bond distances of agostic C–H bonds are considered to range between 1.8 and 2.3 Å^83^).

In the case of cyclohexane, two TSs are shown in Figure 15. In the first, cyclohexane adopts a chair conformation with an equatorial C–H bond undergoing addition. Agostic bonding in this TS involves a β-C–H bond that is also at an equatorial site of the ring, therefore leading to a stereoconfiguration in which the C–H bond undergoing addition and the agostic β-H are at trans positions of the six-membered ring (*trans-*CHxA; Figure 15e); this is in contrast to the other cycloalkanes in which Ir and the agostic β-hydrogen are mutually cis (Figures 15b-d). The metrics of the Ir–C–H β-agostic unit in *trans-*CHxA indicate an agostic interaction much weaker than for the other alkanes. In the second TS for cyclohexane, the ring is in a boat conformation and the C–H bond undergoing cleavage and the agostic β-C–H bond are mutually cis (*cis-*CHxA; Figure 15f). In this TS, the metrics of the agostic Ir–C–H unit are very similar to those found in the other alkanes (Figure 15a–d). Thus, CHxA can “choose” either a strong agostic interaction, similar to that found with the other alkanes investigated, but accompanied by severe conformational strain (the “boat” conformation), or alternatively, an unstrained (chairlike) conformer with a weak agostic interaction. The calculations predict the highly strained *cis*-CHxA **TS1** to have a significantly lower energy than *trans*-CHxA (Table 3) unequivocally highlighting the importance of the agostic interaction in driving C–H addition in the given system.^84^

An effective agostic interaction in **TS1** clearly requires an eclipsed relationship of the H atoms that are geminal to the agostic C–H bond and the C–Ir bond. This is necessarily unfavorable for all alkanes, but it is expected to be most severe for cyclohexane. In support of this proposal, calculations of the free cycloalkanes with a fully eclipsed HCCH unit (Table 3) indicate an energetic cost of 6.9 kcal/mol for CHxA; this is much greater than that of *n*-butane (2.4 kcal/mol). In contrast, the energies of eclipsed conformers of CPA, CHpA, and COA are lower than that of *n*-butane. Not surprisingly, therefore, the energy of **TS1** for *cis*-CHxA (35.6 kcal/mol) is computed to be higher than for all other alkanes in Table 3 (26.8–31.7 kcal/mol).

For all substrates, Intrinsic Reaction Coordinate (IRC) analyses show **TS1** to lead to the intermediate (**i1**) **1-(H)**_**2**_ **R** ^**+**^, having (after full geometry optimization) an approximately pentagonal-bipyramidal geometry in which the incipient C–H bond has been completely cleaved (C–H distance > 2.0 Å, Figure 16) and one of the five equatorial sites is occupied by the β-agostic C–H bond. The metrics are indicative of even stronger β-agostic C–H bonds than those found in the respective TSs (the agostic Ir–H distances are shorter and the agostic C–H bonds are longer).

**Figure 16:**
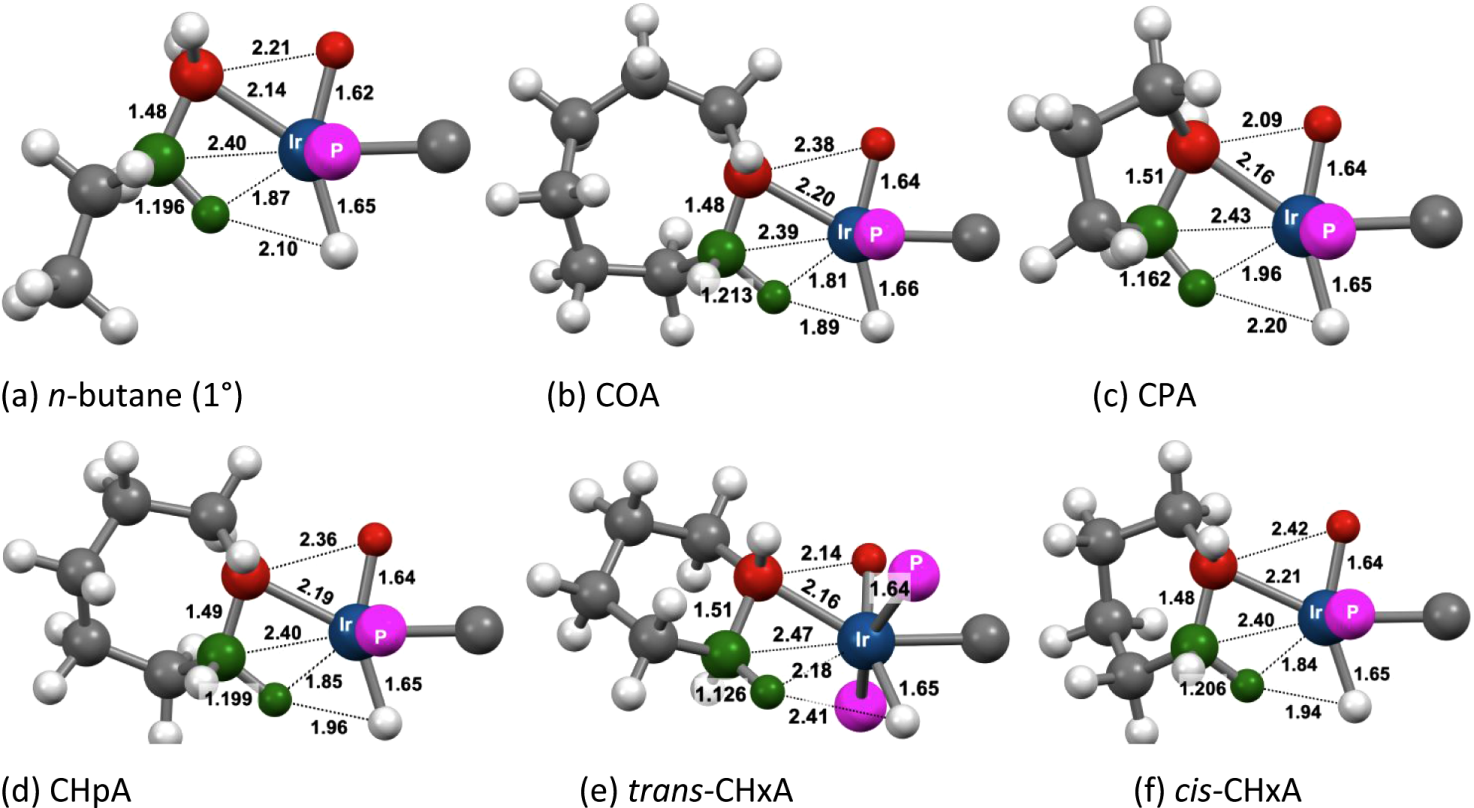
Calculated geometries of products (**1-(H)**_**2**_ **R** ^**+**^) of C–H addition to **1-H** ^**+**^. Atoms of added C–H bond in red and the atoms of the β-agostic C–H bond in green.

Consistent with the TSs of C–H addition (**TS1**) being “very late”, the free energies of the products of addition are nearly equal to those of the TSs (Table 3). The spans of ca. 10 kcal/mol in Δ*G* ^‡^ and 12 kcal/mol in Δ*G*° for the alkanes investigated are far greater than those implied by previously reported experimental studies,^4,5,47–50,59^ of cycloalkane addition (e.g., a range of 12-fold between CPA, CHxA, and COA at −60 °C, corresponding to a difference of only ca. 1.1 kcal/mol). Perhaps even more notably, while COA was found to be the *least* reactive cycloalkane in such systems, it is calculated to be the *most* reactive cycloalkane in the present system. These differences can be well explained, as discussed above, in terms of ring strain and the importance of the agostic interaction in the present system. Conversely, in the case of “classical” C–H activating transition metal systems, where no ancillary agostic interaction is involved, the addition of a metal center to the ring only *increases* the strain associated with eclipsed or intra-annular interactions.

### Mechanism A: β-H Transfer

For catalytic alkane dehydrogenations operating via an initial C–H bond addition, the mechanism is typically assumed to proceed by a conventional of β-H transfer step, to afford a coordinated olefin and a second M-H bond. Our calculations, however, predict that the agostic β-C–H bond in the present products of α-C–H addition, instead undergoes a transfer of the β-hydrogen to the adjacent hydride ligand (**TS2**, Figure 17). We will refer to this step as a β-H-to-hydride transfer (BHHT). Such β-H transfers to a ligand are not common, but they are not unprecedented.^85–88^ While no intermediate with a new Ir–H bond is formed during this process, the Ir–(β-H) distances (ca. 1.71 Å) do, however, suggest a significant degree of Ir–H bonding in these TSs (Figure 17). Perhaps the closest comparison to such a TS is with oxidative hydrogen migrations^89–91^ or σ-CAM pathways,^92,93^ in which the TS typically has the character of a C–H addition product, but rather than being an energy minimum, it is a TS leading to a dihydrogen complex.

**Figure 17:**
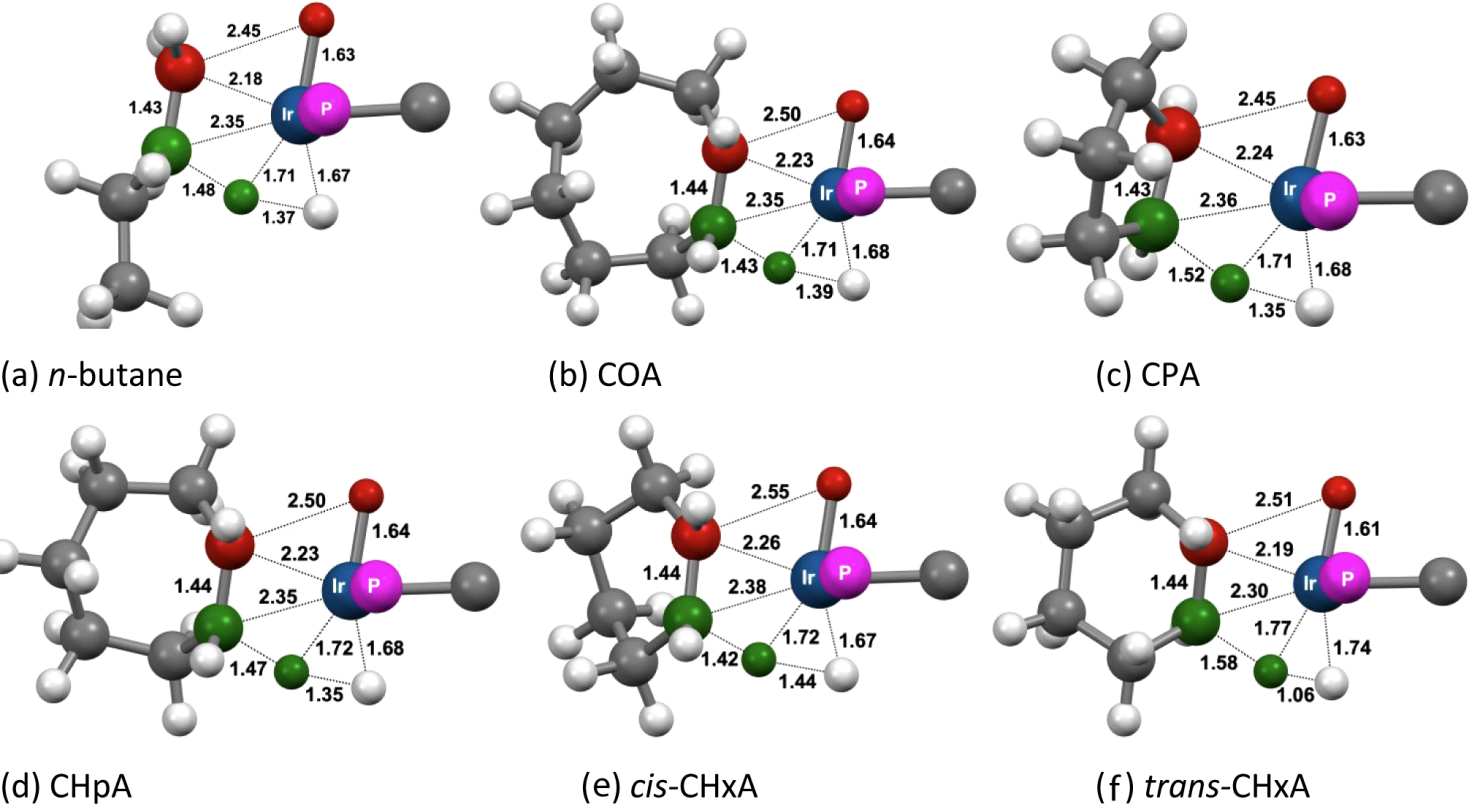
Calculated geometries of **TS2** for β-C–H transfer (from carbon to hydride). Atoms of the first C–H bond addition step in red and the atoms of the β-hydride transfer step in green.

As stressed above, the TSs for C–H addition (**TS1**) have geometries and energies remarkably similar to the C–H addition products (**i1**). Those same C–H addition products have, in turn, geometries and energies very similar to the subsequent TSs (**TS2**) for β-H-to-hydride transfer (BHHT). The geometric relationships are highlighted in Figure 18 using cyclopentane as an example (see Table 3 for free energies). Given how shallow these calculated minima are on the potential energy surface (PES)^94^ and, in some cases, nonexistent on the enthalpy and Gibbs free energy surface, we can infer that they do not represent true intermediates in the sense of species with a finite lifetime. Rather, the C–H addition and β-H transfer to the hydride ligand can be viewed, in effect, as components of an asynchronous concerted process,^95,96^ connecting weakly bound alkane σ-C–H complex precursors (**1-H­(RH)** ^**+**^) with the corresponding olefin dihydrogen complex intermediates **1-H­(alkene)­(H**_**2**_ **)** ^**+**^ (**i2**). Complexes **i2** have an approximately octahedral geometry with mutually trans hydride and η^2^-H_2_ ligands.^97^ Interestingly, the H_2_ ligand of **i2** (e.g., Figure 18d) is oriented perpendicular to the plane of the reaction coordinate of **TS2**, yet it is reached directly by the IRCs originating from **TS2**.

**Figure 18:**
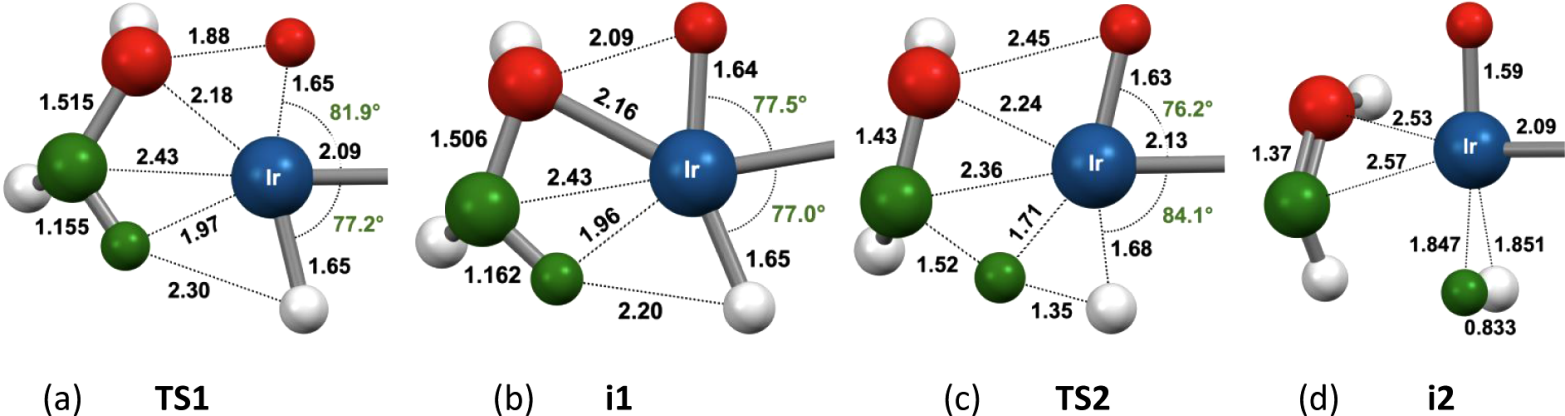
Illustration of the geometric similarity of the stationary points identified on the PES of dehydrogenation of cyclopentane by **1-H** ^**+**^ by mechanism A. For clarity, only atoms in the coordination sphere of the metal and approximately in the plane perpendicular to the P–P axis (and H atoms bound to the key carbons undergoing C–H addition and β-H transfer) are shown.

Following the formation of **1-H­(alkene)­(H**_**2**_ **)** ^**+**^ (**i2**), several different steps appear to be plausible. The formation of **1-H­(alkene)­(H**_**2**_ **)** ^**+**^ from alkane and **1-H** ^**+**^ is calculated to be endergonic in all cases (Table 3 and Figure 20); therefore, the reverse of its formation would be rapid. Rotation of H_2_ around the (η^2^-H_2_)–Ir axis presumably has a very low barrier. The formation of **1-H­(alkene)­(H**_**2**_ **)** ^**+**^, followed by a rapid back-reaction, would therefore lead to exchange between the hydride of **1-H** ^**+**^ and alkane, providing a pathway for hydrogen isotope exchange (HIE).^98^ Notably, the H/D exchange occurs at the β-position rather than at the site of actual (α) C–H oxidative addition (Figure 19).

**Figure 19:**
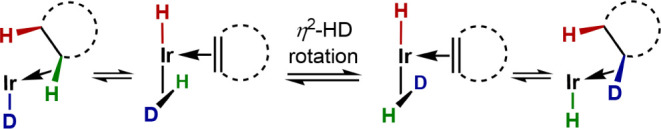
Schematic illustration of mechanism **A** for C–H/Ir–D exchange.

As illustrated for the reaction of COA in Figure 20, the loss of H_2_ from **1-H­(COE)­(H**_**2**_ **)** ^**+**^ is calculated to be approximately ergoneutral. We have been able to locate a TS for the loss of H_2_ from only one of the **1-H­(alkene)­(H**_**2**_ **)** ^**+**^ complexes (alkene = 1-butene) but, as expected, the barrier for H_2_ loss is very low (Δ*G* ^‡^ = 5.0 kcal/mol) and thus H_2_ loss is presumably facile and reversible. We infer that this is the case for all the alkanes investigated.

**Figure 20:**
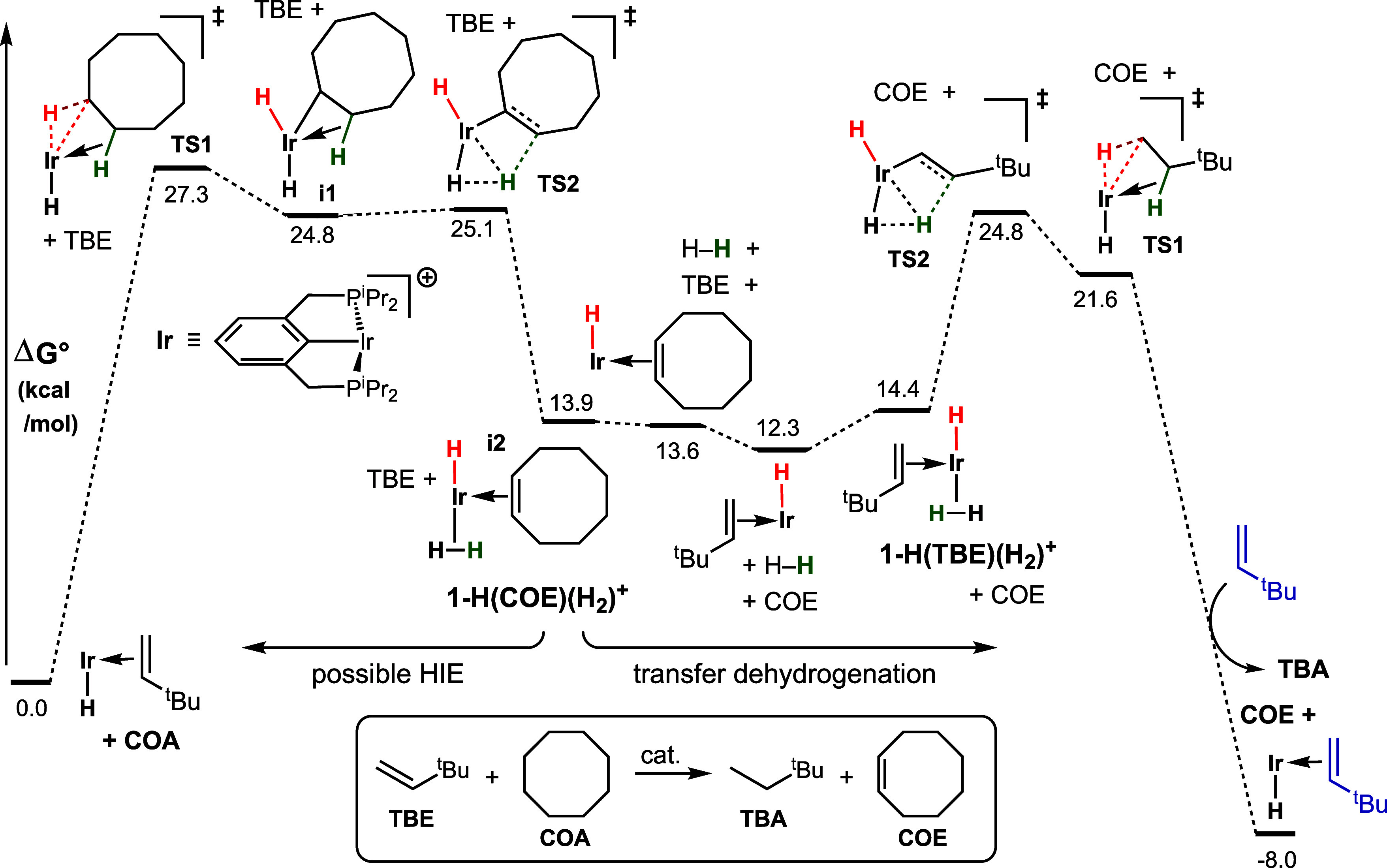
Free energy profile and schematic of full catalytic cycle calculated for COA/TBE transfer-dehydrogenation. (B3LYP-D3BJ in toluene continuum, 298 K, 1 M hydrocarbons, 1 atm H_2_, statistical corrections made for number of possible microstates, based on permutations of C–H bonds.^36^

If H_2_ is lost reversibly from **1-H­(alkene)­(H**_**2**_ **)** ^**+**^, the H_2_ loss process would have no obvious effect on any of the experimental observations including HIE. However, H_2_ loss results in a vacant coordination site, which could permit an associative exchange of alkenes. Indeed, as discussed in the previous section (Experimental Results), experimentally we have shown that complexes **1-H­(alkene)** ^**+**^ undergo rapid exchange between free and bound alkenes. If alkene exchange does occur prior to reassociation of H_2_, followed by hydrogenation of the incoming alkene to give the corresponding alkane (the reverse of the alkane dehydrogenation), it provides a full pathway for transfer dehydrogenation (with COA/TBE as the H-donor/H-acceptor couple in Figure 20). We also cannot exclude an alternative catalytic pathway for alkene exchange, in which alkene is lost directly from **1-H­(alkene)­(H**_**2**_ **)** ^**+**^ and the incoming acceptor replaces it (COE and TBE in Figure 20). Loss of alkene, however, is calculated to have a greater barrier than loss of H_2_ (e.g., Δ*G*° = 7.1 kcal/mol for vertical COE dissociation), compared with Δ*G* ^‡^ = 5.0 kcal/mol for loss of H_2_. But regardless of whether alkene exchange occurs directly or via loss of H_2_ (as in Figure 20), if exchange is rapid relative to the back reaction for formation of **1-H­(alkene)­(H**_**2**_ **)** ^+^, it would not affect the overall rate of transfer dehydrogenation (or HIE). The present study is focused primarily on the initial C–H bond cleavage and β-H transfer steps, i.e., the formation of **1-H­(alkene)­(H**_**2**_ **)** ^**+**^ from **1-H** ^**+**^ and alkane. In view of the very unusual selectivity observed, it is clear that these steps, and not olefin exchange, determine the rates, including relative rates, of dehydrogenation and HIE.

In the mechanism discussed so far, the C–H bond undergoing addition is positioned trans to the hydride ligand initially present in **1-H­(RH)** ^**+**^, while the agostic bond is cis to that hydride, as summarized in Figure 20 (mechanism **A**). In this mechanism, HIE occurs selectively between the originally present hydride ligand and the β*-*C–H bond. Such a pathway, however, is not possible for species that cannot feasibly undergo 1,2-dehydrogenation, e.g., for aromatic C–H bonds or for the benzylic C–H bond of toluene. Yet, as described in the experimental results section above, these molecules are in fact observed to undergo HIE, although somewhat slowly. In this context, we will now consider mechanism **B** (Figure 21) in which the hydride resulting from C–H addition is positioned cis to the hydride initially present in **1-H** ^**+**^, thus allowing exchange between the α-C–H bond and the hydride of **1-H** ^**+**^.

**Figure 21:**
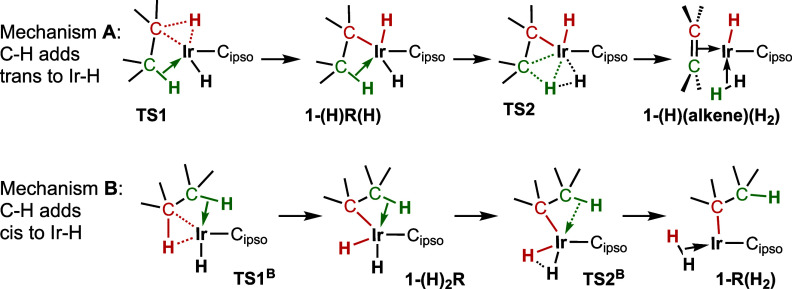
Comparison of alkane dehydrogenation by mechanisms **A** and **B**.

### Mechanism B: C–H Bond Addition

Representative TSs for C–H bond addition by mechanism **B** are illustrated in Figure 22 and free energies are given in Table 4. As found for mechanism **A**, the TSs for alkane C–H addition (**TS1** ^**B**^) are characterized by large degrees of C–H bond cleavage and Ir–H bond-making and strong β-agostic interactions. Based on metric parameters, the agostic interactions, although relatively strong, appear to be slightly weaker than those present in the corresponding TSs of Mechanism **A** (i.e., the C–H distances are slightly shorter and the Ir–C and Ir–H distances slightly longer; Figure 22). This can be readily attributed to the strong trans-influence of the parent hydride ligand that is exerted on the agostic bond in **TS1** ^**B**^.^99^ Despite the weaker agostic bonds, Table 4 shows the energies of **TS1** ^**B**^ to be comparable to those of **TS1** in Table 3. This is unsurprising because **TS1** in Mechanism **A** yields an Ir–H bond trans to the initial hydride; the resulting trans-dihydride configuration is generally unfavorable.

**Figure 22:**
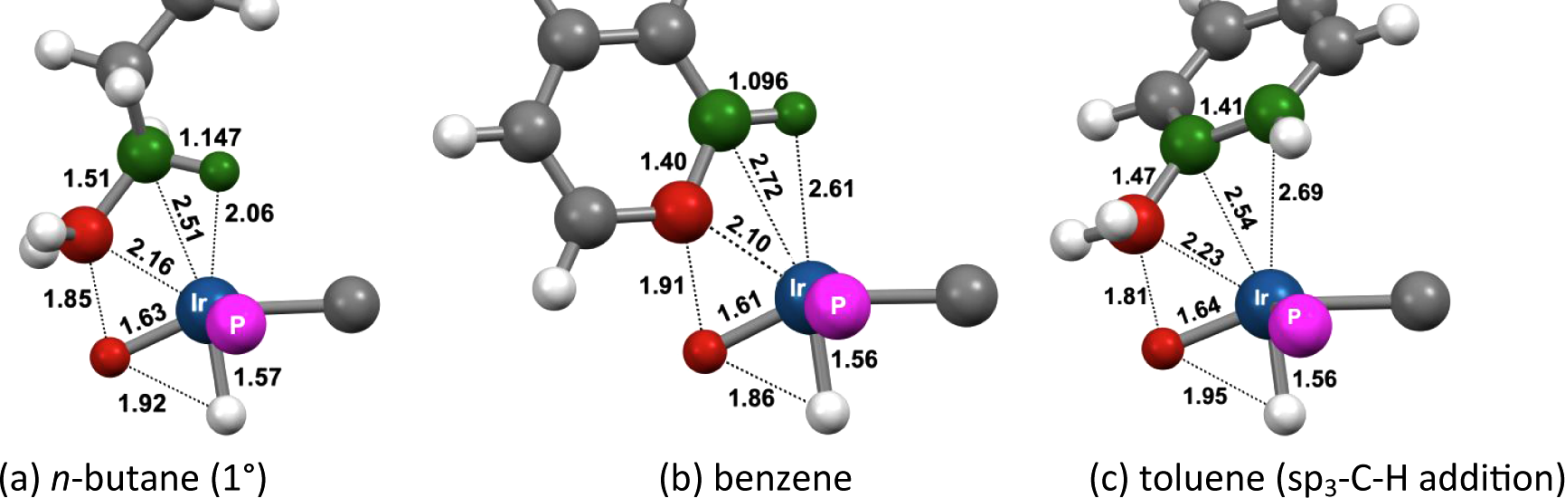
Calculated geometries of TSs for C–H addition via mechanism **B** (**TS1** ^**B**^). Atoms of C–H bond undergoing addition in red and the atoms of the β-agostic C–H bond (or C1–C2 π-system for toluene) in green.

**Table 4:** Calculated Free Energies^a^ (kcal/mol) of TSs and Products of C–H Addition to **1-H** ^**+**^ (Relative to Free Hydrocarbon and **1-H** ^**+**^) for Mechanism **B**

	C–H Addition		Reductive η^2^-H_2_ formation	
Hydrocarbon	**TS1^B^** C–H addition	**i1^B^** cis-**1-(H)_2_R^+^**	**TS2^B^** (H–H-bond formation)	**i2^B^** **1-(alkyl)(H_2_)^+^**
*n*-butane	29.5	29.7	36.4	21.3
CPA	33.0	33.2	39.0	24.7
COA	26.0	26.3	35.4	25.1
benzene	28.5	28.5	31.4	13.4
toluene	31.4	30.3	36.8	20.9

a B3LYP-D3BJ (toluene solvent continuum).

For the addition of benzene, a β-C–H bond is oriented toward the metal in **TS1** ^**B**^ (Figure 22b). However, the corresponding C–H (1.096 Å) and Ir–H (2.61 Å) bond distances indicate that any agostic interaction is at best minimal. Thus, although the reaction of benzene involves the addition of a C­(sp^2^)–H bond (typically, significantly more favorable than sp^3^), the computed energy of **TS1** ^**B**^ for benzene is similar to that for the addition of the 1° C–H bond of *n*-butane (Table 4). Interestingly, in the case of toluene, C­(sp^3^)–H addition proceeds via a TS with η^3^-allyl character, in which the C1–C2 π-system seems to play a role analogous to that of the agostic C–H bond for alkane C–H addition (Figure 22c). Consistently, the energy of **TS1** ^**B**^ for toluene is also close to that of *n*-butane.

As in the case of mechanism **A**, the products of C–H addition of mechanism **B** are geometrically and energetically very similar to those TSs that lead to them, as illustrated for *n*-butane in Figure 23. However, unlike pathway **A**, pathway **B** results in the two hydride ligands positioned mutually cis in the Ir­(V) product **1-R­(H)**_**2**_^**+**^ (**i1** ^**B**^). This would seem to offer the possibility of facile reductive formation of coordinated H_2_, which, if reversible, could result in HIE between **1-H** ^**+**^ and both alkanes and aromatic C–H bonds.

**Figure 23:**
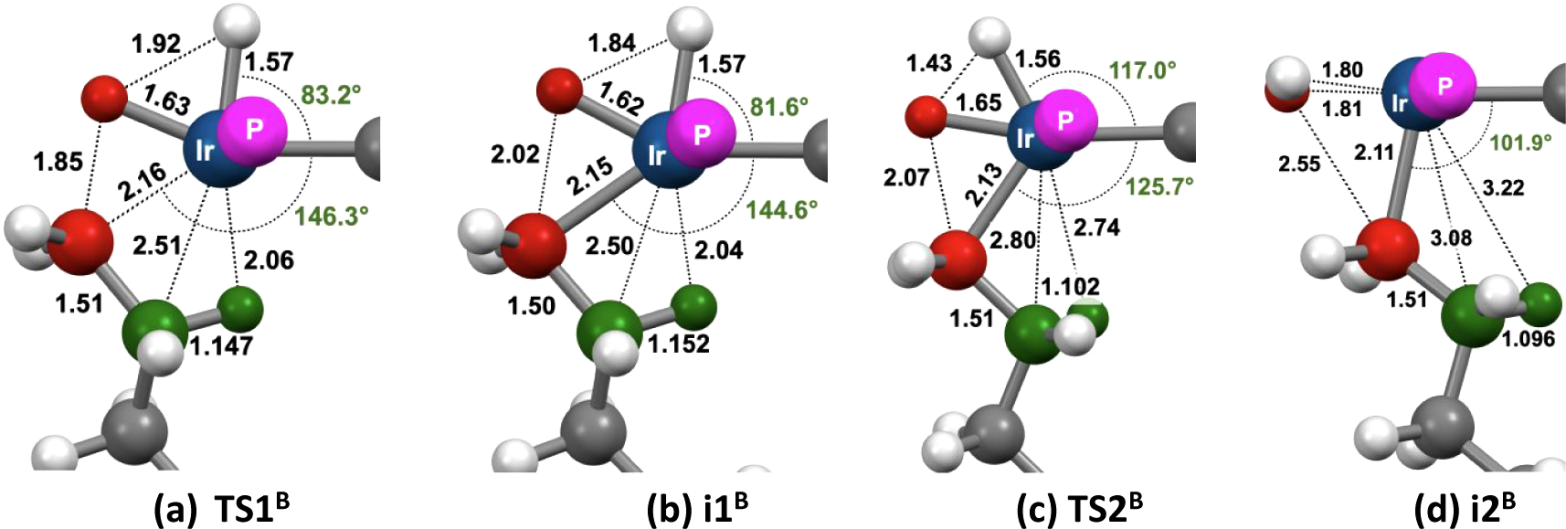
Dehydrogenation of *n*-butane (1° position) with **1-H** ^**+**^ via mechanism **B**. (a, b) TS and product for C–H addition; (c, d) TS and product for reductive H–H bond formation.

### Mechanism B: η^2^-H_2_ Formation

For all substrates studied, reductive H–H bond formation from *cis-* **1-R­(H)**_**2**_^**+**^ is computed to give a five-coordinate d^6^-Ir­(III) dihydrogen complex **1-R­(H**_**2**_ **)** ^+^ (**i2** ^**B**^) having a square pyramidal geometry with the R-group at the apical position and the H_2_ ligand oriented perpendicularly to the Ir–R bond. This square-pyramidal geometry is commonly observed among d^6^-ML_5_ complexes lacking a single π-donor ligand (which adopt a distorted TBP or “Y” geometry).^100–102^ It is particularly favorable in this case because the two strong-trans-influence groups in **i2** ^**B**^, the alkyl (R) and the PCP ipso-carbon, are mutually cis and each is trans to either an empty coordination site or a very weak trans-influence ligand, H_2_.^24,101–103^ Obviously, agostic bonding is not feasible in **1-R­(H**_**2**_ **)** ^+^, as illustrated for *n*-butane in Figure 23d. Remarkably, the calculations identify a TS for H–H bond formation, **TS2** ^**B**^, that connects reactant **1-R­(H)**_**2**_^**+**^ (**i1** ^**B**^) directly with **1-R­(H**_**2**_ **)** ^+^ (**i2** ^**B**^) even when R is an alkyl group that affords an agostic bond in **1-R­(H)**_**2**_^**+**^. In such a case, the IRC of the full transformation comprises three segments: (i) initial rotation of the alkyl group in **1-R­(H)**_**2**_^**+**^ that disconnects the agostic bond; (ii) H–H bond formation by motion of the hydride trans to R in the direction of the hydride cis to R; and (iii) final motion of the alkyl and H_2_ ligands to attain the square pyramidal geometry. Figure 23c shows the large extent of agostic bond loss in **TS2** ^**B**^. Accordingly, for *n*-butane reductive H–H bond formation, starting from **1-R­(H)**_**2**_^**+**^ (**i1** ^**B**^), a significant barrier (Δ*G* ^‡^ = 6.7 kcal/mol) is encountered (Table 4). An even greater barrier is computed in the reaction of COA (9.1 kcal/mol). A substantial barrier is also computed for the reaction of toluene (6.5 kcal/mol) in which there is no agostic bond, but **TS2** ^**B**^ requires loss of an η^3^-allyl interaction. In contrast, the barrier for H–H bond formation following C–H addition of benzene is very low (Δ*G* ^‡^ = 2.9 kcal/mol).

Thus, alkane C–H addition is found to be comparably facile via pathways **A** and **B**, with both pathways involving strong assistance by a β-agostic interaction. The resulting coordination geometries, however, necessitate distinctly different subsequent reactions. Pathway **B** allows the reductive formation of bound H_2_, and thereby enables direct HIE between the α-C–H bond that undergoes addition and the hydride ligand of **1-H** ^**+**^. Pathway **A** allows the transfer of H from the β-agostic C–H bond to the **1-H** ^**+**^ hydride ligand (BHHT). Surprisingly perhaps, the BHHT TS (**TS2** ^**A**^) is very similar in energy and even geometry to the TS for C–H addition (**TS1** ^**A**^), whereas the TS for H–H reductive bond formation (**TS2** ^**B**^) is significantly higher in energy than **TS1** ^**B**^. Simplistically, the agostic bonding can be viewed to activate the β-C–H bond toward BHHT in pathway **A** while, in contrast, it inhibits reductive H–H bond formation (**TS2** ^**B**^) in pathway **B**.

Only in the case of pathway **A** do the steps described above lead to dehydrogenation to give olefin (coordinated to the iridium center). In the case of pathway **B**, β-H transfer after C–H addition is in principle possible, but if it were to occur after the H–H reductive bond formation (and subsequent H_2_ loss), the overall barrier for the reaction would of course be at least as high as the TS for the H–H reductive bond formation step; the computational results therefore strongly suggest that pathway **B** is unfavorable for the alkanes investigated.^104^

For C–H activation of substrates such as toluene (benzylic position) or benzene, pathway **A** is not a feasible option for HIE. Either there is no β-H atom to transfer (following toluene benzylic activation) or β-H transfer would afford a very high-energy benzyne complex. We conclude that for these substrates, the experimentally observed HIE (H/D exchange) proceeds via mechanism **B**. The relative barriers calculated by DFT are quite consistent, within the expected accuracy limits, with these results. Thus, the barrier to HIE exchange between benzene and **1-H** ^**+**^ via mechanism **B** is greater than that calculated for COA via Mechanism **A**, but less than that calculated for cyclohexane via either Mechanism **A** or **B** in accord with experimental findings (Figures 11 and 12 [Fig fig12]). Likewise, the barrier to benzene HIE via Mechanism **B** is calculated to be less than that for the benzylic C–H bond of toluene, consistent with the more rapid exchange of para- and meta-C–H bonds of toluene versus exchange at the benzylic position. Notably, however, in the case of ethylbenzene, exchange is more rapid at both benzylic and methyl group positions than at the para- and meta-positions; this is obviously attributable to the viability of Mechanism **A** for H/D exchange with ethylbenzene C­(sp^3^)–H bonds, in contrast with those of toluene.

## Summary and Conclusions

The oxidative addition of C–H bonds by transition metal complexes and the dehydrogenation of alkanes proceeding via C–H oxidative addition have been studied extensively for over 40 years. In this work, we report efficient catalytic alkane dehydrogenation using the high- oxidation-state Ir­(III) metal fragment [(^iPr^PCP)­IrH]^+^ and we give evidence for a critical role of β-agostic interactions in promoting activity. In the reaction of [(^iPr^PCP)­IrH]^+^ with an alkane H–C–C–H linkage, one C–H bond achieves complete α-C–H addition and one forms a strong β-C–H agostic bond. When the β-C–H is oriented toward the preexisting Ir–H bond, hydrogen transfer to the hydride ligand (BHHT) smoothly follows, thereby completing 1,2-dehydrogenation of the alkane to give the corresponding hydride-olefin-dihydrogen complex. This can result in hydrogen isotope exchange (HIE) with the initially present hydride ligand if the back-reaction follows, or the loss of free alkene and catalytic transfer-dehydrogenation in the presence of a sacrificial hydrogen acceptor. The energy surface of the overall process is calculated to have at most a very shallow energy minimum and the overall C–H addition and β-H transfer leading to coordinated alkene may be considered an effectively asynchronous concerted process.^95,96^ This pathway, which has no reported precedent to our knowledge, results in selectivity for both HIE and catalytic dehydrogenation that is distinct from that of established systems for either of these reactions. Specifically, H–C–C–H linkages that favor the formation of β-agostic interactions (which correlates with the energetics of formation of an eclipsed conformer) undergo particularly facile HIE and catalytic dehydrogenation. Thus, strained cycloalkanes (cyclopentane, cycloheptane, cyclooctane) undergo HIE more readily than benzene or the benzylic group of toluene, as well as HIE and catalytic dehydrogenation much more rapidly than cyclohexane or *n*-alkane. Microscopic reversibility dictates that the same factors would favor hydrogenation and indeed the rates of cycloalkene hydrogenation are found to correlate positively with strain in the alkane product, and therefore correlate *inversely* with the thermodynamic driving force. In view of the importance of hydrogenation and dehydrogenation, as well as the great interest in C–H activation and HIE, we believe that these findings open potentially valuable new approaches to the design of catalysts based on consideration of β-agostic and perhaps other secondary interactions.

## Supplementary Material





## Notes

Complete experimental details and synthetic procedures, NMR data, computational details and data, computed energies and thermodynamic quantities (PDF)
Optimized structures for calculated species (in .mol format) (ZIP)
