# Supplementary material for: Alkane Dehydrogenation and H/D Exchange by a Cationic Pincer-Ir(III) Hydride: Cooperative C–H Addition and β‑H Elimination Modes Induce Anomalous Selectivity
Source: J Am Chem Soc. 2025 Mar 13;147(12):10279–97. doi: 10.1021/jacs.4c16699 (PMC12333025; doi:10.1021/jacs.4c16699)
Supplement: Supplementary file 1 [file ja4c16699_si_001.pdf]

Supporting Information for:

Alkane Dehydrogenation and H/D Exchange by a Cationic Pincer-Ir(III) Hydride: Cooperative C-H Addition and β-H Elimination Modes Induce Anomalous Selectivity

Ashish Parihar[a], Thomas J. Emge[a], Faraj Hasanayn[b]* Alan S. Goldman[a]*
[a] *Department of Chemistry and Chemical Biology, Rutgers, The State University of New Jersey, New Brunswick, New Jersey 08903, United States*
[b] *Department of Chemistry, American University of Beirut, Beirut 1107 2020, Lebanon*

*Email: fh19@aub.edu.lb ; alan.goldman@rutgers.edu

Table of Contents

S1. General Considerations........ **S2**

S2. Synthesis

a) [(^{iPr}PCP)IrH][BArF^{24}]........ **S4**
b) [(^{iPr}PCP)IrH(CO)$_2$][BArF^{24}]........ **S4**
c) [(^{iPr}PCP)IrH(H$_2$)][BArF^{24}] **S5**
d) [(^{iPr}PCP)IrH(olefin)][BArF^{24}]........ **S6**

S3. NMR Spectra **S8**

S4. Transfer Dehydrogenation Data........ **S22**

S5. Competition Hydrogenation Data........ **S26**

S6. Competition H/D Exchange Data........ **S33**

S7. Crystallographic Data

a) [(^{iPr}PCP)IrH(CO)$_2$][BArF^{24}]........ **S39**
b) [(^{iPr}POCP^{tBu})IrH(CO)$_2$][BArF^{24}]........ **S40**
c) [(^{iPr}PCP)IrH(COE)(H$_2$O)][BArF^{24}]........ **S41**
d) [(^{iPr}PCP)IrH(C$_3$H$_6$)(H$_2$O)][BArF^{24}]........ **S42**

S8. Computational Details........ **S43**

S9. Calculated Thermodynamic Quantities........ **S44**

S10. References........ **S49**

S1. General Considerations

All reactions were conducted under an argon atmosphere, either in an MBraun glovebox or using a Schlenk line. All glassware was thoroughly cleaned and dried in an oven maintained at 140 °C for at least 8 hours. (^{iPr}PCP)IrHCl, (^{tBu}PCP)IrHCl, (^{Ad}PCP)IrHCl and (^{iPr}PCOP^{tBu})IrHCl were prepared according to previously reported methods[S1-4]. Anhydrous benzene, toluene, p-xylene, mesitylene, hexanes were purchased from Sigma Aldrich, degassed by sparging with argon, and stored over molecular sieves in a Strauss flask under an argon atmosphere and used without further purification. Propylene (99.0 %), ethylene (99.0%), H_2 (UHP), CO (UHP) were all purchased from Airgas. Deuterated solvents were obtained from Cambridge Isotope Labs, degassed by three freeze-pump-thaw cycles on the Schlenk line, dried by stirring over neutral alumina overnight and then stored over molecular sieves in the glovebox. NMR spectra were acquired on 500-MHz Varian and 500-MHz Bruker Ascend NMR spectrometers. ^{1}H and ^{13}C NMR spectra were referenced to residual solvent peaks. Gas Chromatographic analyses (FID detection) were performed on a Varian 430– GC instrument equipped with Agilent J&W GS-GasPro column (60 m length x 0.32 mm ID) using the following method:

Varian 430–GC Detector:

FID starting temperature: 40 °C

Time at starting temp: 1.4 min

Ramp1: 20 °C/min up to 200 °C withhold time = 3 min Ramp2: 30 °C/min up to 260 °C withhold time = 70.6 min

Flow rate (carrier): 1.4 mL/min (N_2)

Split ratio: 25

Inlet temperature: 250 °C

Detector temperature: 260 °C

S1.a. General procedure for transfer dehydrogenation of alkane using an alkene hydrogen acceptor:

In an argon-filled glovebox, a screw-cap Schlenk flask (ca. 2.0 mL) with a magnetic bead was charged with catalyst (4.8 mM) and Na[BArF^{24}], followed by addition of 1.0 mL of the desired alkane substrate and acceptor alkene along with mesitylene (100 mM) as in internal standard. After heating an oil bath to the desired temperature and allowing it to stabilize, the sealed Schlenk flask was taken out of the glovebox, attached to an argon Schlenk line, and fully immersed in the oil bath. To record data, the reaction flask was removed from the oil bath, cooled to 0 °C with an ice bath, and the screw cap was carefully removed under a strong argon flow. An aliquot (0.7 µL) of the reaction mixture was collected using a 1-microliter syringe and directly injected into the GC for analysis. The turnover number was determined by integration of the dehydrogenated product, using the concentrations of the internal standard mesitylene and the catalyst:

$$TON = [[\text{Mesitylene standard}]/(\text{Mesitylene}_{integral})] \times [(\text{Dehydrogenated Product}_{integral})]/[\text{Catalyst}]]$$

S1.b. General procedure for alkane-dehydrogenation competition experiments:

In an argon-filled glovebox, a screw-cap Schlenk flask (ca. 1.0 mL or 2.0 mL) containing a magnetic stirring bead was charged with the required amount of catalyst and 1 equivalent of Na[BArF^{24}], followed by the addition of 0.25mL of each alkane substrate and 2.4 M acceptor alkene, along with mesitylene (100 mM) as an internal standard. An oil bath was heated to the desired temperature and stabilized and the sealed Schlenk flask was removed from the glovebox, connected to an argon Schlenk line, and fully immersed in the oil bath. For data collection, the reaction flask was taken out of the oil bath, cooled to 0 °C using an ice bath, and the screw cap was carefully removed under a strong argon flow. An aliquot (0.7 mL) of the sample was collected using a 1-microliter syringe and directly injected into the GC for analysis. The turnover number was determined by integrating the dehydrogenated product, using the concentrations of the internal standard mesitylene and the catalyst.

S1.c. General procedure for alkene-hydrogenation competition experiments:

In a J-Young NMR tube, the required amount of catalyst and 1 equiv Na[BArF^{24}] were added, followed by 0.4 mL of benzene-d_6. The reaction mixture was heated to 80 °C for 5 minutes to activate the catalyst. 0.1 mL of the respective alkene was then added. ^{1}H NMR spectroscopy was used to determine the concentration of hydrogenation product, with the residual benzene signal in the solvent serving as an internal standard. The zero-point data was taken before adding H_2, after which 1.9 atm of H_2 was added. To ensure mixing of the gas in the solution, the NMR tube was rotated before recording each ^{1}H NMR spectrum to quantify the hydrogenation product. The concentration of hydrogenated product was calculated using the initial and final concentrations of alkene and the residual benzene signal.

S1.d. General procedure for competitive H/D exchange catalysis:

In a J-Young NMR tube, the required amount of catalyst and 1 equiv Na[BArF^{24}] were added, followed by 0.3 mL of benzene-d_6 and 0.2 mL of the deuterated competitive partner substrate. The reaction mixture was then heated to 80 °C for 5 minutes to activate the catalyst, after which the H-source (either H_2 or dioxane) was added. Mesitylene was used as an external standard, with its aryl-H peaks serving as a reference. H/D exchange was monitored by ^{1}H NMR spectroscopy, observing the increase in the residual solvent peak. The zero-point data (which is the residual solvent peak of substrates); were collected before heating and introducing H_2 for efficient mixing of the gas in the solution, the tube was simultaneously heated and rotated.

For the intramolecular H/D exchange, a J-Young NMR tube was loaded with the required quantity of catalyst and 1 equiv Na[BArF^{24}], followed by the addition of 0.4 mL of the deuterated substrate. The reaction mixture was heated to 80 °C for 5 minutes to activate the catalyst, and then 0.1 mL of COA was added as the H-source. The zero-point data was collected before heating the reaction mixture, and the increase in the residual peak was used to determine the concentration of H/D exchange product.

S2. Synthesis

S2.a. Synthesis of [(^{iPr}PCP)IrH][BArF^{24}]

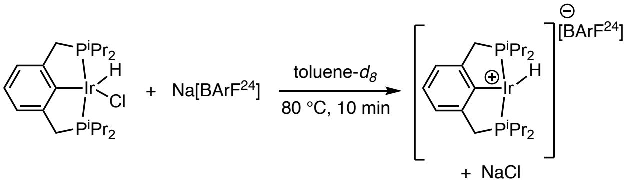

A J-Young NMR tube was loaded with Na[BArF^{24}], (7.8 mg, 0.0088 mmol) and (^{iPr}PCP)IrHCl (5 mg, 0.0088 mmol) inside an argon-filled glovebox, followed by the addition of toluene-d$_8$ (0.5 mL), resulting in a dark, orange-colored solution. The tube was then rotated for about 15 minutes at room temperature to ensure thorough mixing of Na[BArF^{24}] in solution. The reaction mixture was then heated to 80 °C for an additional 10 minutes. The color change to yellowish-orange indicated the formation of [(^{iPr}PCP)IrH$^+$]. The ^{31}P{^{1}H} NMR spectrum showed a broad signal along with some small sharp peaks (Figure S4), possibly due to reversible binding of a solvent molecule, which caused broadening. The ^{1}H NMR spectrum showed no sign of a metal hydride signal.

The NMR probe was then heated to 100 °C, and the spectra were recorded. The ^{31}P{^{1}H} NMR spectrum displayed a sharp single signal at δ 59.67, indicating the formation of [(^{iPr}PCP)IrH$^+$]. The ^{1}H NMR spectrum also revealed a metal hydride signal at δ -41.59, indicating a hydride trans to a vacant coordination site.

Characterization:

^{1}H NMR at 298K (500 MHz, toluene-*d*$_8$) δ 8.27 (d, *J* = 4.7 Hz, 8H) (BArF^{24} H), 7.67 (s, 4H) (BArF^{24} H), 6.74 (s, 3H), 2.66 (s, 4H), 2.16 (d, *J* = 13.4 Hz, 2H), 1.80 (s, 2H), 0.81 (dt, *J* = 20.2, 7.4 Hz, 24H). **^{31}P{^{1}H} NMR at 298K (202 MHz, toluene-*d*$_8$)** δ 55.03 (d, *J* = 320.3 Hz). **^{13}C NMR at 298K (126 MHz, toluene-*d*$_8$)** δ 161.63 (dd, *J* = 99.7, 49.8 Hz), 148.32 136.46, 134.50, 128.85 (td, *J* = 33.1, 25.8 Hz), 128.15, 127.22, 125.36, 124.38, 123.19, 121.60, 118.26 – 115.57 (m), 31.27, 25.56, 23.44 (t, *J* = 16.1 Hz), 21.77, 17.53 (d, *J* = 23.5 Hz), 17.15, 16.67. **^{19}F NMR at 298K (471 MHz, toluene-*d*$_8$)** δ -62.13. **^{11}B NMR at 298K (160 MHz, toluene-*d*$_8$)** δ -6.00. **^{1}H NMR at 373K (500 MHz, toluene-*d*$_8$)** δ 8.19 (s, 8H)) (BArF^{24} H), 7.70 (s, 4H)) (BArF^{24} H), 6.81 (s, 2H), 6.76 (d, *J* = 7.4 Hz, 1H), 2.87 (s, 4H), 2.37 (s, 2H), 2.00 (s, 2H), 0.92 (dd, *J* = 16.7, 8.3 Hz, 18H), 0.88 – 0.78 (m, 6H), -41.59 (s, 1H) (Ir-H). **^{31}P{^{1}H} NMR at 373K (202 MHz, toluene-*d*$_8$)** δ 59.67.

S2.b. Synthesis of [(^{iPr}PCP)IrH(CO)$_2$][BArF^{24}]:

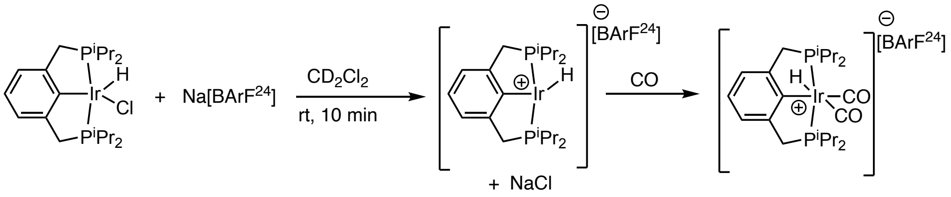

A J-Young NMR tube was charged with Na[BArF^{24}] (7.8 mg, 0.0088 mmol) and (^{iPr}PCP)IrHCl (5 mg, 0.0088 mmol) inside the argon filled glovebox followed by the addition of the solvent (dichloromethane) on Schlenk line resulting in the formation of a dark, orange-colored solution. The reaction tube was then rotated ensuring the mixing of Na[BArF^{24}] in CD$_2$Cl$_2$ for about 15 minutes at room temperature. The change of solution color to yellowish orange

ensured the formation of (^{iPr}PCP)IrH$^+$, possibly binding a solvent molecule The reaction mixture was then charged with 1 atm of CO and allowed the mixing of gas into the solution by rotation the tube for another 15 min at room temperature. This resulted in a change of color to a very light-yellowish solution. The solvent and excess CO was then removed in vacuo resulting in the formation of off white solid which on addition of the $CDCl_3$ yielded the product **[(^{iPr}PCP)IrH(CO)$_2$][BArF^{24}]** as evidenced by various NMR spectroscopic techniques in 87 % NMR Yield.(possible chlorination of aryl ring in byproduct, so dichloromethane and $CDCl_3$ was not used for solvent after this).

Characterization:

^{1}H NMR at 298K (500 MHz, chloroform-*d*) δ 7.54 (d, *J* = 4.7 Hz, 8H) (BArF^{24} H), 7.36 (s, 4H) (BArF^{24} H),, 6.94 (d, *J* = 7.6 Hz, 2H), 6.91 – 6.85 (m, 1H), 3.41 (qt, *J* = 17.2, 4.3 Hz, 4H), 2.34 – 2.24 (m, 2H), 2.15 (td, *J* = 14.7, 13.3, 8.4 Hz, 2H), 1.14 (dq, *J* = 16.9, 7.6 Hz, 12H), 0.98 (dt, *J* = 12.3, 7.0 Hz, 6H), 0.80 (q, *J* = 7.5 Hz, 6H), -10.42 (t, *J* = 12.8 Hz, 1H). **^{31}P{^{1}H} NMR at 298K (202 MHz, chloroform-*d*)** δ 51.11. **^{13}C NMR at 298K (126 MHz, chloroform-*d*)** δ 166.36 (Ir-CO), 165.20 (Ir-CO), 161.67 (dd, *J* = 99.6, 49.9 Hz), 146.14 (t, *J* = 6.1 Hz), 134.79, 133.88, 129.68 – 128.18 (m), 127.72 (d, *J* = 18.2 Hz), 125.63, 123.79 (t, *J* = 8.2 Hz), 123.46, 121.30, 117.65 – 117.18 (m), 38.44 (t, *J* = 17.7 Hz), 27.02 (t, *J* = 14.9 Hz), 26.07 (t, *J* = 17.3 Hz), 19.84, 18.94 (d, *J* = 5.3 Hz), 18.10, 17.67. **^{19}F NMR at 298K (471 MHz, chloroform-*d*)** δ -62.38. **^{11}B NMR at 298K (160 MHz, chloroform-*d*)** δ -6.62. (s).

S2.c. Synthesis of [(^{iPr}PCP)IrH(H$_2$)][BArF^{24}]

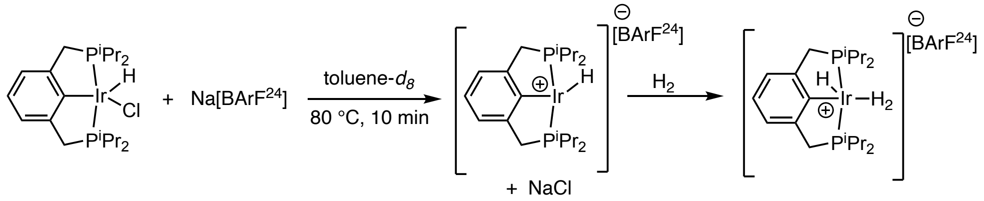

A J-Young NMR tube was charged with Na[BArF^{24}] (7.8 mg, 0.0088 mmol) and (^{iPr}PCP)IrHCl (5 mg, 0.0088 mmol) inside an argon-filled glovebox, followed by the addition of toluene-d$_8$ (0.5 mL), resulting in a dark orange solution. The tube was then rotated to ensure thorough mixing, for approximately 15 minutes at room temperature, after which the mixture was heated to 80 °C for an additional 10 minutes. The NMR tube was then charged with 1 atm H_2 gas and the solution color changed to pale yellow.

The ^{31}P{^{1}H} NMR spectrum revealed two signals at δ 54.9 and 57.1 ppm in a 70:30 ratio. The ^{1}H NMR spectrum displayed two signals in the upfield region at δ -4.0 and -26.8 in a 2:1 ratio, consistent with the formation of [(^{iPr}PCP)IrH(H$_2$)][BArF^{24}]. [^{31}P]-[^{1}H] HMBC studies correlated the hydride peaks with the major fragment observed in the ^{31}P{^{1}H} NMR spectrum. VT-NMR studies provided further evidence for the formation of [(^{iPr}PCP)IrH(H$_2$)][BArF^{24}]. It was found that [(^{iPr}PCP)IrH(H$_2$)][BArF^{24}] is stable only under H_2 atmosphere; removal of H_2 and subsequent exposure to vacuum led to the formation of [(^{iPr}PCP)IrH][BArF^{24}].

Characterization:

^{1}H NMR (500 MHz, toluene-*d*$_8$) at 298K : δ 8.30 (d, *J* = 5.2 Hz, 8H), 7.70 (s, 4H), 6.90 – 6.71 (m, 3H), 2.81 (dt, *J* = 16.8, 4.6 Hz, 2H), 2.60 (d, *J* = 29.9 Hz, 2H), 1.57 – 1.48 (m, 2H), 1.48 – 1.40 (m, 2H), 0.97 – 0.89 (m, 6H), 0.71 – 0.55 (m, 12H), 0.43 (q, *J* = 7.3 Hz, 6H), -4.00 (s, 2H), -26.89 (d, *J* = 12.5 Hz, 1H). **^{31}P NMR (202 MHz, toluene-*d*$_8$):** δ 54.93

^{13}C NMR (126 MHz, toluene-d_8): δ 163.31 – 161.03 (m), 147.81, 135.15, 129.63, 129.34 (d, J = 9.0 Hz), 127.99, 127.13 (d, J = 8.3 Hz), 126.01, 123.84, 122.93, 121.73 (d, J = 12.9 Hz), 117.61 (d, J = 6.0 Hz), 34.15, 25.82, 24.74 (t, J = 15.8 Hz), 22.42, 18.14, 17.66, 16.48. **^{19}F NMR (471 MHz, toluene-d_8)**: δ -62.11. **^{11}B NMR (160 MHz, toluene-d_8)**: δ -6.01 (d, J = 2.8 Hz).

S2.d. Synthesis of [(^{iPr}PCP)IrH(olefin)][BArF^{24}]:

Reaction of [(^{iPr}PCP)IrH][BArF^{24}] with C_2H_4:

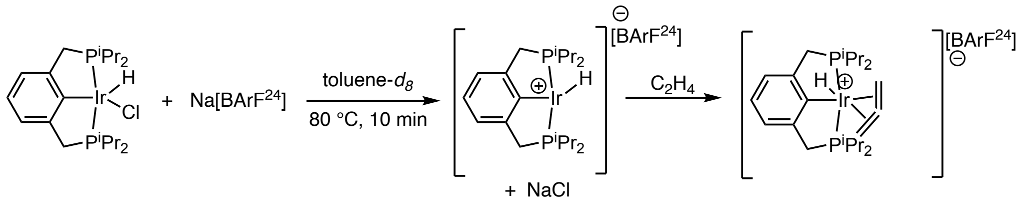

In a J-Young NMR tube, [(^{iPr}PCP)IrH][BArF^{24}] was synthesized following the procedure described above. Upon charging the toluene solution of [(^{iPr}PCP)IrH][BArF^{24}] with 1 atm C_2H_4, the solution color changed from yellow-orange to very light yellow. Characterization by ^{31}P{^{1}H} NMR spectroscopy at 298 K showed a very broad signal at 29.6 ppm. The ^{1}H NMR spectrum at 298 K displayed no metal-hydride peaks, while the signals corresponding to free ethylene appeared as broad peaks at 5.1 ppm (compared to 5.25 ppm in the absence of the metal complex).

At a lower temperature, 233 K, the ^{31}P{^{1}H} NMR spectrum showed a sharp peak at 27.18 ppm, and the ^{1}H NMR spectrum displayed a peak in the upfield region at -13.3 ppm, corresponding to the metal hydride. The signals for free ethylene became relatively sharper at 5.24 ppm at this temperature. When the ethylene pressure was increased from 1 atm to 1.6 atm, the ^{31}P{^{1}H} NMR signals at 298 K became sharper compared to those at 1 atm and shifted to 28.6 ppm.

These observations suggest a rapid exchange between coordinated and free ethylene and indicate the existence of an equilibrium between the mono- and bis-ethylene complexes, [(^{iPr}PCP)IrH(C_2H_4)][BArF^{24}] and [(^{iPr}PCP)IrH(C_2H_4)$_2$][BArF^{24}]. The relevant NMR spectra are provided in the NMR Spectra section below.

Reaction of [(^{iPr}PCP)IrH][BArF^{24}] with propylene:

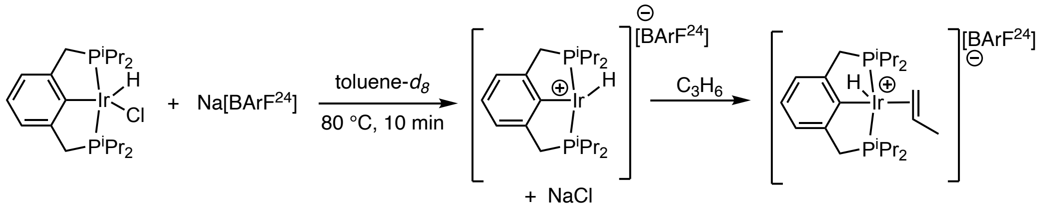

In a J-Young NMR tube, [(^{iPr}PCP)IrH][BArF^{24}] was synthesized according to the procedure described above. Upon charging the toluene solution of [(^{iPr}PCP)IrH][BArF^{24}] with 1 atm C_3H_6, the solution color changed to light yellow. ^{31}P{^{1}H} NMR spectroscopy at 298 K showed a relatively sharp signal at 46.3 ppm, sharper than the signal observed when ethylene was introduced to [(^{iPr}PCP)IrH][BArF^{24}]. In the ^{1}H NMR spectrum, a sharper signal for free propylene was noted, along with a signal in the upfield region at -34.2 ppm, corresponding to a metal hydride with trans to a vacant coordination site. At 233 K, further characterization of the complex was hindered by its low solubility in

toluene. When the temperature was raised to 323 K, the $^{31}P\{^1H\}$ NMR spectrum showed a broader peak than at 298 K. The relevant NMR spectra are provided in the NMR Spectra section below.

Reaction of [(^{iPr}PCP)IrH][BArF^{24}] with TBE:

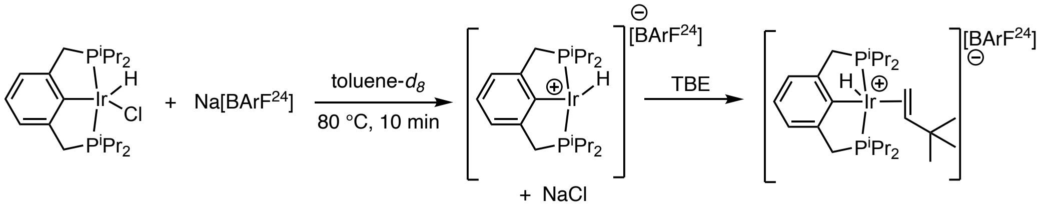

In a J-Young NMR tube, [(^{iPr}PCP)IrH][BArF^{24}] was synthesized following the previously described procedure. Upon the addition of 3 equivalents of TBE to the toluene solution of [(^{iPr}PCP)IrH][BArF^{24}], the solution turned light yellow. Characterization by $^{31}P\{^1H\}$ NMR spectroscopy at 298 K revealed a broad signal at 51.2 ppm. The ^1H NMR spectrum at 298 K displayed a broad signal for free TBE and an upfield signal at -30.7 ppm, corresponding to a metal hydride trans to vacant coordination site. At 273 K, the $^{31}P\{^1H\}$ NMR spectrum showed an even broader peak, shifted to 49.6 ppm. The ^1H NMR spectrum at 273 K showed sharp signals for free TBE, with a broad metal hydride peak at -29.9 ppm. Further cooling to 233 K led to continued broadening of the $^{31}P\{^1H\}$ NMR signals, while the ^1H NMR spectrum displayed sharp peaks for free TBE and a metal hydride signal in the same region at -29.4 ppm. The relevant NMR spectra are provided in the NMR Spectra section below.

Reaction of [(^{iPr}PCP)IrH][BArF^{24}] with cyclopentene (CPE):

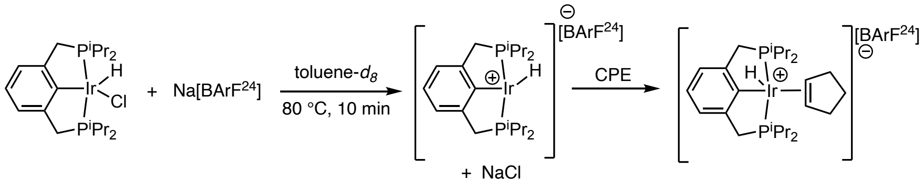

In a J-Young NMR tube, [(^{iPr}PCP)IrH][BArF^{24}] was synthesized following the procedure described above. Upon the addition of 3 equiv CPE to the toluene solution of [(^{iPr}PCP)IrH][BArF^{24}], the solution turned light yellow. Characterization by $^{31}P\{^1H\}$ NMR spectroscopy at 298 K revealed a sharp signal at 38.8 ppm, which was sharper than the signals observed when propylene or TBE was added to [(^{iPr}PCP)IrH][BArF^{24}]. The ^1H NMR spectrum showed a slightly broadened signal for free CPE and an upfield signal at -33.4 ppm, corresponding to a metal hydride trans to a vacant coordination site. When the temperature was lowered to 253 K, the $^{31}P\{^1H\}$ NMR spectrum showed very sharp signal at 38.0 ppm, while the ^1H NMR spectrum displayed sharp signals for free CPE, with a very sharp metal hydride peak at -32.8 ppm. These findings suggest the formation of a mono-olefin complex [(^{iPr}PCP)IrH(CPE)][BArF^{24}]. The relevant NMR spectra are provided in the NMR Spectra section below.

S3. NMR Spectra:

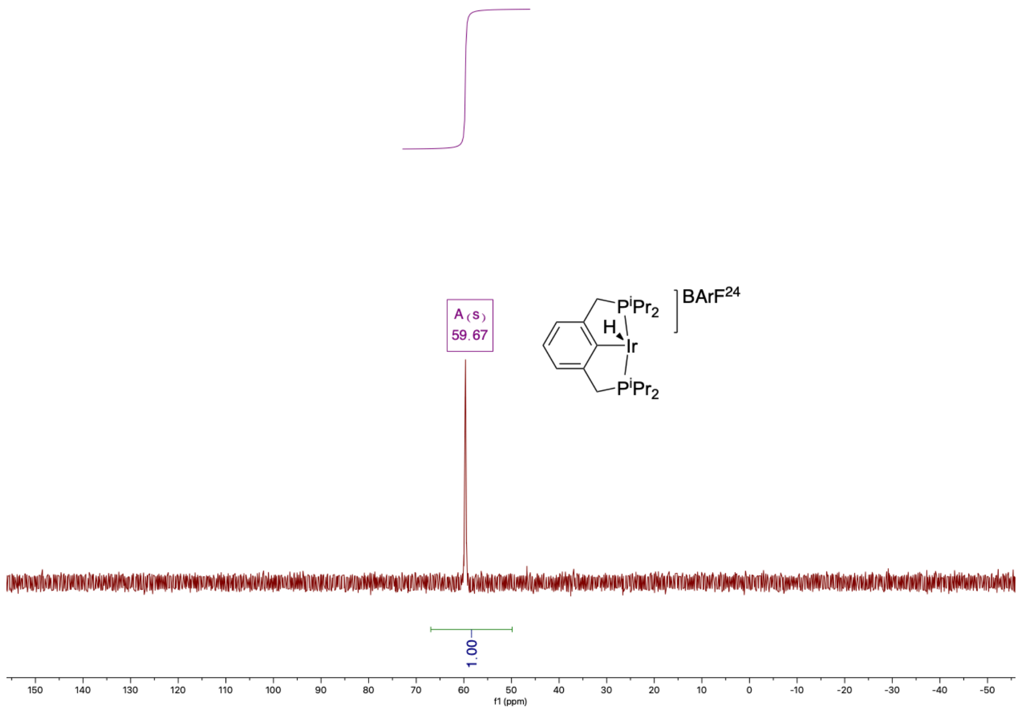

Figure S1. $^{31}P\{^1H\}$ NMR Spectrum of [(^{iPr}PCP)IrH][BArF^{24}] in toluene-d$_8$ at 373 K

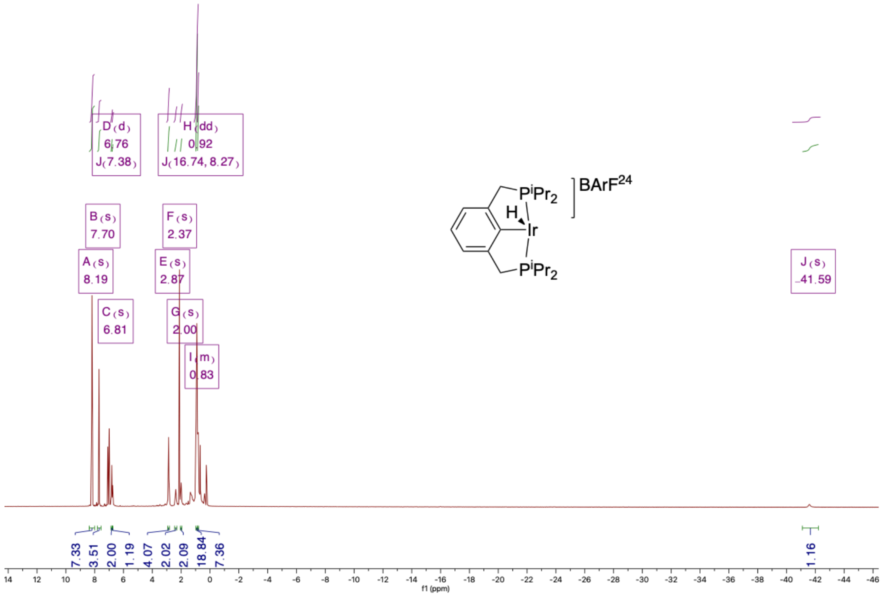

Figure S2. ^1H NMR Spectrum of [(^{iPr}PCP)IrH][BArF^{24}] in toluene-d$_8$ at 373 K

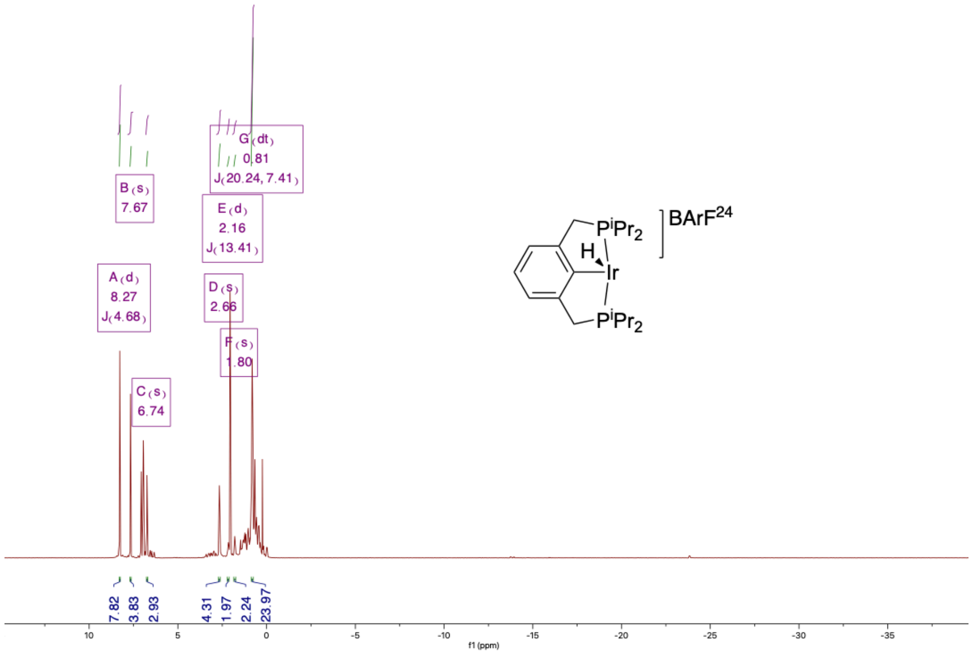

Figure S3. ^{1}H NMR Spectrum of [(^{iPr}PCP)IrH][BArF^{24}] in toluene-d_8 at 298 K

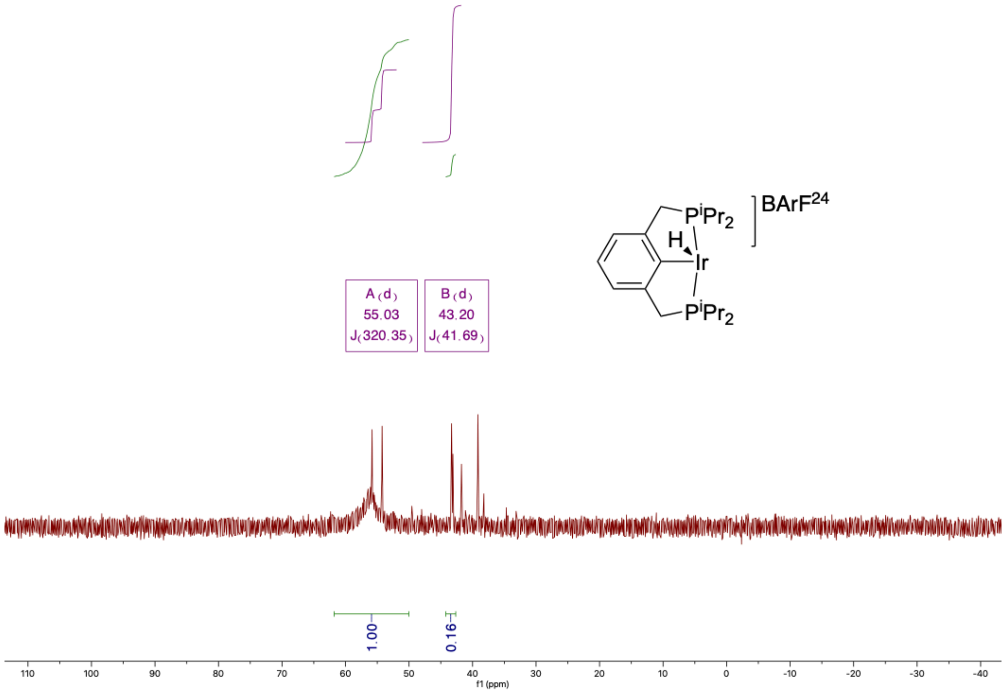

Figure S4. $^{31}P\{^{1}H\}$ NMR Spectrum of [(^{iPr}PCP)IrH][BArF^{24}] in toluene-d_8 at 298 K

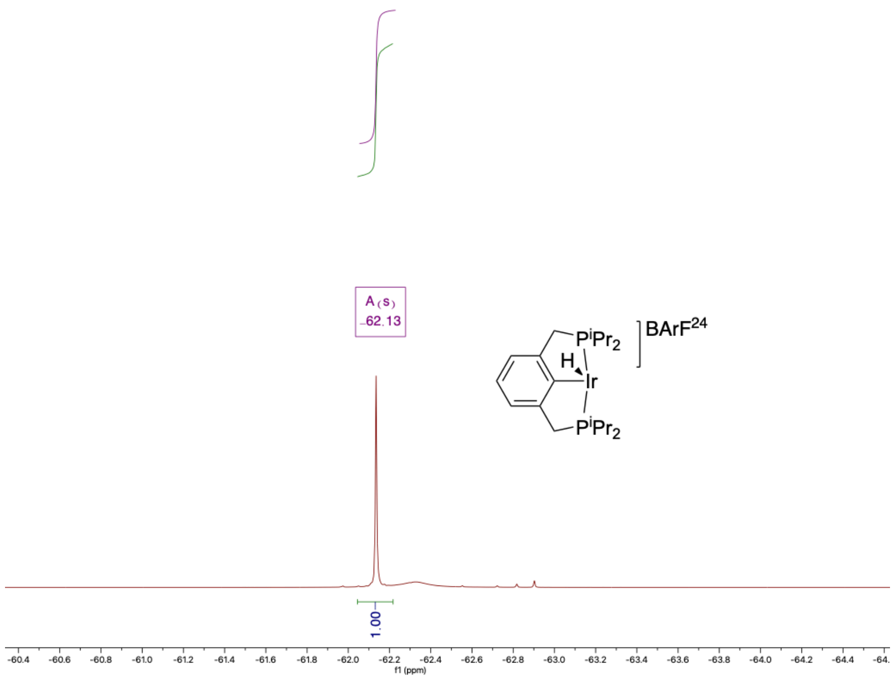

Figure S5. $^{19}F\{^{1}H\}$ NMR Spectrum of [(^{iPr}PCP)IrH][BArF^{24}] in toluene-d_8 at 298 K

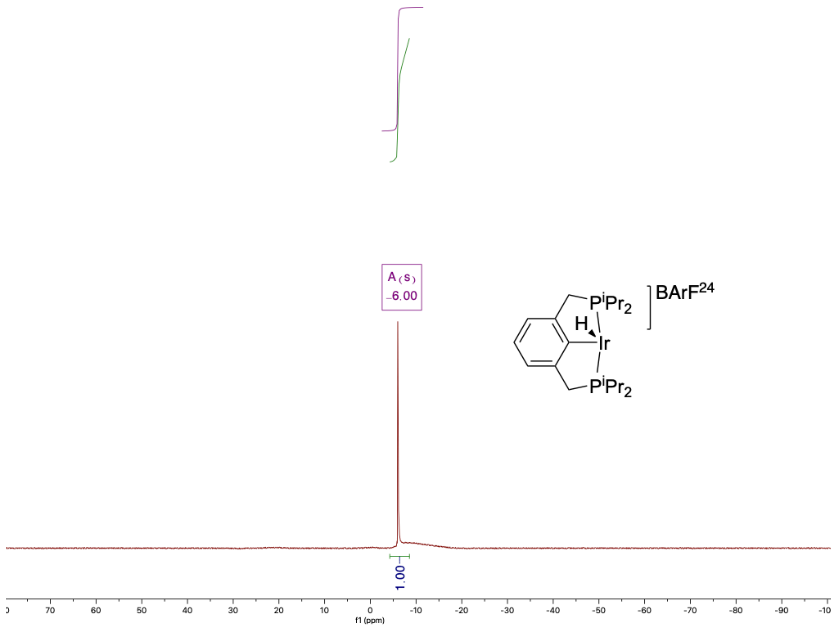

Figure S6. $^{11}B\{^{1}H\}$ NMR Spectrum of [(^{iPr}PCP)IrH][BArF^{24}] in toluene-d_8 at 298 K

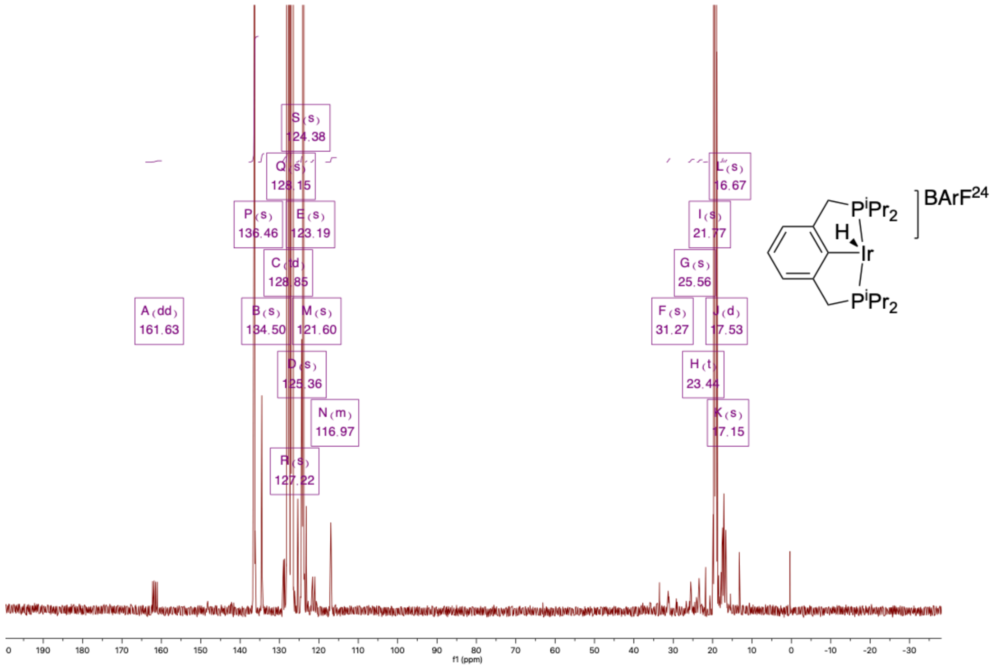

Figure S7. ^{13}C NMR Spectrum of [(^{iPr}PCP)IrH][BArF^{24}] in toluene-d$_8$ at 298 K

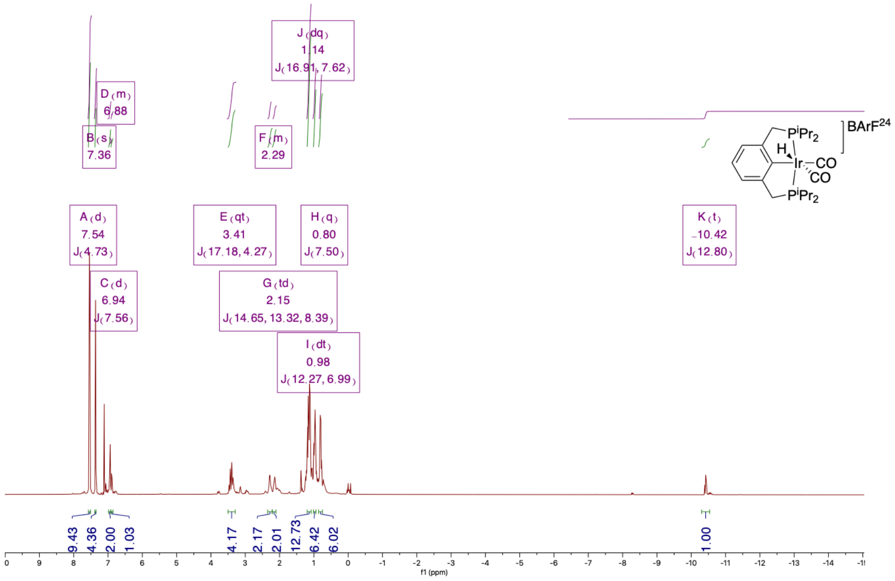

Figure S8. ^{1}H NMR Spectrum of [(^{iPr}PCP)IrH(CO)$_2$][BArF^{24}] in CDCl$_3$ at 298 K

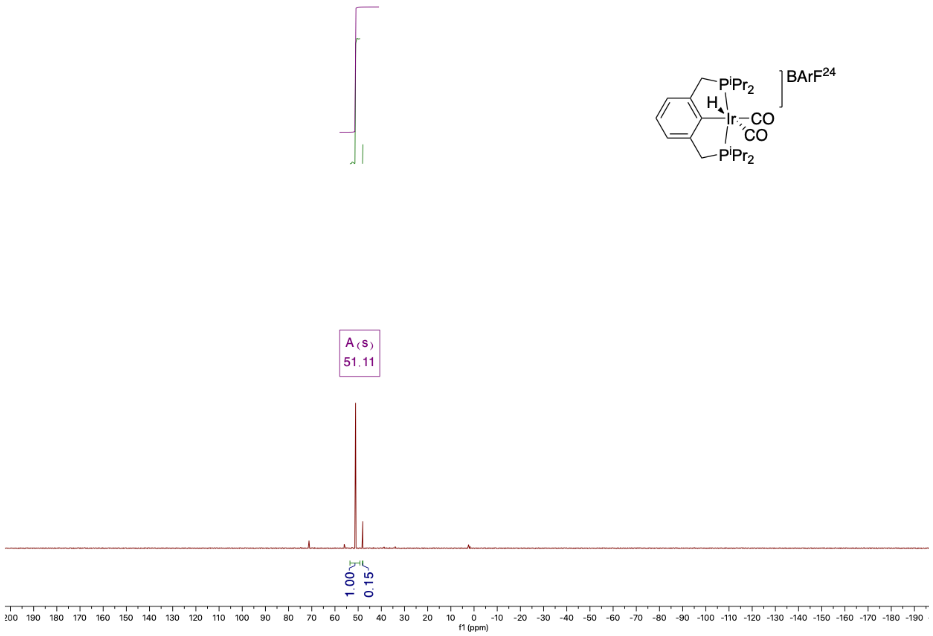

Figure S9. $^{31}P\{^{1}H\}$ NMR Spectrum of [(^{iPr}PCP)IrH(CO)$_2$][BArF^{24}] in $CDCl_3$ at 298 K

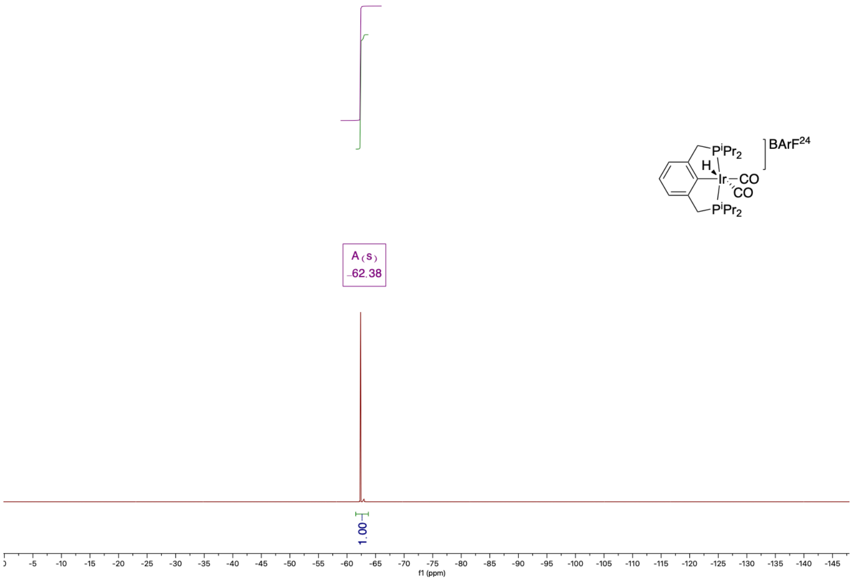

Figure S10. $^{19}F\{^{1}H\}$ NMR Spectrum of [(^{iPr}PCP)IrH(CO)$_2$][BArF^{24}] in $CDCl_3$ at 298 K

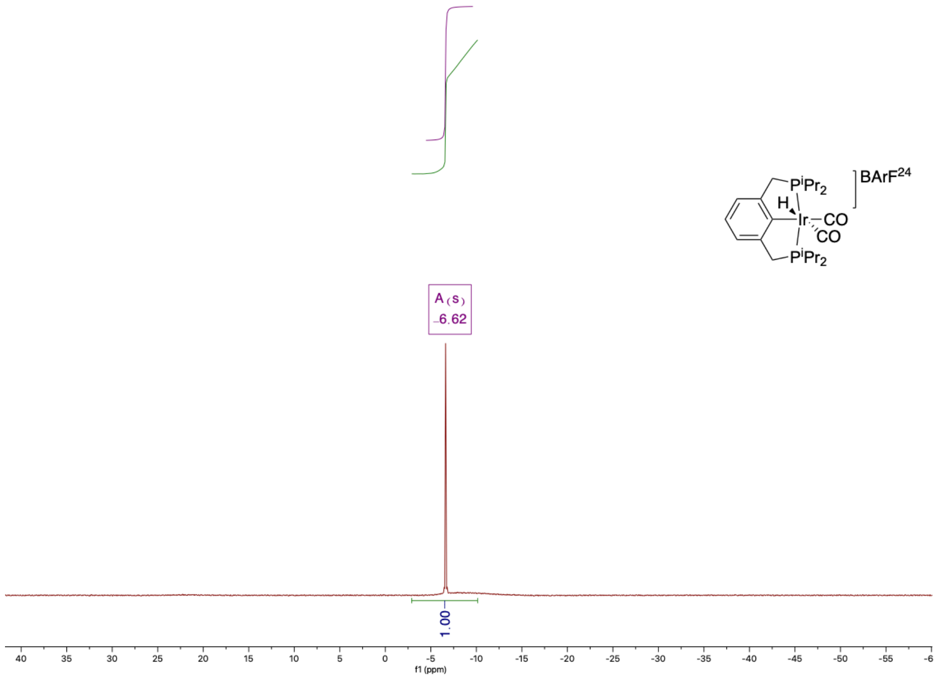

Figure S11. $^{11}B\{^{1}H\}$ NMR Spectrum of [(^{iPr}PCP)IrH(CO)$_2$][BArF^{24}] in $CDCl_3$ at 298 K

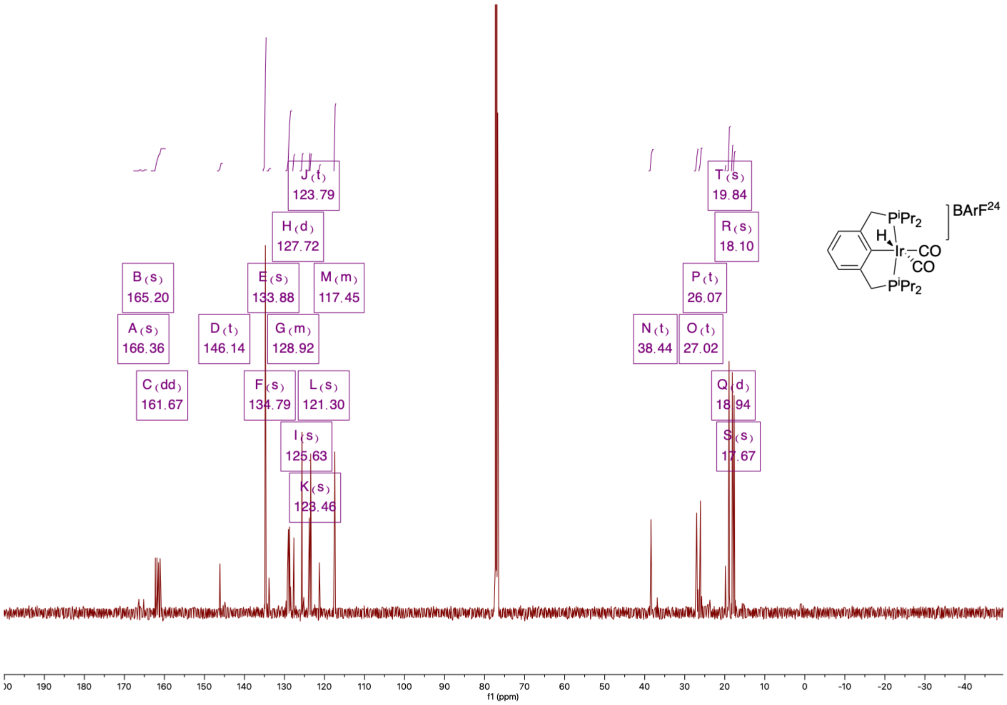

Figure S12. ^{13}C NMR Spectrum of [(^{iPr}PCP)IrH(CO)$_2$][BArF^{24}] in $CDCl_3$ at 298 K

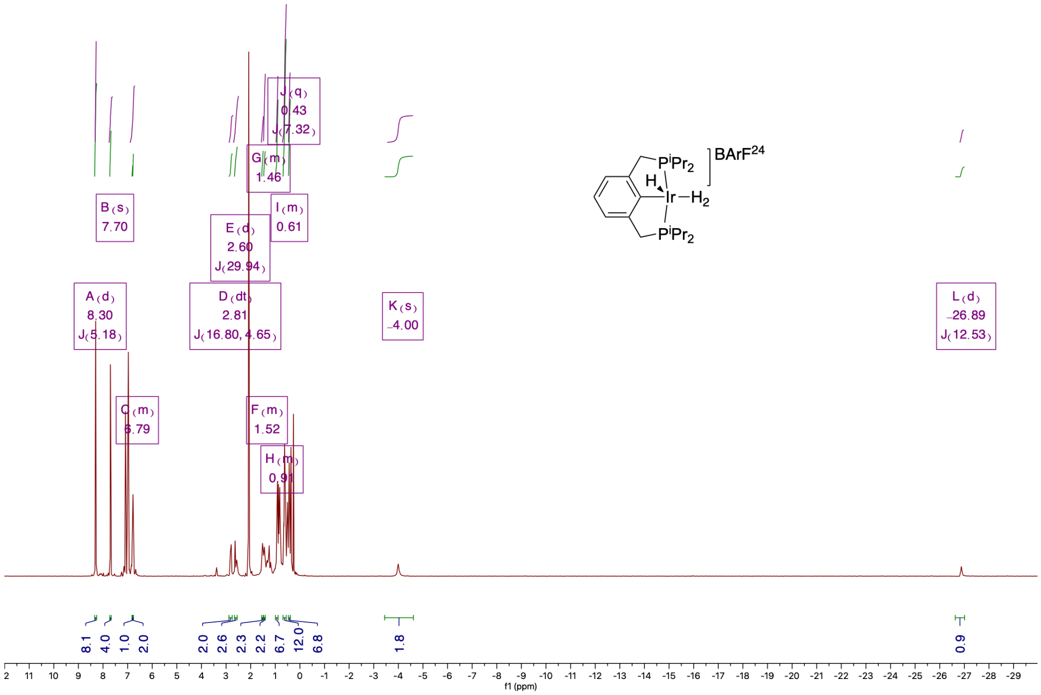

Figure S13. ^{1}H NMR Spectrum of [(^{iPr}PCP)IrH(H_2)][BArF^{24}] in toluene-d_8 at 298 K

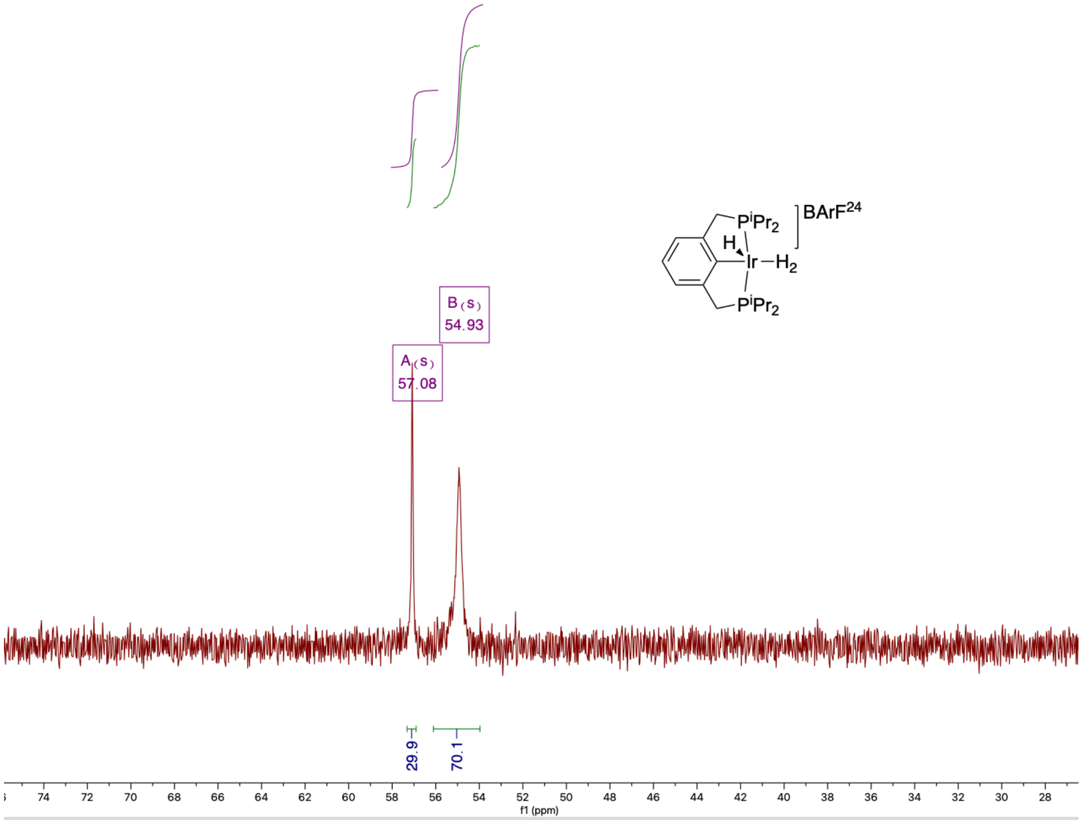

Figure S14. $^{31}P\{^{1}H\}$ NMR Spectrum of [(^{iPr}PCP)IrH(H_2)][BArF^{24}] in toluene-d_8 at 298 K

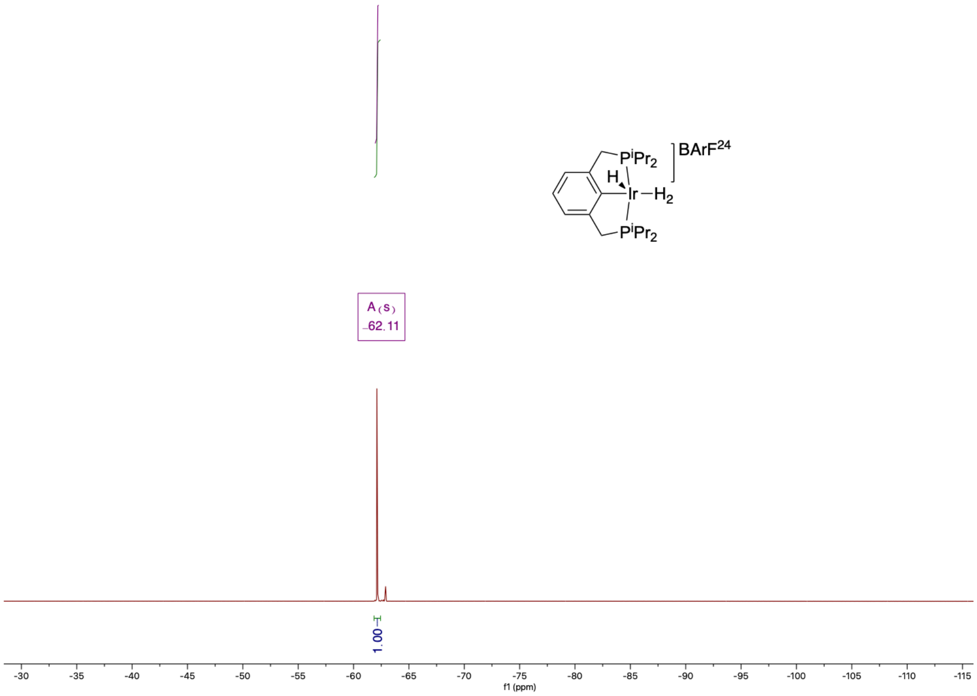

Figure S15. $^{19}F\{^{1}H\}$ NMR Spectrum of [(^{iPr}PCP)IrH(H_2)][BArF^{24}] in toluene-d_8 at 298 K

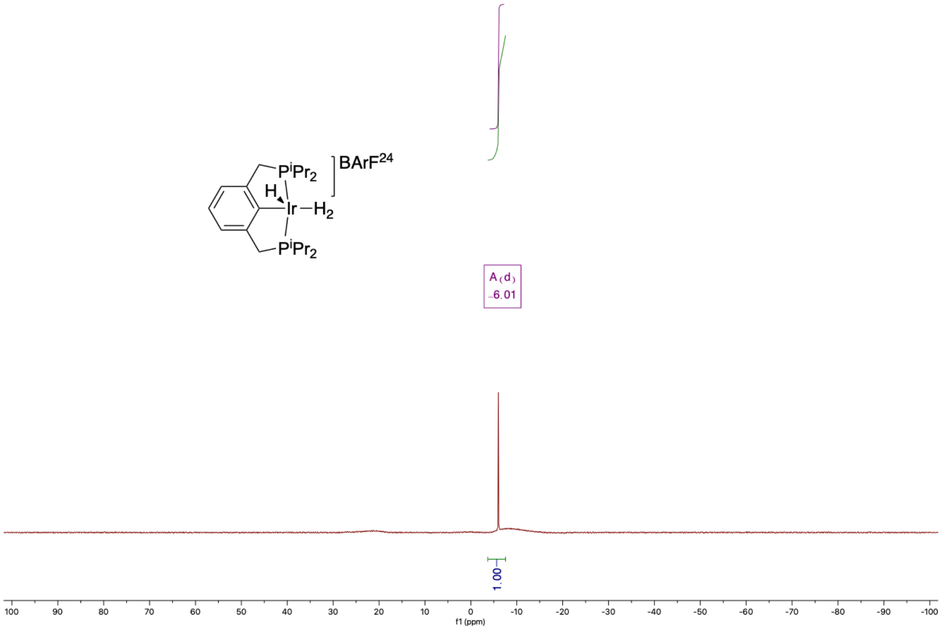

Figure S16. $^{11}B\{^{1}H\}$ NMR Spectrum of [(^{iPr}PCP)IrH(H_2)][BArF^{24}] in toluene-d_8 at 298 K

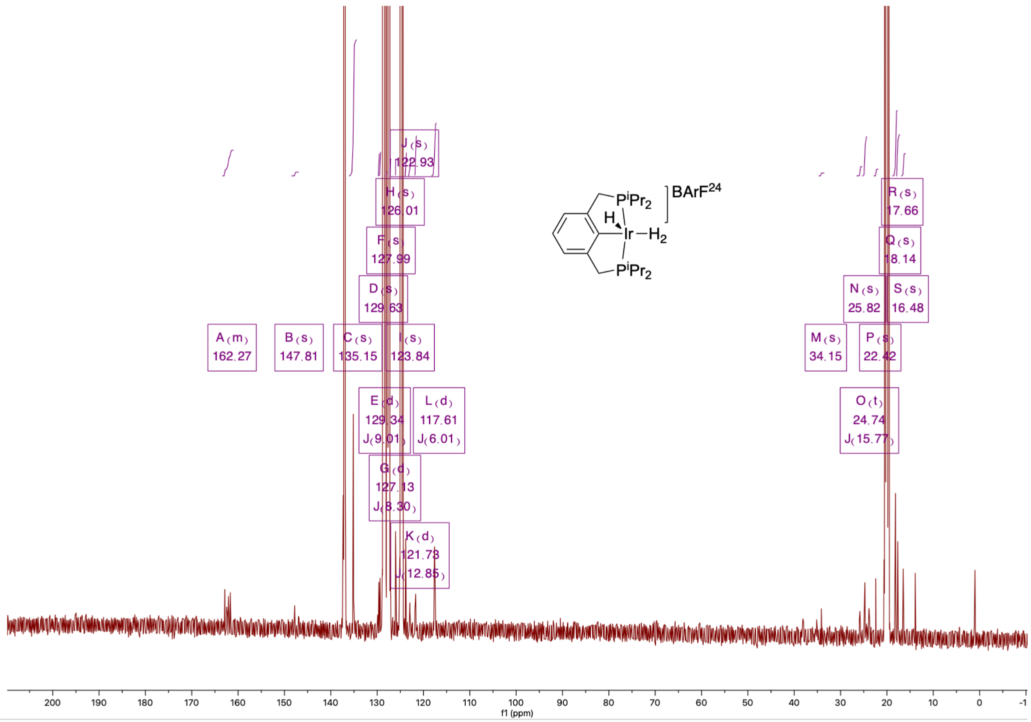

Figure S17. ^{13}C NMR Spectrum of [(^{iPr}PCP)IrH(H_2)][BArF^{24}] in toluene-d_8 at 298 K

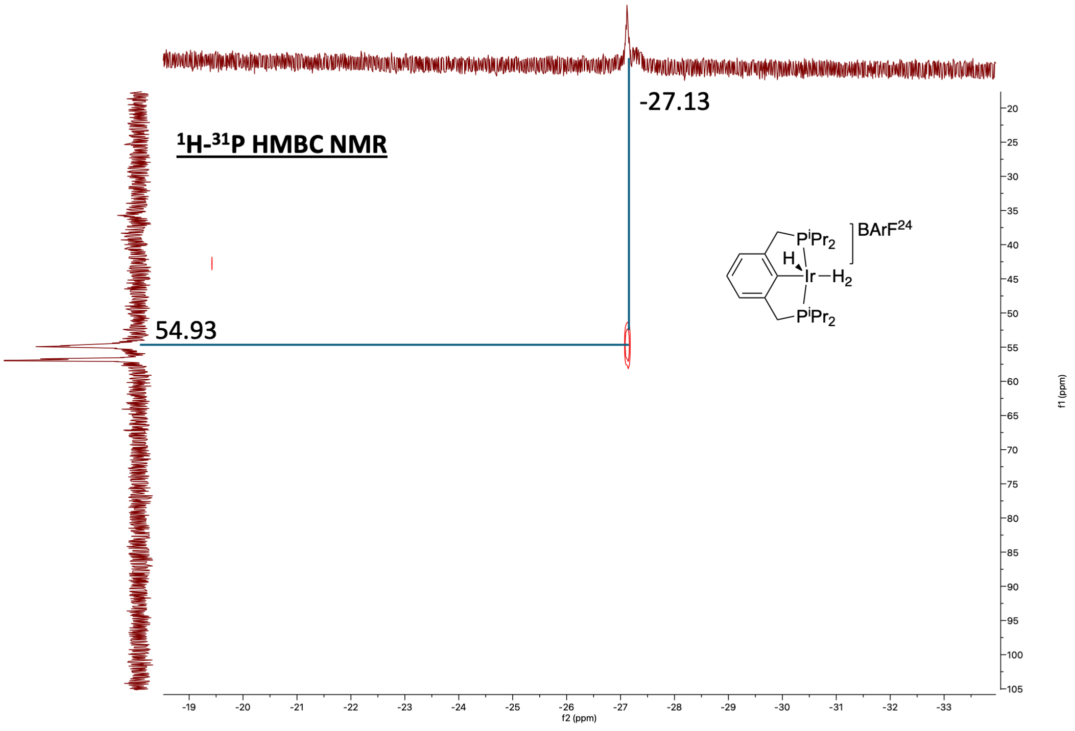

Figure S18. ^{1}H-^{31}P HMBC NMR Spectrum of [(^{iPr}PCP)IrH(H_2)][BArF^{24}] in benzene-d_6 at 298K

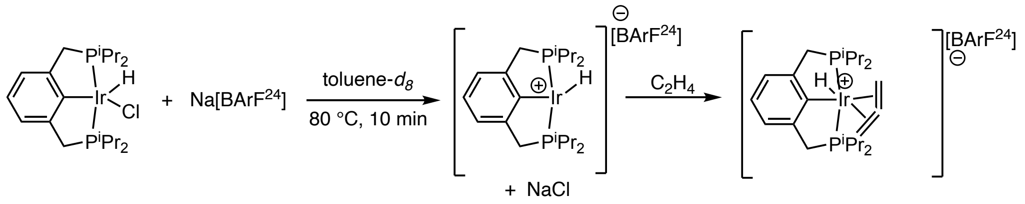

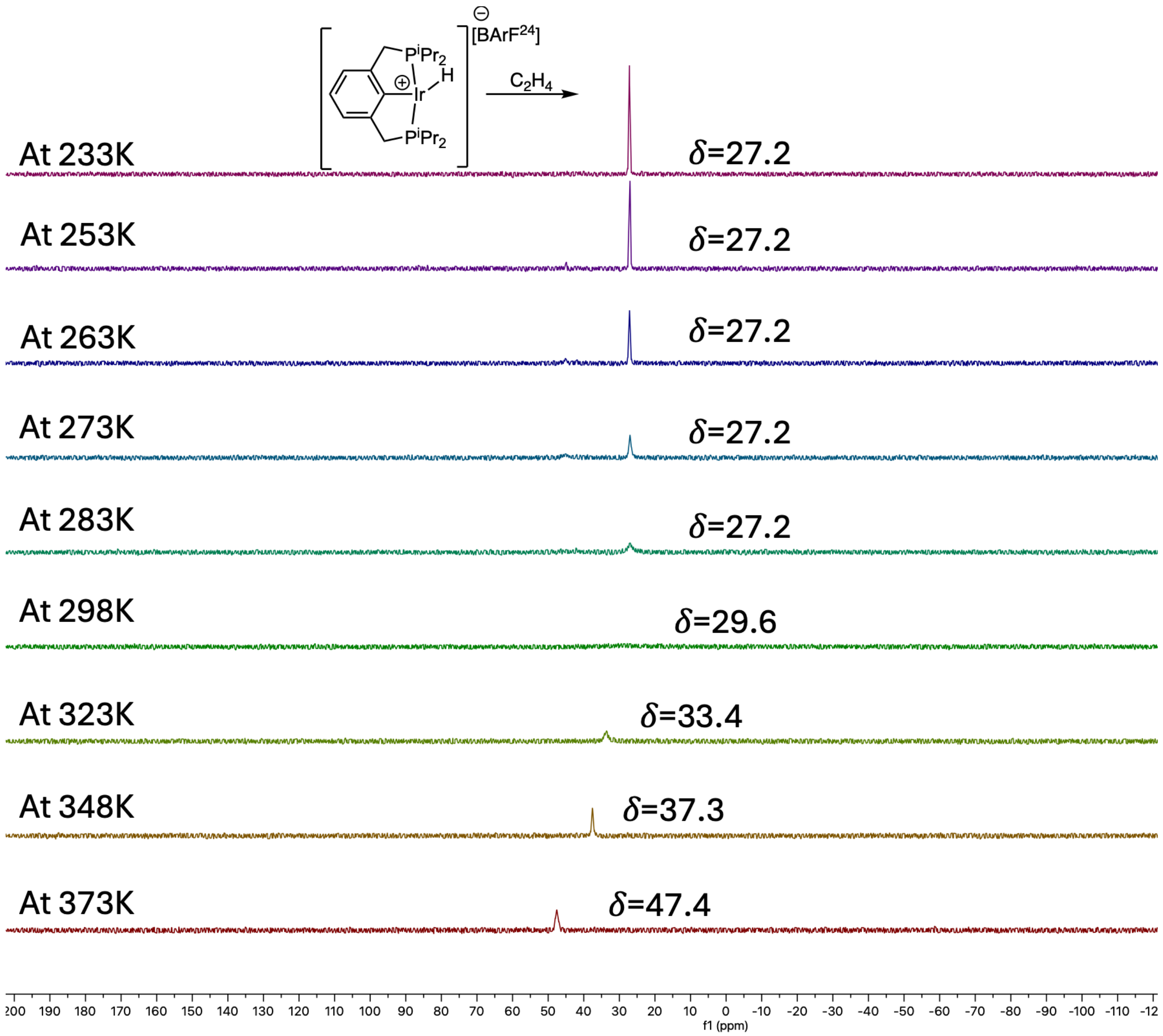

Figure S19. VT ^{31}P NMR Spectra, reaction of [(^{iPr}PCP)IrH][BArF^{24}] with C_2H_4 in toluene-d$_8$.

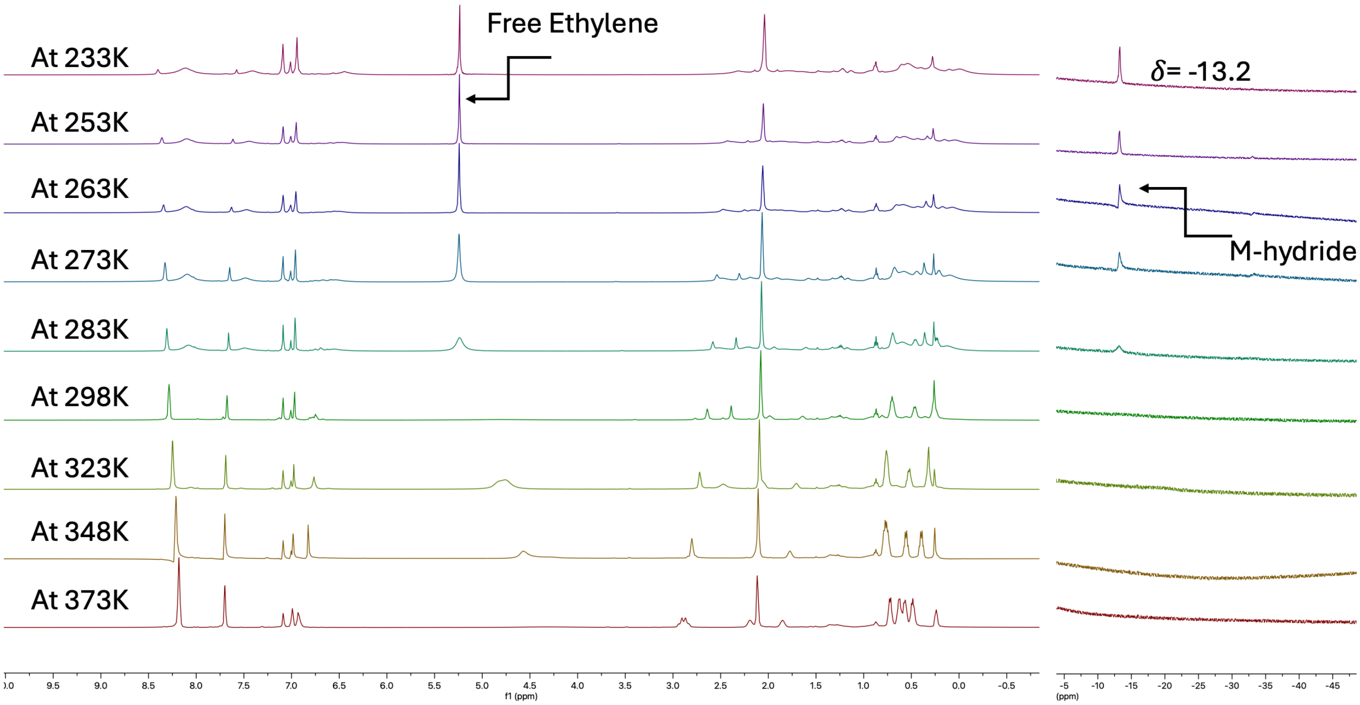

Figure S20. VT ^{1}H NMR Spectra, reaction of [(^{iPr}PCP)IrH][BArF^{24}] with C_2H_4 in toluene-d8.

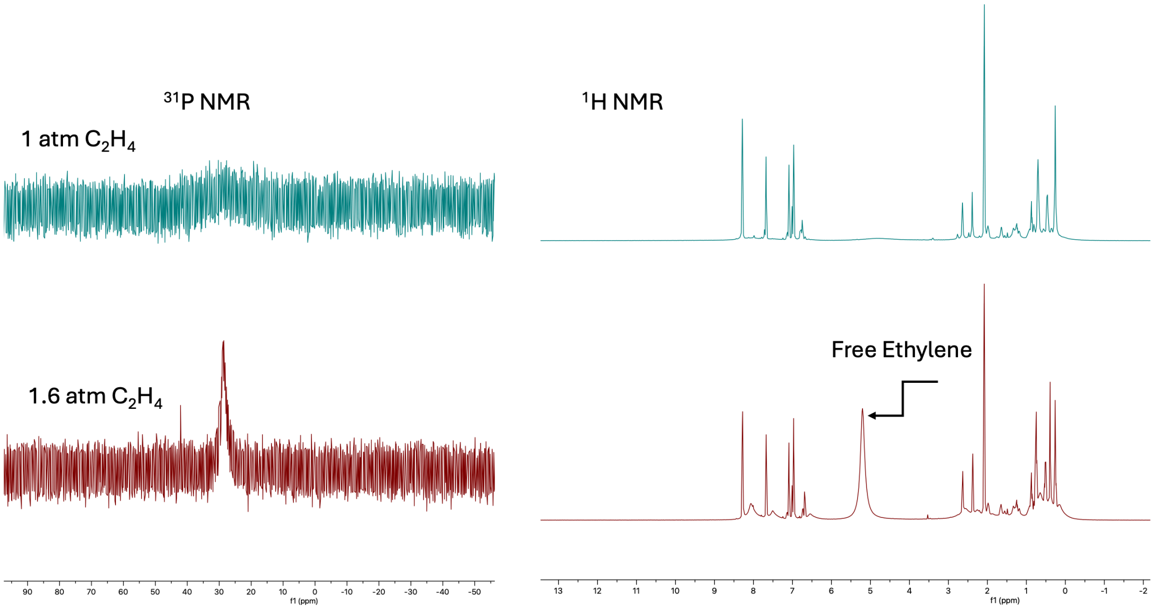

Figure S21. ^{31}P and ^{1}H NMR Spectra, reaction of [(^{iPr}PCP)IrH][BArF^{24}] with variable pressure of C_2H_4 in toluene-d_8.

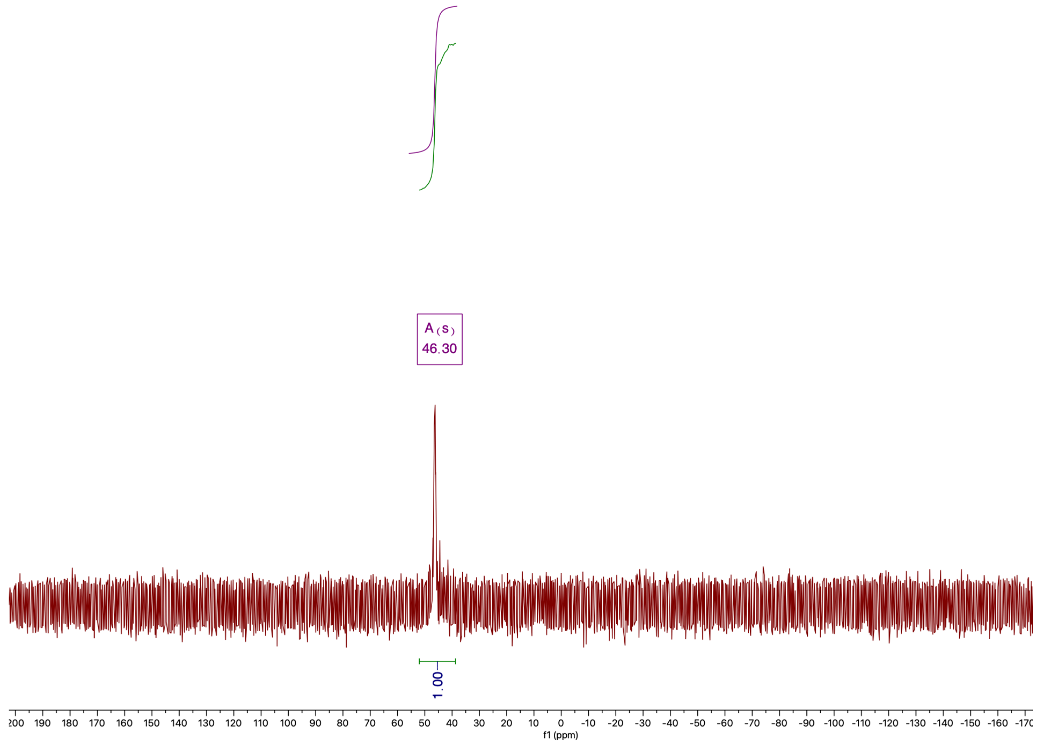

Figure S22. $^{31}P\{^{1}H\}$ NMR Spectrum of $[(^{iPr}PCP)IrH(C_3H_6)][BArF^{24}]$ in toluene-d_8 at 298 K

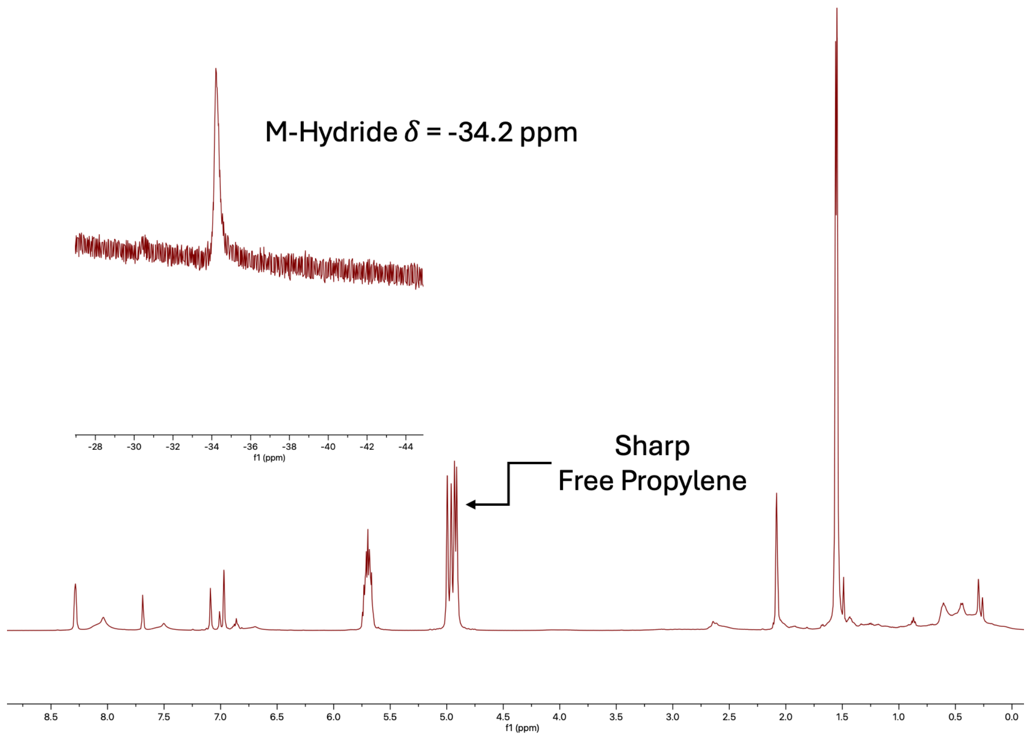

Figure S23. ^{1}H NMR Spectrum of $[(^{iPr}PCP)IrH(C_3H_6)][BArF^{24}]$ in toluene-d_8 at 298 K

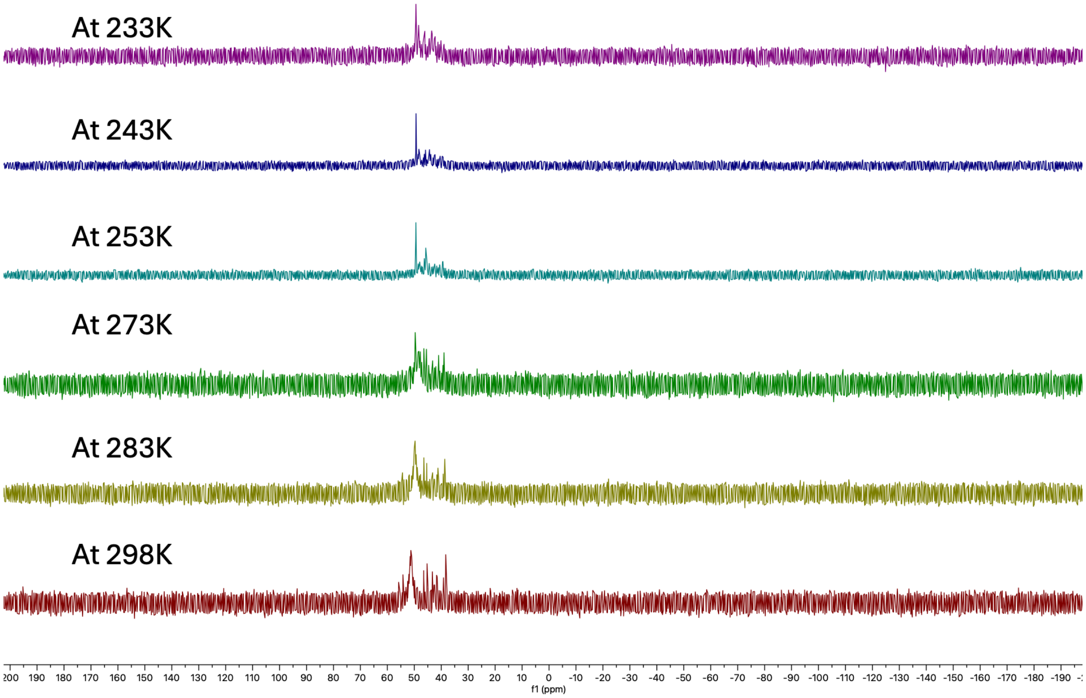

Figure S24. VT $^{31}P\{^1H\}$ NMR Spectra, reaction of [(^{iPr}PCP)IrH][BArF^{24}] with TBE in toluene-d_8.

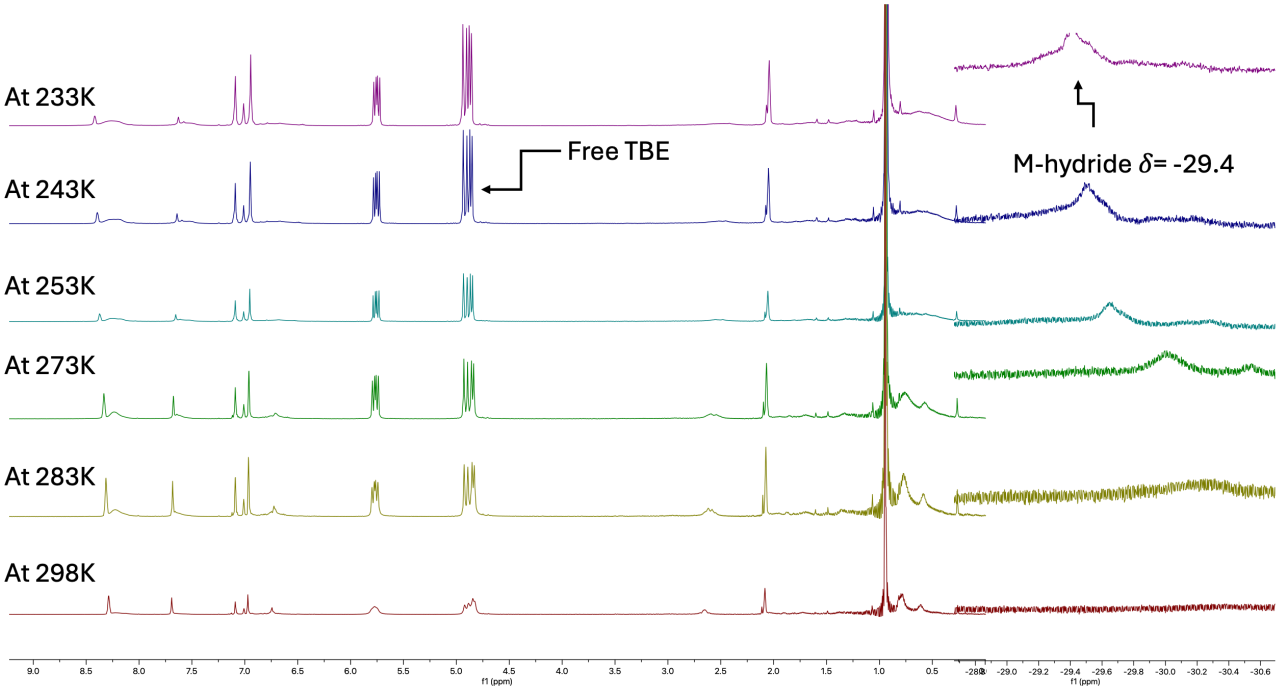

Figure S25. VT ^1H NMR Spectra, reaction of [(^{iPr}PCP)IrH][BArF^{24}] with TBE in toluene-d_8.

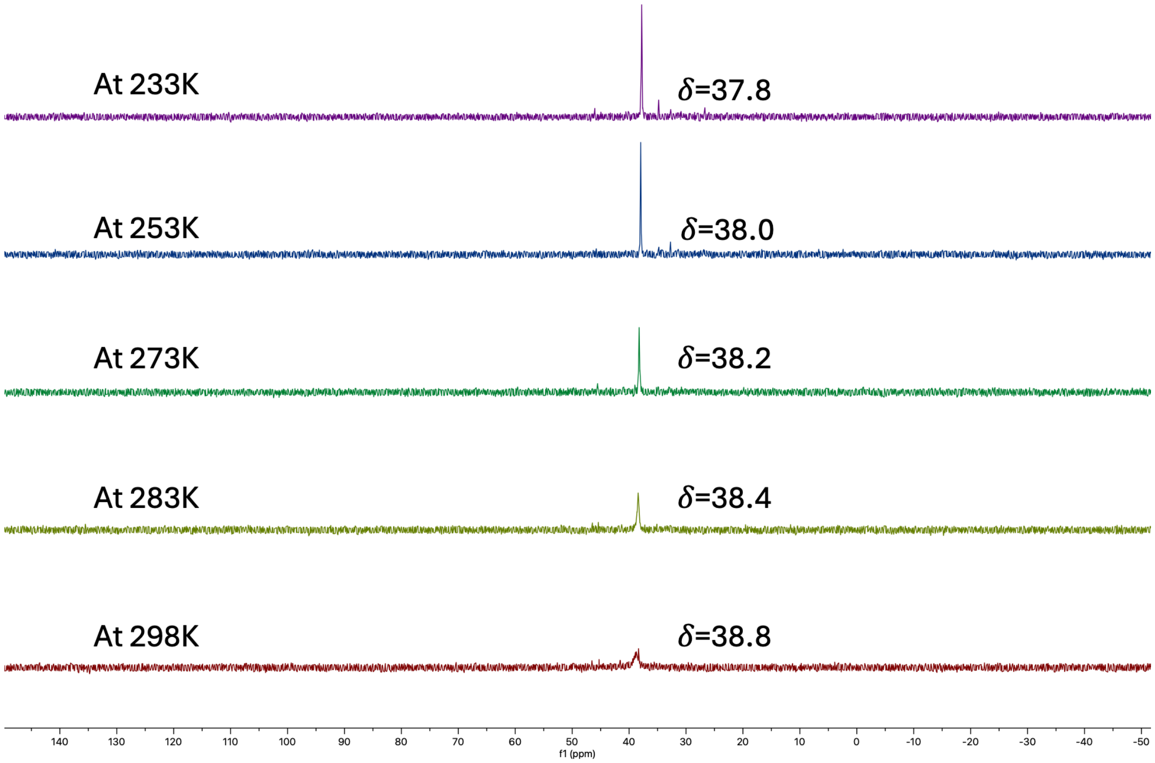

Figure S26. VT $^{31}P\{^{1}H\}$ NMR Spectra, reaction of [(^{iPr}PCP)IrH][BArF^{24}] with CPE in toluene-d$_8$.

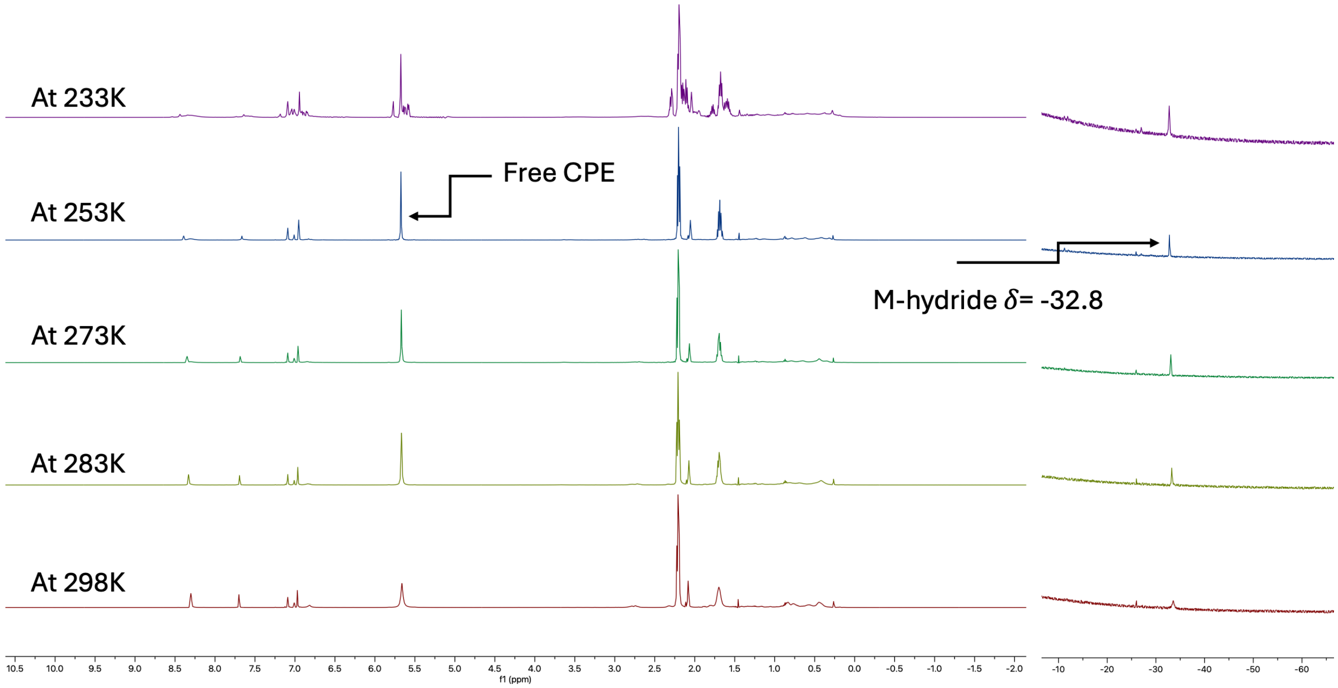

Figure S27. VT ^{1}H NMR Spectra, reaction of [(^{iPr}PCP)IrH][BArF^{24}] with CPE in toluene-d$_8$.

S4. Transfer Dehydrogenation Data

S4.a. Optimization of catalytic conditions for COA/TBE dehydrogenation by (^{iPr}PCP)IrH$^+$:

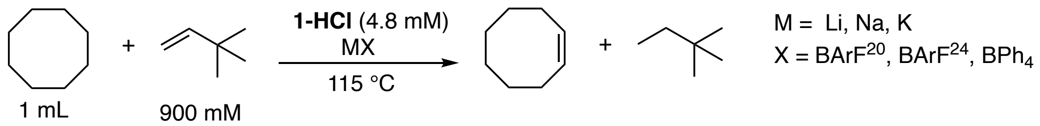

Table S1. Various cationic and anion sources used for COA/TBE. Cat. (iPrPCPIrH$^+$= 4.8 mM), 115 °C, COE (in mM)

Time/min	0	10	30	60	120	240	1080
LiBArF^{20}	0	59.2	136.2	208.6	265.2	348.4	564.0
$LiBPh_4$	0	29.4	75.3	187.6	260.3	358.0	445.1
NaBArF^{24} (Anhydrous)	0	53.3	202.5	275.8	339.4	411.4	648.2
NaBArF^{24} (1-3 % water)	0	111.1	186.6	282.8	355.8	430.3	558.0
$KBPh_4$	0	0	0	0	0	0	0

Table S2. Various concentration of Li[BArF^{20}] used for COA/TBE. Cat. (iPrPCPIrH$^+$= 4.8 mM), 115 °C, COE (in mM)

Time/min	0	10	30	60	120	240	1080
3 equiv LiBArF^{20}	0	92.5	184.0	268.9	353.1	464.4	615.0
6 equiv LiBArF^{20}	0	121.8	211.5	282.6	343.5	423.5	487.8
1 equiv LiBArF^{20}	0	59.2	136.2	208.5	265.2	348.4	564.0
0 equiv LiBArF^{20}	0	0	0	0	0	0	0
0.2 equiv LiBArF^{20}	0	22.4	32.6	22.9	32.1	35.0	35.2
0 equiv Catalyst	0	0	0	0	0	0	0

S4.b. Screening of acceptor for alkane dehydrogenation by (^{iPr}PCP)IrH$^+$:

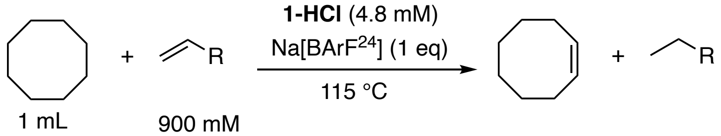

Table S3. Various acceptors used for COA dehydrogenation. Cat. (iPrPCPIrH$^+$= 4.8 mM), 115 °C, COE (in mM)

Time/min	0	10	30	60	120	240
TBE	0	111.07	186.63	282.79	355.84	430.27
TBPE	0	15.12	31.9	64.36	82.85	88.76
hexene	0	15.52	30.48	54.37	55.39	56.87
cyclohexene	0	33.16	62.81	65.64	68.8	84.15

S4.c. Various catalysts used for COA/TBE dehydrogenation:

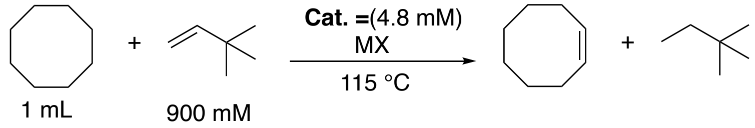

Table S4. Various catalysts (4.8 mM) used for COA/TBE dehydrogenation. 115 °C, COE (in mM)

Time/min	0	10	30	60	120	240
(^{iPr4}PCP)IrH$^+$	0	111.07	186.63	282.79	355.84	430.27
(^{tBu2}PCOP^{iPr2})IrH$^+$	0	48.68	52.66	58.12	63.12	73.02
(^{Ad4}PCP)IrH$^+$	0	0	0	15.12	30.05	31.2
(^{tBu4}PCP)IrH$^+$	0	0	0	0	0	0

S4.d. Competition COA & n-decane /TBE dehydrogenation catalyzed by (iPrPCP)Ir(I) and (iPrPCP)IrH$^+$:

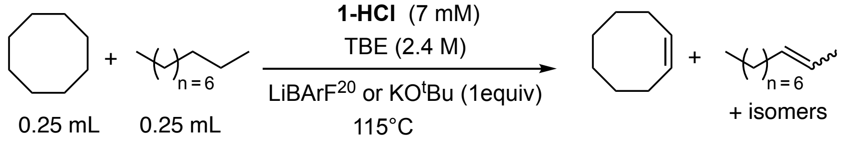

Table S5. (^{iPr}PCP)Ir(I)(7 mM) for COA & n-decane/TBE competition dehydrogenation. 115 °C, COE & decenes (in mM)

Time/min	0	10	30	60	120
decenes	0	63.01	88.1	204.84	295.93
COE	0	36.61	46.63	92.27	130.39

Table S6. (^{iPr}PCP)IrH$^+$ (7 mM) for COA & n-decane/TBE competition dehydrogenation. 115 °C, COE & decenes (in mM)

Time/min	0	10	30	60	120
COE	24.64	91.04	143.2	174.04	213.35
decenes	0	0	0	0	0

S4e. Competition COA & n-decane /TBE dehydrogenation catalyzed by (^{iPr}PCP)Ir(I) + HBArF^{24}:

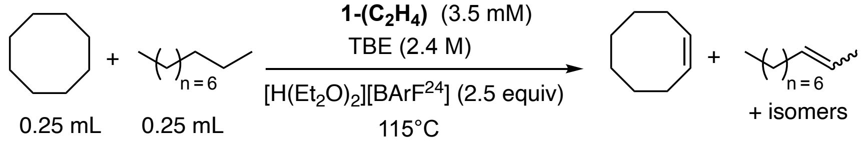

Table S7. (^{iPr}PCP)IrH$^+$ (7 mM) for COA & n-decane/TBE competition dehydrogenation. 115 °C, COE & decenes (in mM)

Time/min	0	10	30	60	120
COE	20.96	69.88	116.65	134.5	166.38
decenes	0	0	0	0	2.06

S3.f. Competition CPA & n-decane /TBE dehydrogenation catalyzed by (^{iPr}PCP)IrH$^+$:

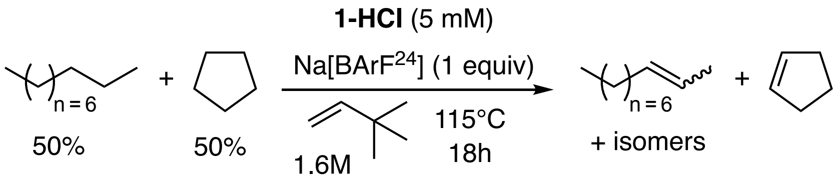

Table S8. (^{iPr}PCP)IrH$^+$ (5 mM) for CPA & n-decane/TBE competition dehydrogenation. 115 °C, CPE & decenes (in mM)

Time/min	0	10	30	60	300	1080
CPE	0	8.1	10.1	12.2	17	26.2
decenes	0	0	0	0	0	2.04

S3g. Competition CDA & n-decane /TBE dehydrogenation catalyzed by (^{iPr}PCP)IrH$^+$:

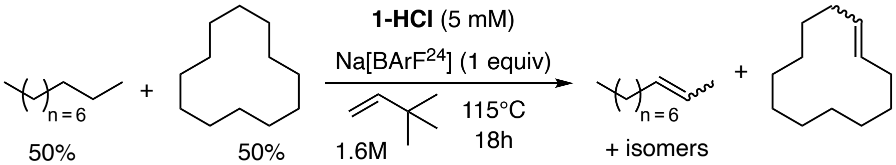

Table S9. (^{iPr}PCP)IrH$^+$ (5 mM) for CDA & n-decane/TBE competition dehydrogenation. 115 °C, CDE & decenes (in mM)

Time/min	0	10	30	60	120	240
decenes	0	3.04	7.32	8.58	9.31	12.56
CDE	0	0	0	0.68	0.69	0.68

S3h. Competition CDA & n-decane /TBE dehydrogenation catalyzed by (^{iPr}PCP)IrH$^+$:

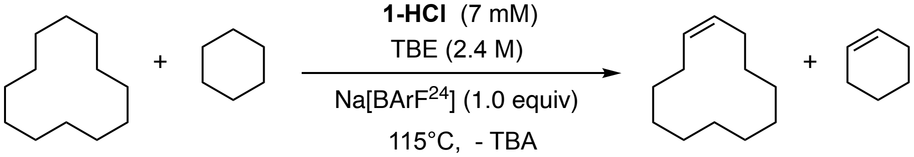

Table S10. (^{iPr}PCP)IrH$^+$ (7 mM) for CDA & CHA/TBE competition dehydrogenation. 115 °C, CDE & CHE (in mM)

Time/min	0	90	180	1050	1560
CDE	0	1.11	1.2	2.39	3.34
CHxE	0	0	0	0	0

S3.i. Competition COA & CPA /TBE dehydrogenation catalyzed by (^{iPr}PCP)IrH$^+$:

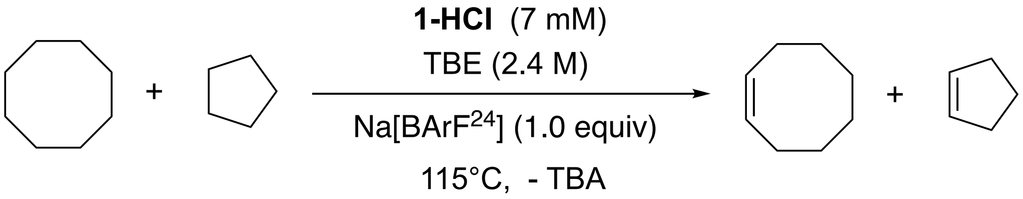

Table S11. (^{iPr}PCP)IrH$^+$ (7 mM) for COA & CPA/TBE competition dehydrogenation. 115 °C, COE & CPE (in mM)

time/min	0	30	60	90	210	1080
COE	0	22.84	32.63	49.01	71.65	132.18
CPE	0	0	0	0	4.35	22.55

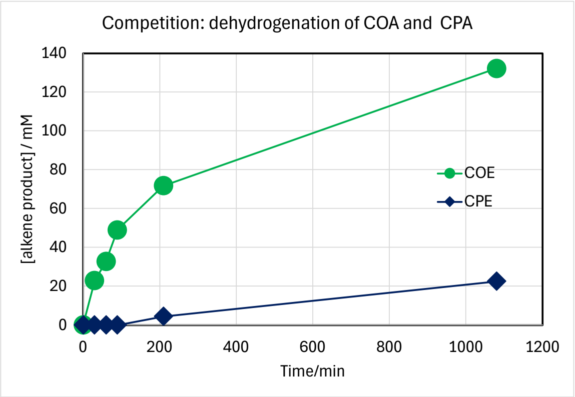

S3.j. Control experiment for COA/TBE dehydrogenation using H[BArF^{24}]:

Table S12. (^{iPr}PCP)IrH$^+$ (3.5 mM) for COA/TBE dehydrogenation and control with H[BArF^{24}] 115 °C, COE & CPE (in mM)

Time/min	0	10	30	60	120
(PCP)Ir(I) + HBArF^{24}	0	48.92	95.69	113.54	145.42
HBArF^{24}	0	10.4	17.5	16.93	7.5

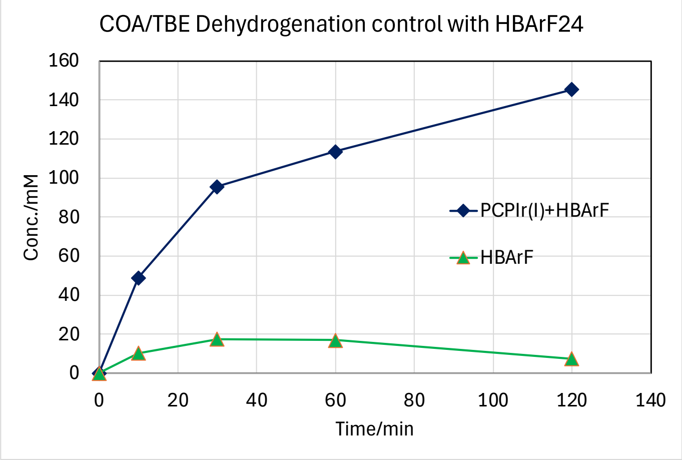

S5. Competition Hydrogenation Data

Competition between COE and TBE using (^{iPr}PCP)IrH$^+$:

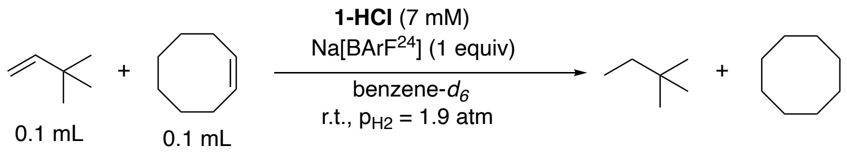

Table S13. Data for hydrogenation competition between COE and TBE catalyzed by (^{iPr}PCP)IrH$^+$

No. of times H_2 Charged	0	1	2	3	7	12
COA	0	88.13	151.81	225.13	520.53	861.69
TBA	0	85.64	166.27	245.23	483.99	791.48

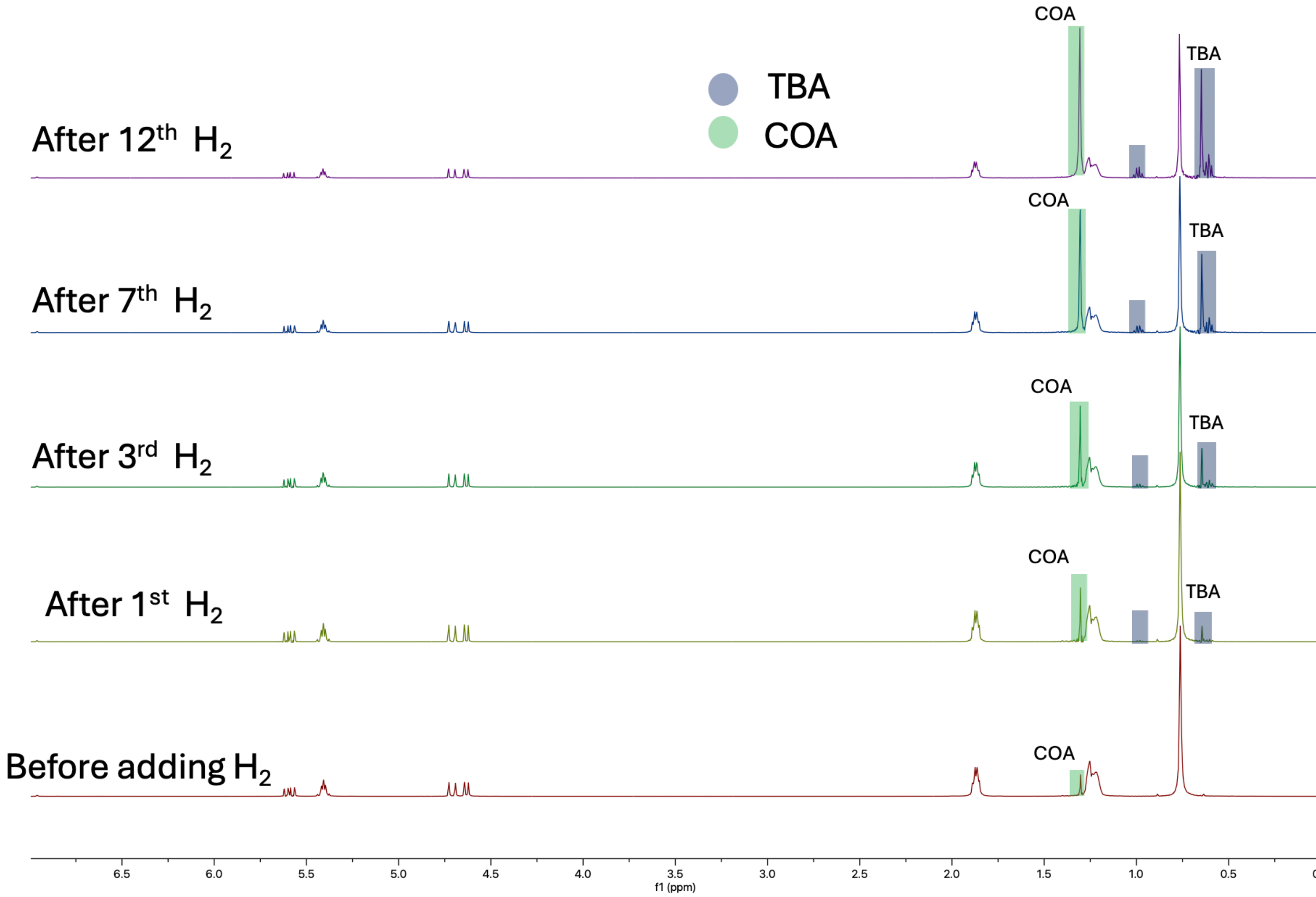

Figure S28. ^{1}H NMR spectra of hydrogenation competition between COE and TBE by (^{iPr}PCP)IrH$^+$ in benzene-d_6

Competition between COE and TBE using (^{iPr}PCP)Ir(I) :

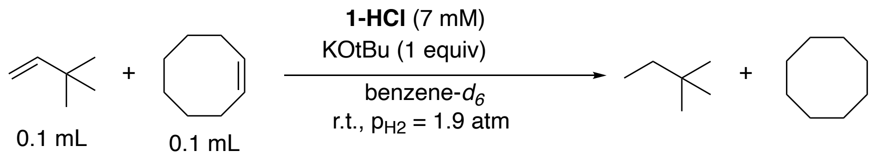

Table S14. Data for hydrogenation competition between COE and TBE catalyzed by (^{iPr}PCP)Ir(I)

No. of times H_2 Charged	0	1	2	3	7
TBA	0	146.3	321.25	436.3	1032.5
COA	84.4	85.3	85.3	85.3	87.18

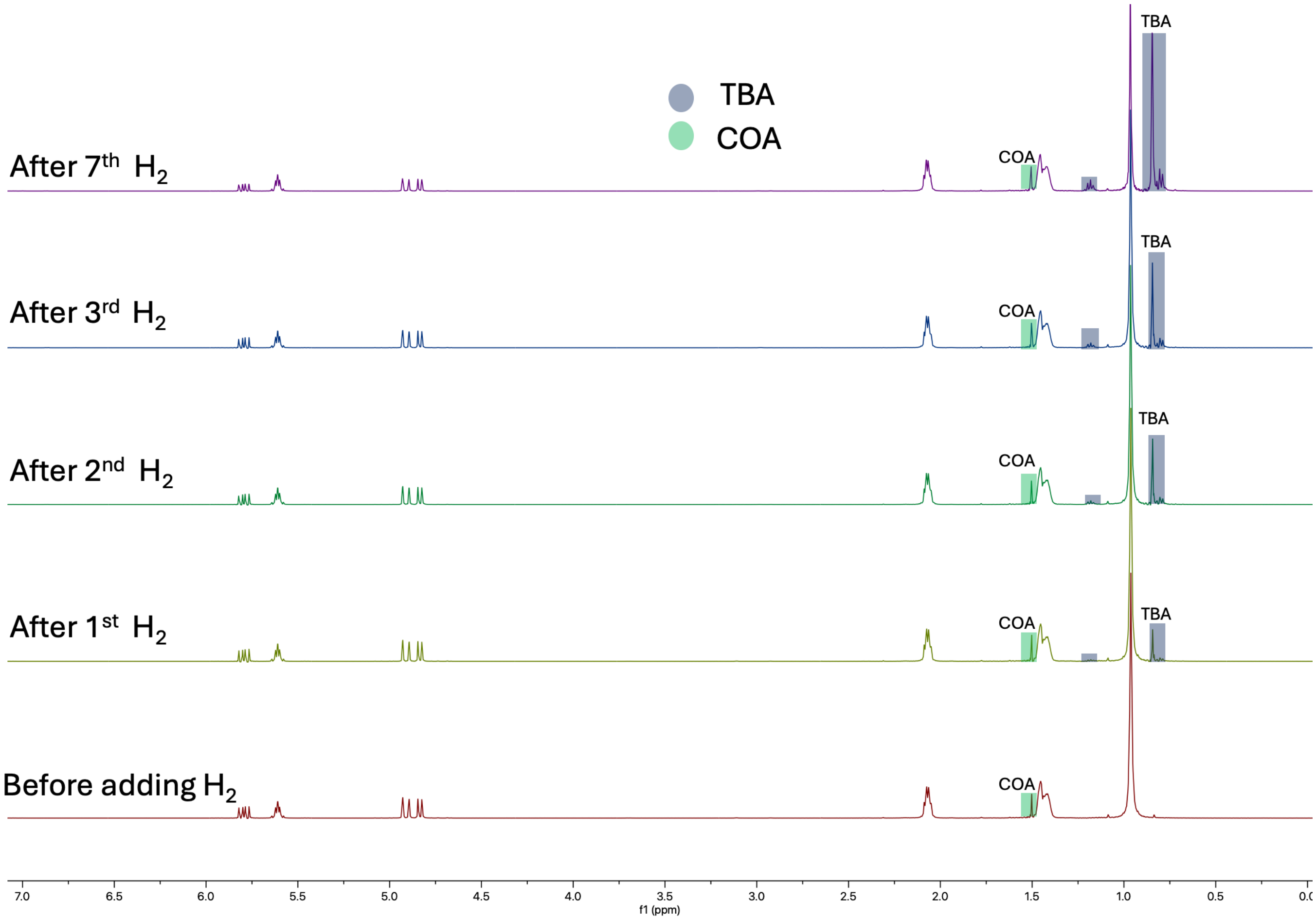

Figure S29. ^{1}H NMR spectra of hydrogenation competition between COE and TBE by (^{iPr}PCP)Ir(I) in benzene-d$_6$

Competition between CPE and TBE using (^{iPr}PCP)IrH$^+$:

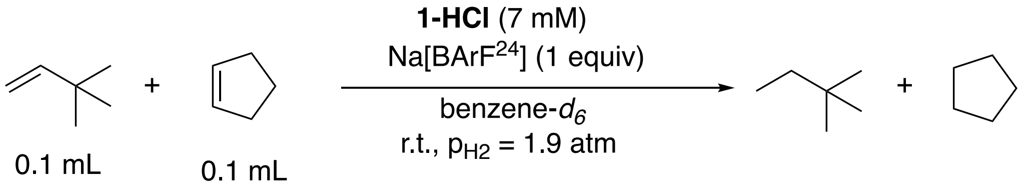

Table S15. Data for hydrogenation competition between CPE and TBE catalyzed by (^{iPr}PCP)IrH$^+$

No. of times H_2 Charged	0	1	2	4	7
CPA	0	36.621	121.29	274.8	536.92
TBA	0	13.454	-31.68	38.86	151.47

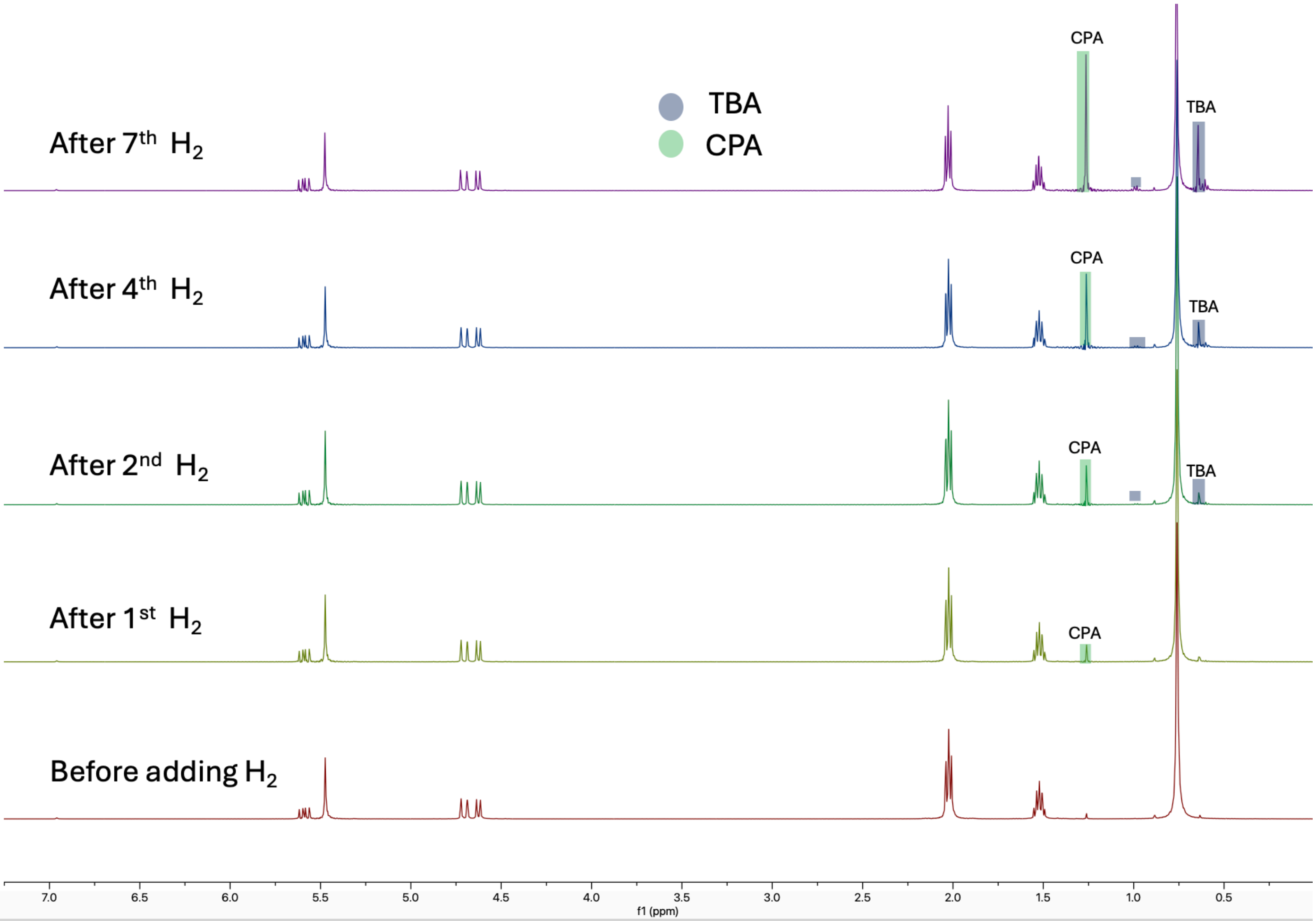

Figure S30. ^{1}H NMR spectra of hydrogenation competition between CPE and TBE by (^{iPr}PCP)IrH$^+$ in benzene-d_6

Competition between CHE and TBE using (^{iPr}PCP)IrH^{+} :

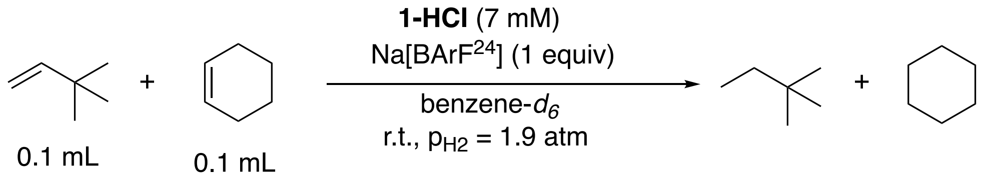

Table S16. Data for hydrogenation competition between CHxE and TBE catalyzed by (^{iPr}PCP)IrH^{+}

No. of times H_2 Charged	0	1	3	5	8
TBA	0	146.51	398.91	635.79	944.95
CHxA	0	6.47	14.14	21.71	26.04

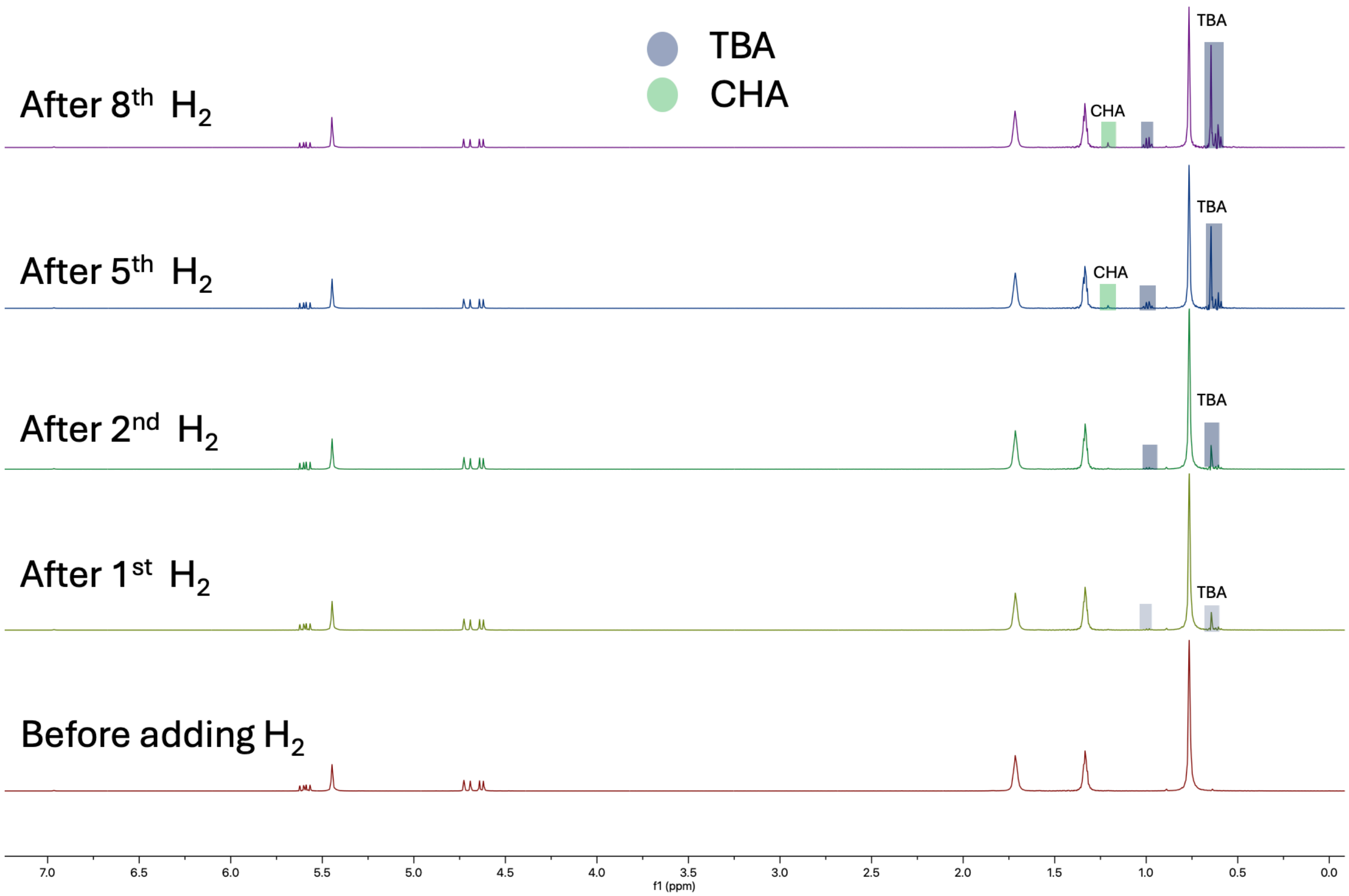

Figure S31. ^{1}H NMR spectra of hydrogenation competition between CHE and TBE by (^{iPr}PCP)IrH^{+} in benzene-d_6

Competition between ChPE and TBE using (^{iPr}PCP)IrH$^+$:

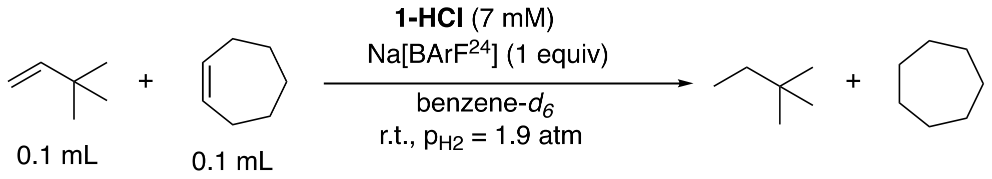

Table S17. Data for hydrogenation competition between CHpE and TBE catalyzed by (^{iPr}PCP)IrH$^+$

No. of times H_2 Charged	0	1	3	6	10
CHpA	0	34.97	102.28	206.24	322.68
TBA	0	8.16	26.65	53.83	82.71

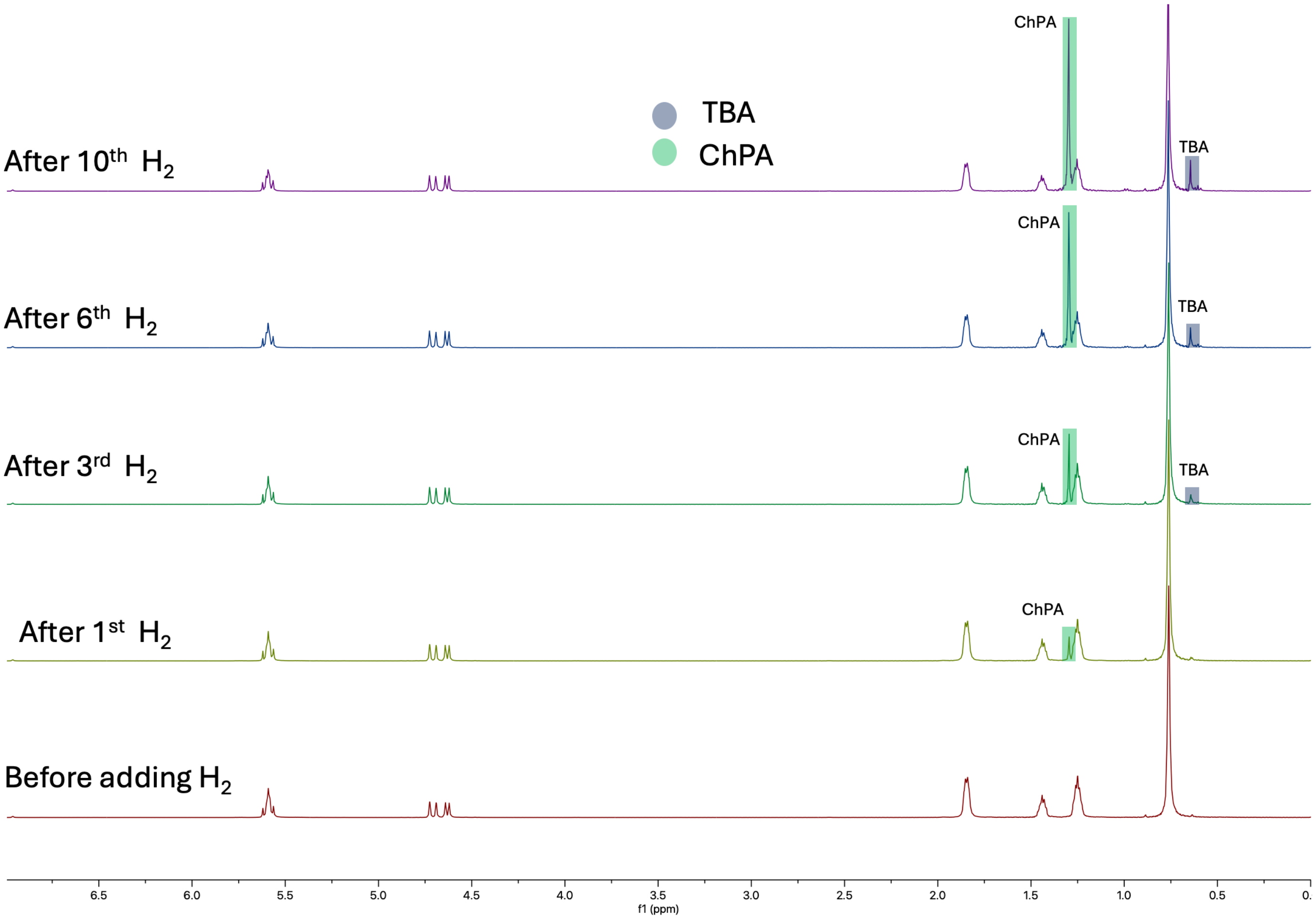

Figure S32. ^{1}H NMR spectra of hydrogenation competition between ChPE and TBE by (^{iPr}PCP)IrH$^+$ in benzene-d$_6$

Competition between COE and TBPE using (^{iPr}PCP)IrH$^+$:

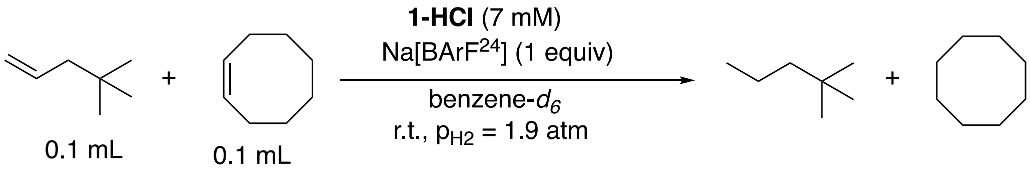

Table S18. Data for hydrogenation competition between COE and TBPE catalyzed by (^{iPr}PCP)IrH$^+$

No. of times H_2 Charged	0	1	3	6	10
TBPA	0	140.08	337.41	679.6	1114.17
COA	0	24.15	51.42	114.79	226.96

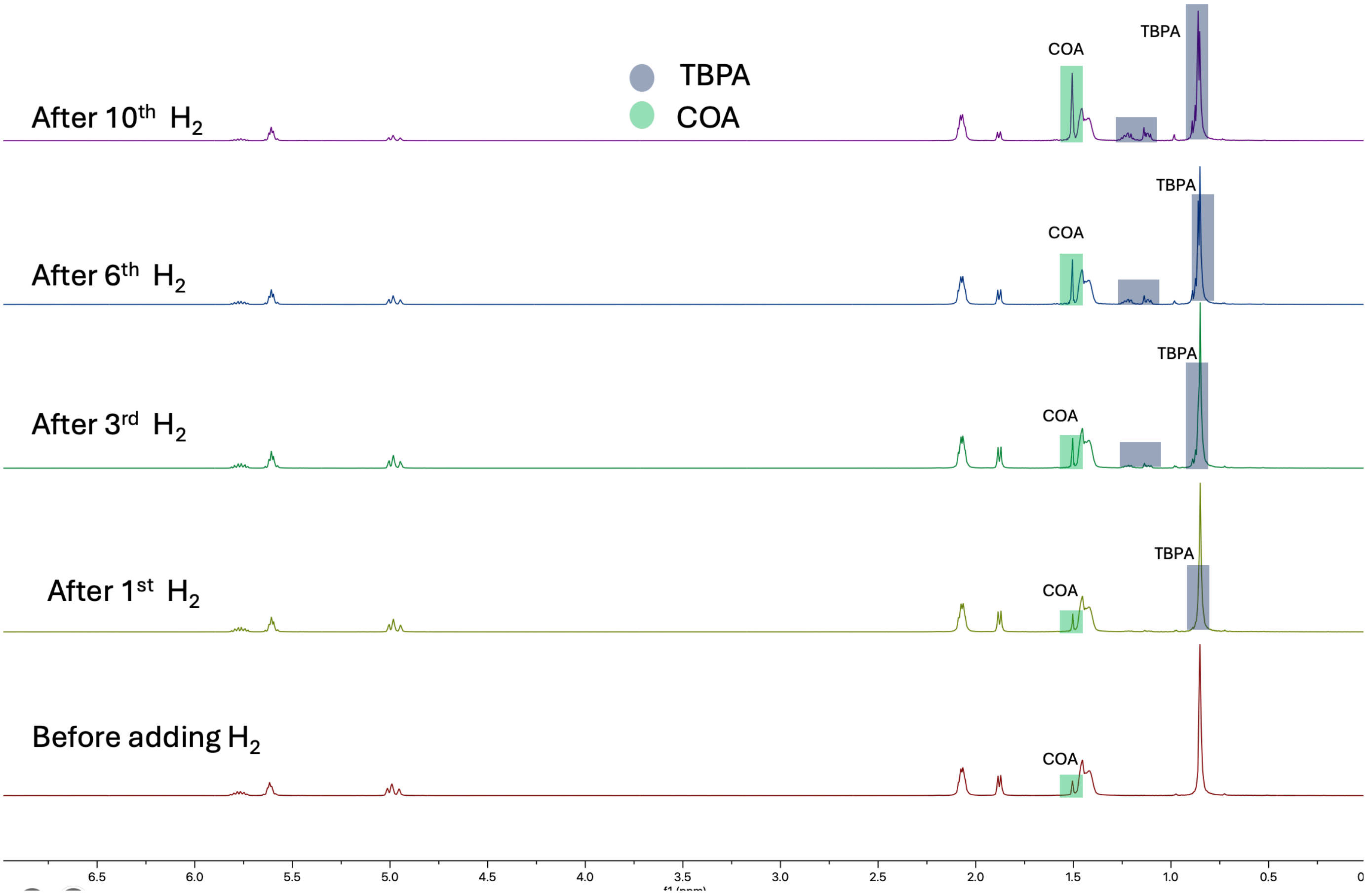

Figure S33. ^{1}H NMR spectra of hydrogenation competition between COE and TBPE by (^{iPr}PCP)IrH$^+$ in benzene-d_6

Competition between COE and CPE using (^{iPr}PCP)IrH$^+$:*

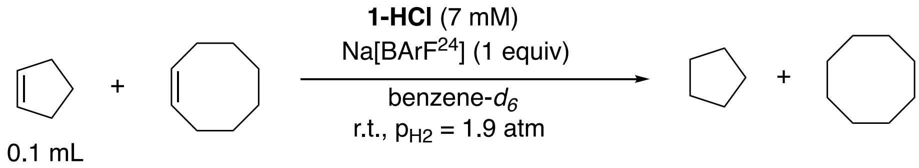

Table S19. Data for hydrogenation competition between CPE and COE catalyzed by (^{iPr}PCP)IrH$^+$

No. of times H_2 Charged	0	1	3	4	7	11
CPA	0	118.84	396.21	567.49	1062.08	1763.18
COA	0	103.57	278.38	389.49	729.98	1180.69

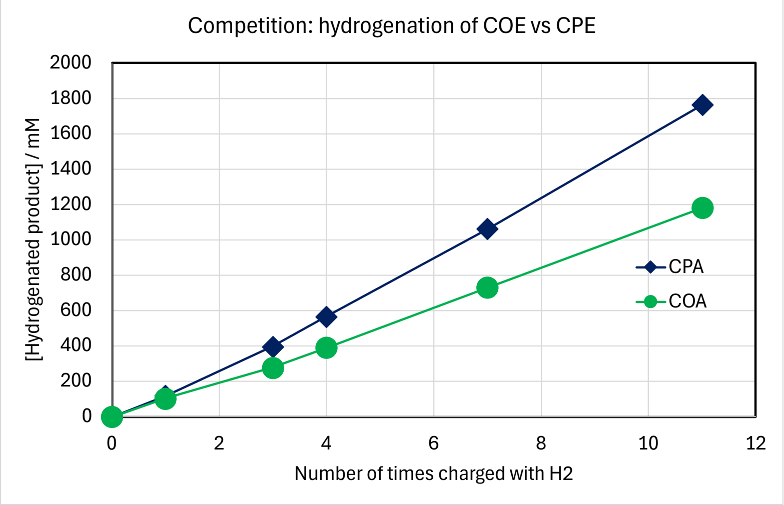

Figure S34. Hydrogenation competition between COE and CPE by (^{iPr}PCP)IrH^{+}*

**Analysis done using GC as in ^{1}H NMR spectrum COA and CPA peak were overlapping and hindering the analysis.*

S6 Competition H/D Exchange Data

Competition between benzene and CHA H/D exchange using (^{iPr}PCP)IrH$^+$ and H_2:

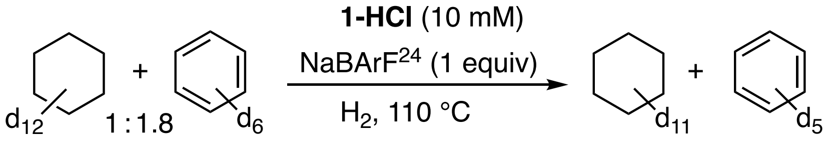

Table S20. Data for H/D exchange competition between benzene and CHA catalyzed by (^{iPr}PCP)IrH$^+$

Time/min	0	90	1020	2460
benzene-d5H	0	1	27	35
cyclohexane-d11H	0	0	6	7

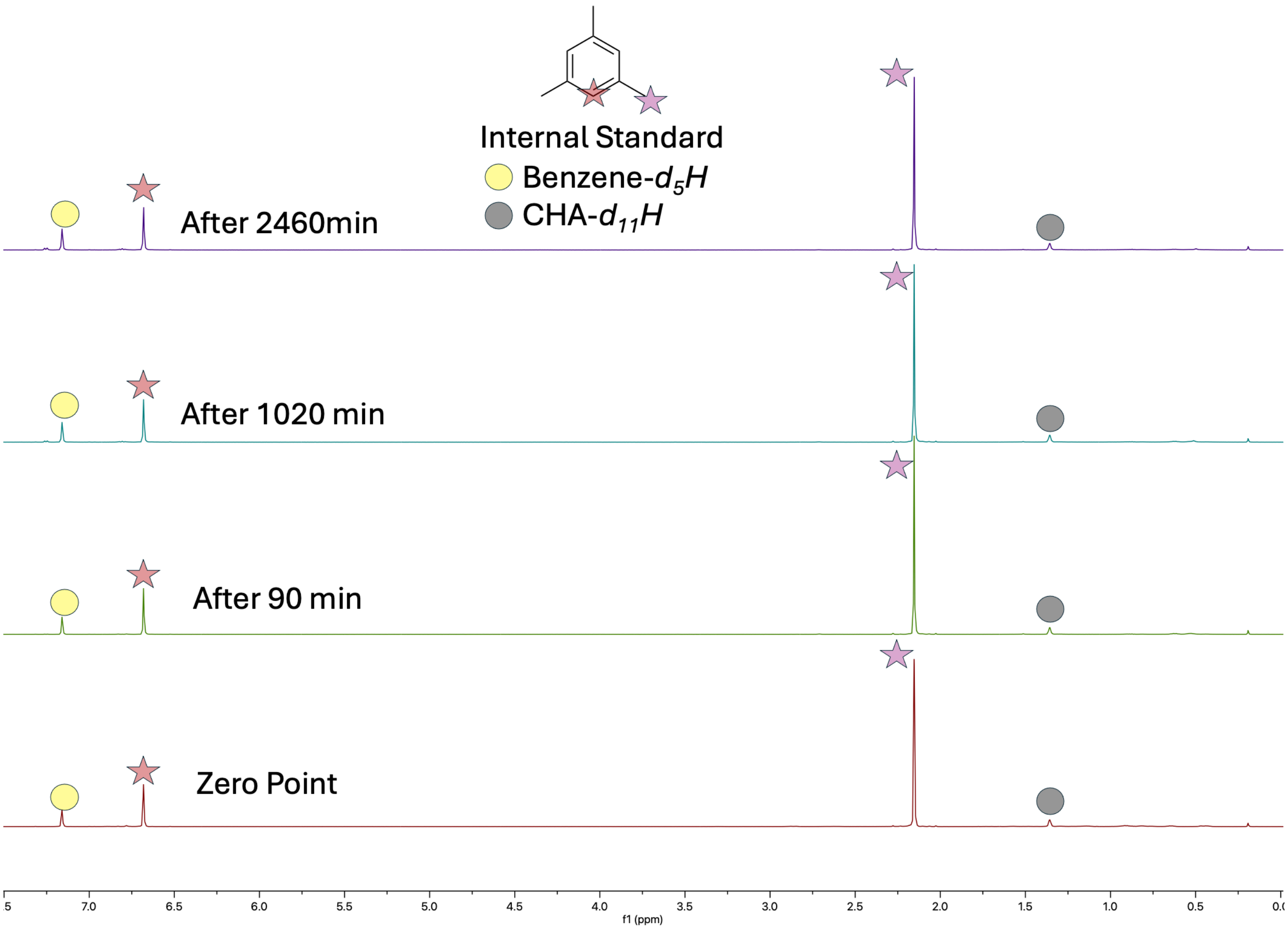

Figure S35. ^{1}H NMR spectra of H/D exchange competition between benzene and CHA by (^{iPr}PCP)IrH$^+$ in benzene-d_6: CHA-d_{12} (0.3 mL : 0.2 mL) using Mesitylene as internal standard.

Competition between benzene and n-octane H/D exchange using (^{iPr}PCP)IrH$^+$ and H_2:

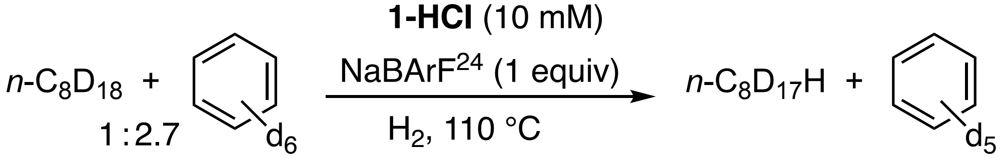

Table S21. Data for H/D exchange competition between benzene and CHA catalyzed by (^{iPr}PCP)IrH$^+$

Time/min	0	60	540	1320	3960
benzene-d5	0	0	6	12	62
n-octane-d17 (internal h)	0	0	4	8	34
n-octane-d17 (terminal h)	0	0	4	6	20

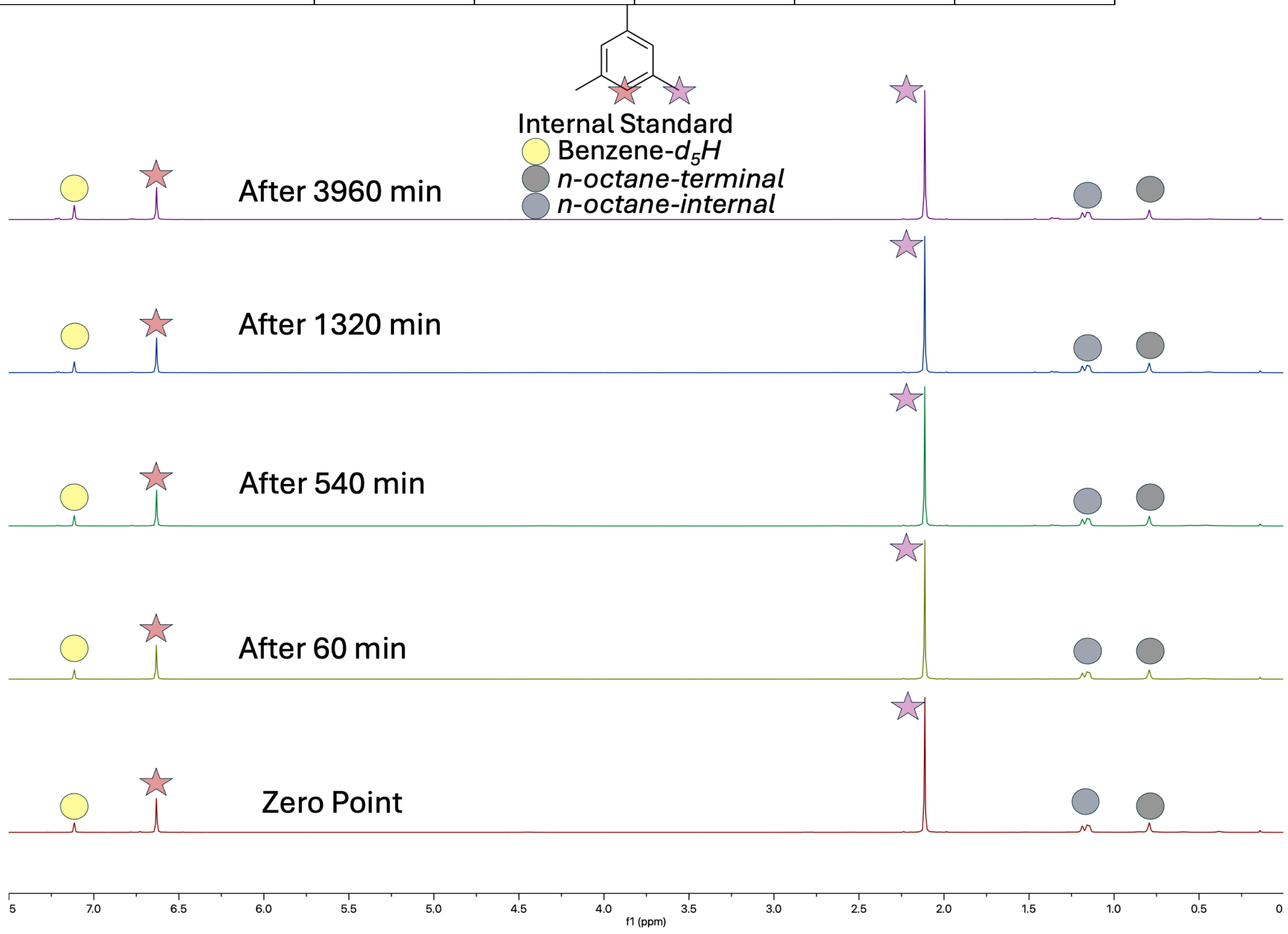

Figure S36. ^{1}H NMR spectra of H/D exchange competition between benzene and n-octane by (^{iPr}PCP)IrH$^+$ in benzene-d_6: n-octane-d_{18} (0.3 mL : 0.2 mL) using Mesitylene as internal standard.

Competition between benzene and COA H/D exchange using (^{iPr}PCP)IrH$^+$ and H_2:

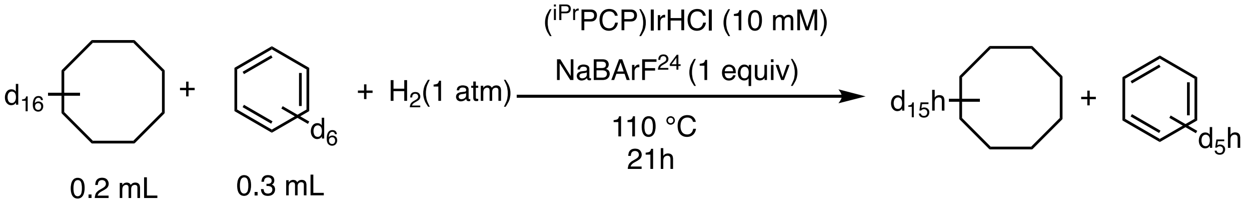

Table S22. Data for H/D exchange competition between benzene and COA catalyzed by (^{iPr}PCP)IrH$^+$

Time/min	0	120	300	440	1260
COA-d15h	0	75	110	143	251
benzene-d5h	0	0	0	2	8

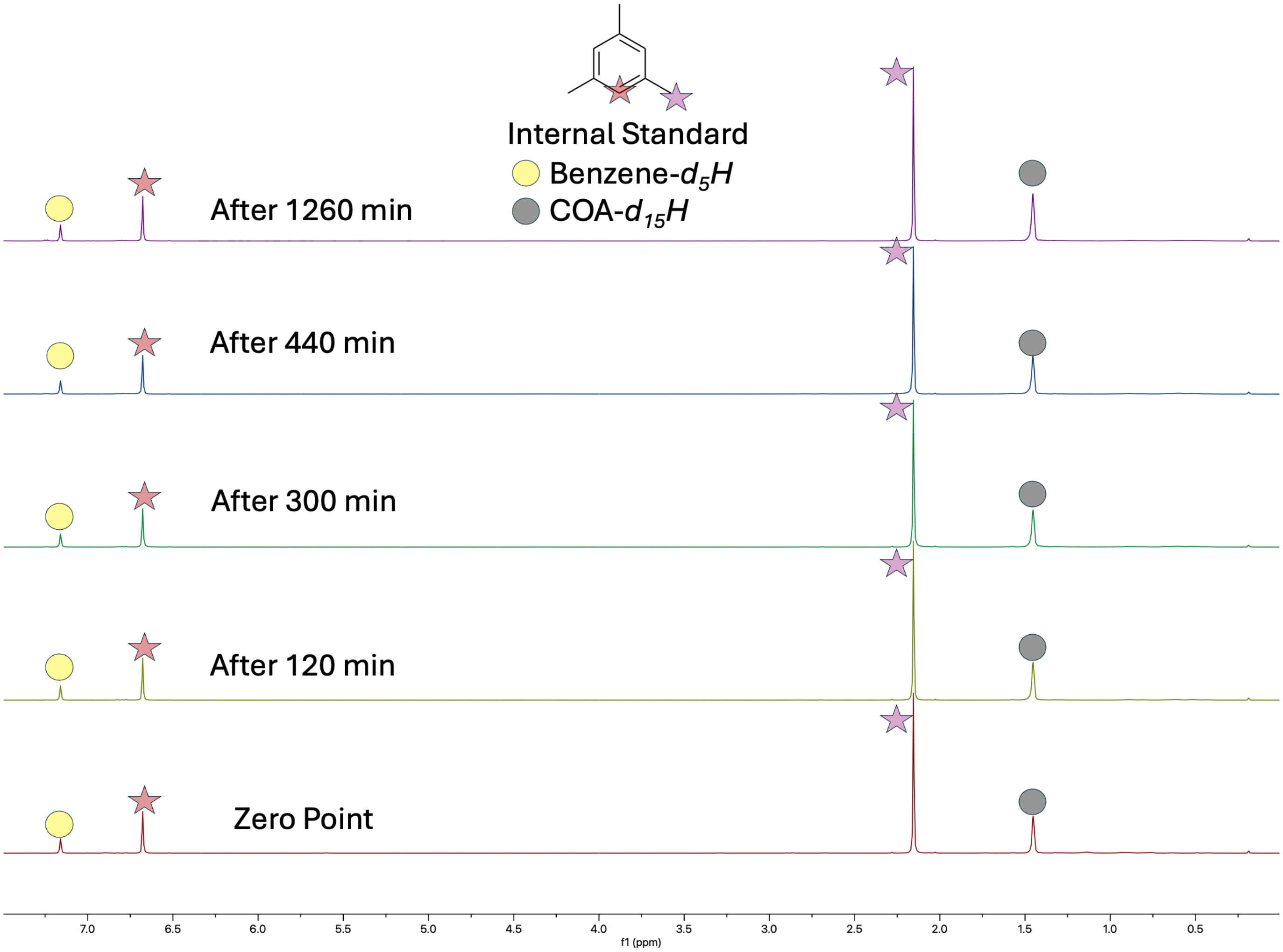

Figure S37. ^{1}H NMR spectra of H/D exchange competition between benzene and COA by (^{iPr}PCP)IrH$^+$ in benzene-d$_6$: COA-d$_{16}$ (0.3 mL : 0.2 mL) using Mesitylene as internal standard.

Competition between benzene and COA H/D exchange using (^{iPr}PCP)IrH$^+$ and dioxane:

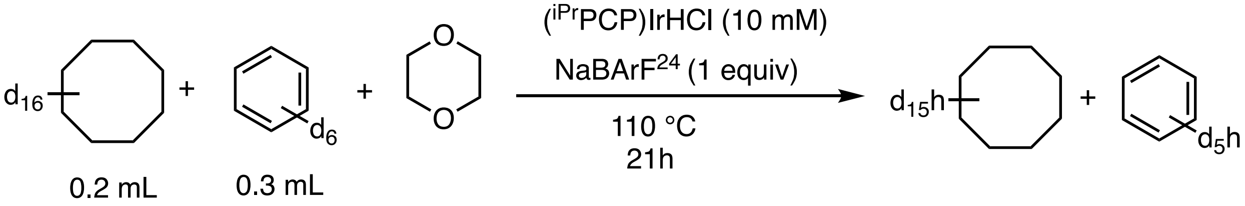

Table S23. Data for H/D exchange competition between benzene and COA catalyzed by (^{iPr}PCP)IrH$^+$

Time	0	120	300	1320	1800	2640
COA-d15h	0	121.44	152.24	334.4	361.68	400.4
benzene-d5h	0	5.28	14.96	22.88	22.88	22.88

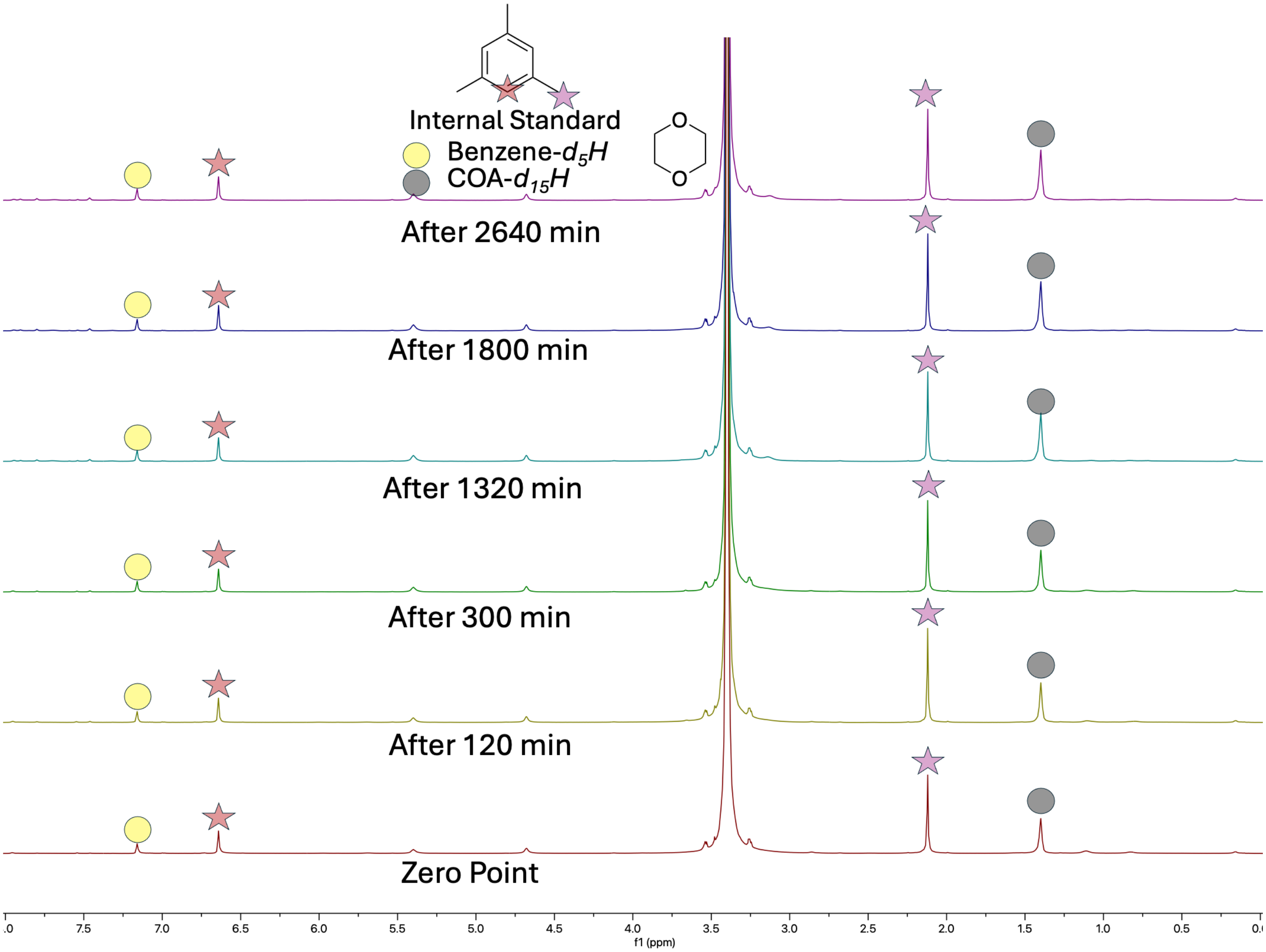

Figure S38. ^{1}H NMR spectra of H/D exchange competition between benzene and COA by (^{iPr}PCP)IrH$^+$ in benzene-d$_6$: COA-d$_{16}$ (0.3 mL : 0.2 mL) using Mesitylene as internal standard.

Competition between intramolecular H/D exchange of toluene using (^{iPr}PCP)IrH^{+} and COA:

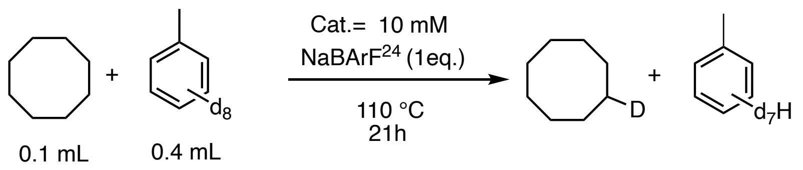

Table S24. Data for intramolecular competition H/D exchange of toluene with COA catalyzed by (^{iPr}PCP)IrH^{+}

Time/min	0	90	240	420	1260
meta-H	0	25	51	90	343
para-H	0	7	22	45	182
benzylic-H	0	2	14	18	53
ortho-H	0	0	0	0	0

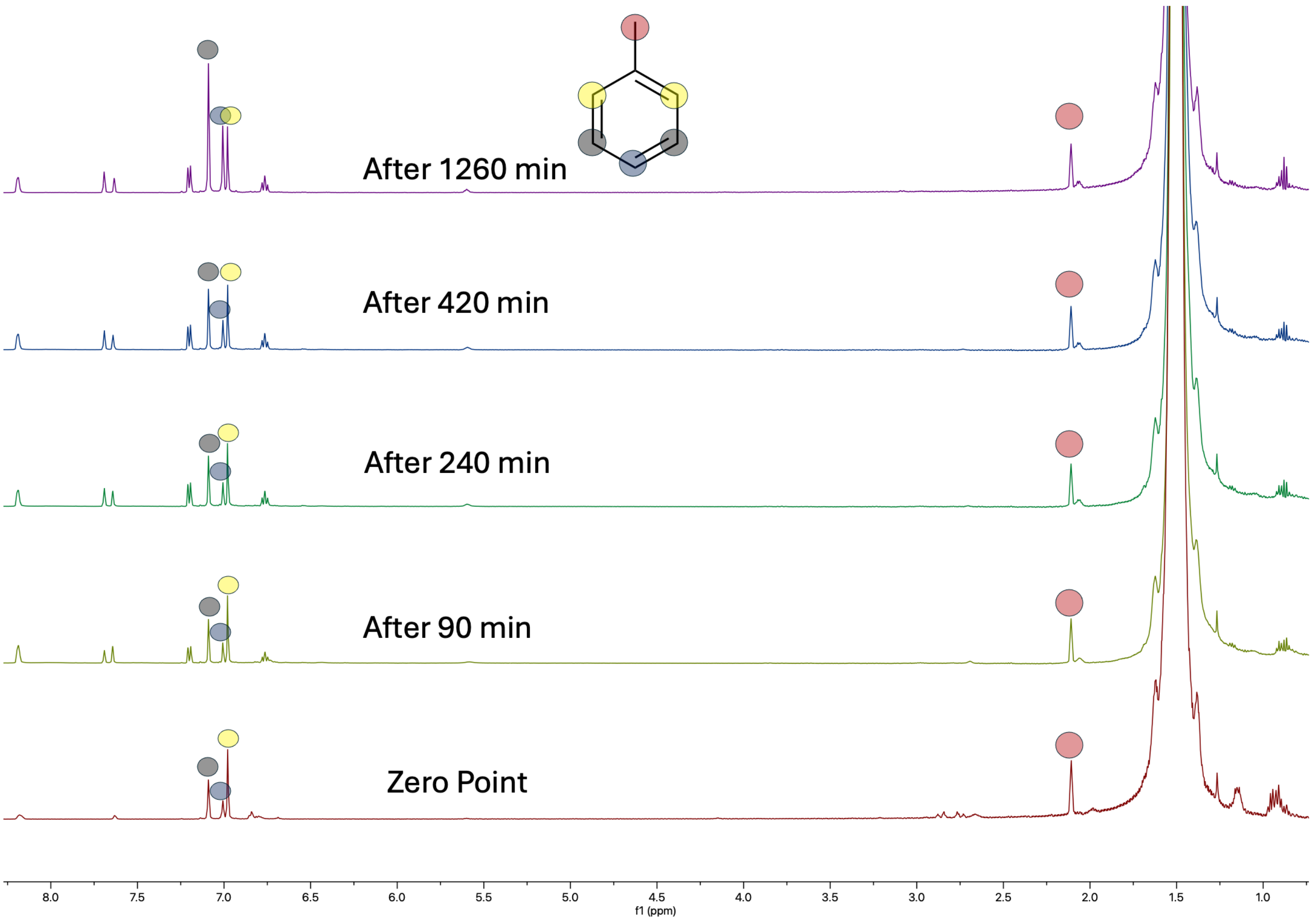

Figure S39. ^{1}H NMR spectra of H/D exchange intramolecular competition of toluene with COA by (^{iPr}PCP)IrH^{+} in toluene-d_8 : COA-h_{16} (0.4 mL : 0.1 mL) using ortho protons as standard.

Competition between intramolecular H/D exchange of ethylbenzene using (^{iPr}PCP)IrH^{+} and COA:

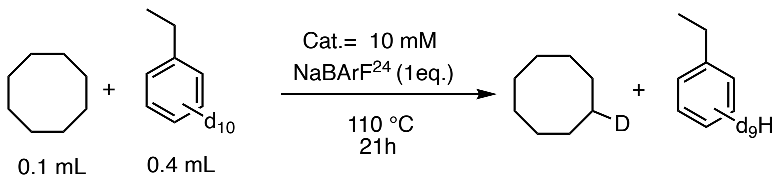

Table S25. Data for intramolecular competition H/D exchange of toluene with COA catalyzed by (^{iPr}PCP)IrH^{+}

Time/min	0	45	135	870	1268	2120
terminal-H	0	18.52	49.91	119.86	168.29	238.15
benzylic-H	0	3.31	10.92	43.86	68.64	111.42
meta-H	0	3.51	6.37	25.28	42.77	74.2
para-H	0	5.77	4.48	7.51	20.37	35.76
ortho-H	0	0	0	0	0	0

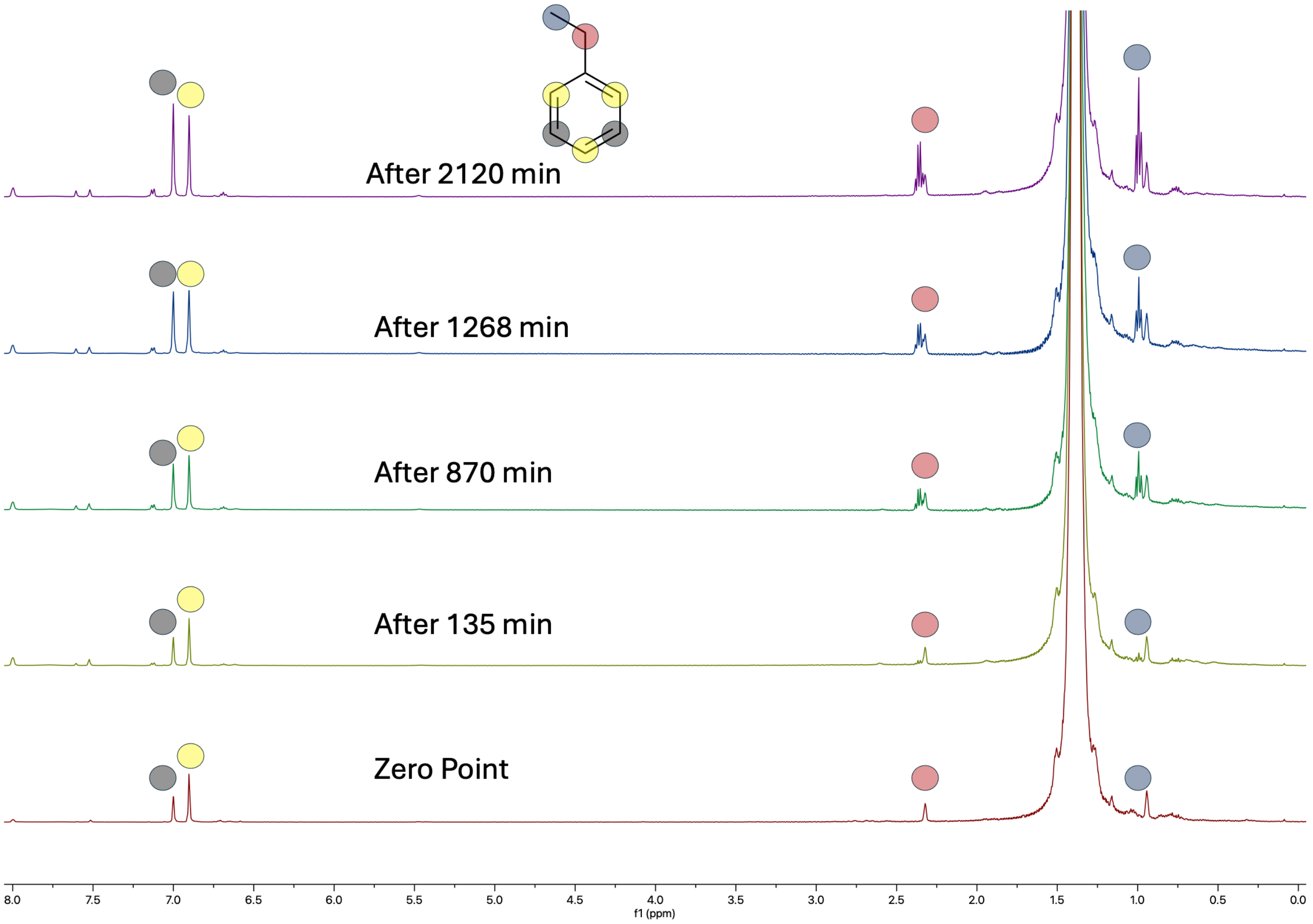

Figure S40. ^{1}H NMR spectra of H/D exchange intramolecular competition of toluene with COA by (^{iPr}PCP)IrH^{+} in ethylbenzene-d_{10} : COA-h_{16} (0.4 mL : 0.1 mL) using ortho protons as standard.

S7. Crystallographic Data

S7.a Crystallographic data for (^{iPr}PCP)IrH(CO)$_2$[BArF^{24}]

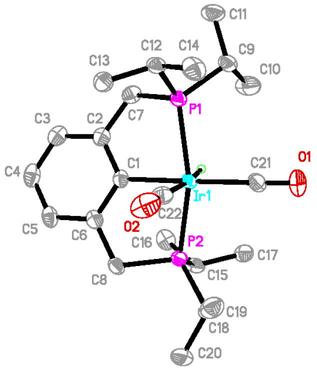

Figure S41. ORTEP representation (50% probability ellipsoids) of (^{iPr}PCP)IrH(CO)$_2$[BArF^{24}] determined by SDXRD. Hydrogen atoms other than those of hydride ligand and BArF^{24} anion omitted for clarity.

Crystal data and structure refinement for AP-632a_faces.

Identification code	AP-632a_faces	
Empirical formula	C60 H54 B F24 Ir O2 P2	
Formula weight	1527.98	
Temperature	100(2) K	
Wavelength	1.54184 Å	
Crystal system	Monoclinic	
Space group	$P2_1/n$	
Unit cell dimensions	a = 13.95055(12) Å	a= 90°.
	b = 16.06162(13) Å	b= 101.9841(8)°.
	c = 28.0208(2) Å	g = 90°.
Volume	6141.73(9) Å^3	
Z	4	
Density (calculated)	1.652 Mg/m^3	
Absorption coefficient	5.744 mm^{-1}	
F(000)	3032	
Crystal size	0.240 x 0.050 x 0.040 mm^3	
Theta range for data collection	3.189 to 68.246°.	
Index ranges	-15<=h<=16, -19<=k<=15, -33<=l<=33	
Reflections collected	60254	
Independent reflections	11219 [R(int) = 0.0419]	
Completeness to theta = 67.684°	99.7 %	
Absorption correction	Gaussian	
Max. and min. transmission	1.000 and 0.565	
Refinement method	Full-matrix least-squares on F^2	
Data / restraints / parameters	11219 / 85 / 883	
Goodness-of-fit on F^2	1.069	
Final R indices [I>2sigma(I)]	R1 = 0.0336, wR2 = 0.0823	
R indices (all data)	R1 = 0.0379, wR2 = 0.0848	
Extinction coefficient	n/a	
Largest diff. peak and hole	2.626 and -1.265 e.Å^{-3}	

S7.b Crystallographic data for (^{iPr}POCP^{tBu})IrH(CO)$_2$[BArF^{24}]

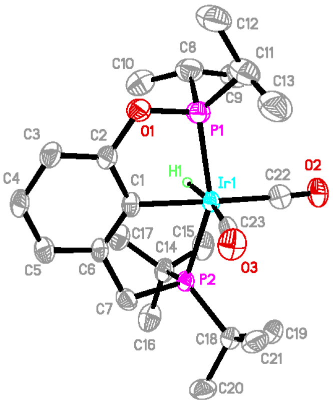

Figure S42. ORTEP representation (50% probability ellipsoids) of (^{iPr}POCP^{tBu})IrH(CO)$_2$[BArF^{24}] determined by SDXRD. Hydrogen atoms other than those of hydride ligand and BArF^{24} anion omitted for clarity.

Identification code	AP-492_faces	
Empirical formula	C55 H50 B F24 Ir O3 P2	
Formula weight	1479.90	
Temperature	100(2) K	
Wavelength	1.54184 Å	
Crystal system	Triclinic	
Space group	P-1	
Unit cell dimensions	a = 12.1549(5) Å	a= 77.198(2)°.
	b = 12.4547(4) Å	b= 83.847(3)°.
	c = 20.2953(6) Å	g = 86.917(3)°.
Volume	2977.38(18) Å^3	
Z	2	
Density (calculated)	1.651 Mg/m^3	
Absorption coefficient	5.914 mm^{-1}	
F(000)	1464	
Crystal size	0.080 x 0.080 x 0.020 mm^3	
Theta range for data collection	3.641 to 70.075°.	
Index ranges	-14<=h<=14, -15<=k<=15, -24<=l<=24	
Reflections collected	41500	
Independent reflections	11149 [R(int) = 0.0620]	
Completeness to theta = 67.684°	99.1 %	
Absorption correction	Gaussian	
Max. and min. transmission	0.996 and 0.676	
Refinement method	Full-matrix least-squares on F^2	
Data / restraints / parameters	11149 / 177 / 817	
Goodness-of-fit on F^2	1.041	
Final R indices [I>2sigma(I)]	R1 = 0.0507, wR2 = 0.1208	
R indices (all data)	R1 = 0.0582, wR2 = 0.1249	
Extinction coefficient	n/a	
Largest diff. peak and hole	2.109 and -1.678 e.Å^{-3}	

S7.c Crystallographic data for [(^{iPr}PCP)IrH(COE)(H_2O)][BArF^{24}]

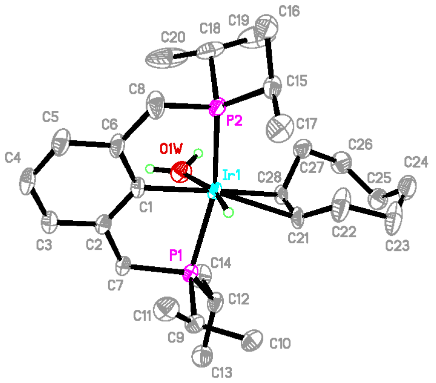

Figure S43. ORTEP representation (50% probability ellipsoids) of [(^{iPr}PCP)IrH(COE)(H_2O)][BArF^{24}] determined by SDXRD. Hydrogen atoms other than those of hydride ligand and BArF^{24} anion omitted for clarity.

Identification code	AP-522b_auto_a	
Empirical formula	C66 H70 B F24 Ir O P2	
Formula weight	1600.17	
Temperature	100(2) K	
Wavelength	1.54184 Å	
Crystal system	Triclinic	
Space group	P-1	
Unit cell dimensions	a = 12.66887(17) Å	a= 86.4135(7)°.
	b = 13.43922(13) Å	b= 84.2356(9)°.
	c = 19.83396(15) Å	g = 86.0322(9)°.
Volume	3346.53(6) Å^3	
Z	2	
Density (calculated)	1.588 Mg/m^3	
Absorption coefficient	5.285 mm^{-1}	
F(000)	1604	
Crystal size	0.250 x 0.170 x 0.050 mm^3	
Theta range for data collection	4.095 to 70.070°.	
Index ranges	-15<=h<=15, -15<=k<=16, -24<=l<=24	
Reflections collected	77359	
Independent reflections	12704 [R(int) = 0.0661]	
Completeness to theta = 67.684°	99.9 %	
Absorption correction	Semi-empirical from equivalents	
Max. and min. transmission	1.00000 and 0.61381	
Refinement method	Full-matrix least-squares on F^2	
Data / restraints / parameters	12704 / 2000 / 968	
Goodness-of-fit on F^2	1.051	
Final R indices [I>2sigma(I)]	R1 = 0.0330, wR2 = 0.0882	
R indices (all data)	R1 = 0.0338, wR2 = 0.0889	
Extinction coefficient	n/a	
Largest diff. peak and hole	1.363 and -2.084 e.Å^{-3}	

S7.d Crystallographic data for [(^{iPr}PCP)IrH(C_3H_6)(H_2O)][BArF^{24}]

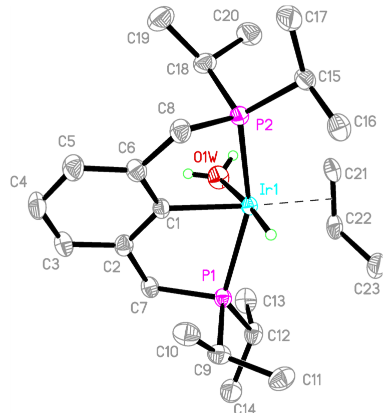

Figure S44. ORTEP representation (50% probability ellipsoids) of [(^{iPr}PCP)IrH(C_3H_6)(H_2O)][BArF^{24}] determined by SDXRD. Hydrogen atoms other than those on water molecules and hydride ligand omitted for clarity. BArF^{24} anion and benzene solvate are also omitted for clarity.

Identification code	AP-649_block_faces_a	
Empirical formula	C62 H64 B F24 Ir O P2	
Formula weight	1546.08	
Temperature	100(2) K	
Wavelength	1.54184 Å	
Crystal system	Monoclinic	
Space group	$P2_1/c$	
Unit cell dimensions	a = 19.52580(10) Å	a= 90°.
	b = 13.06660(10) Å	b= 97.53°.
	c = 24.93880(10) Å	g = 90°.
Volume	6307.85(6) $Å^3$	
Z	4	
Density (calculated)	1.628 Mg/m^3	
Absorption coefficient	5.585 mm^{-1}	
F(000)	3088	
Crystal size	0.190 x 0.120 x 0.100 mm^3	
Theta range for data collection	3.575 to 68.246°.	
Index ranges	-23<=h<=23, -15<=k<=15, -29<=l<=30	
Reflections collected	50887	
Independent reflections	11539 [R(int) = 0.0275]	
Completeness to theta = 67.684°	99.9 %	
Absorption correction	Gaussian	
Max. and min. transmission	1.000 and 0.687	
Refinement method	Full-matrix least-squares on F^2	
Data / restraints / parameters	11539 / 1831 / 930	
Goodness-of-fit on F^2	1.034	
Final R indices [I>2sigma(I)]	R1 = 0.0249, wR2 = 0.0608	
R indices (all data)	R1 = 0.0260, wR2 = 0.0616	
Extinction coefficient	n/a	
Largest diff. peak and hole	1.086 and -0.757 e.$Å^{-3}$	

S8. Computational Details

All electronic structure calculations employed the DFT method. Geometry optimization and vibrational analyses were carried out in the gas phase using the M06L density functional as implemented in *Gaussian-16*.[S5, 6] For this purpose the 6-311G(d,p) basis set was used for the main group elements,[S7] while iridium carried the SDD relativistic effective core potential and associated basis set augmented with one *f* polarization function.[S8, 9] Final electronic energies were obtained in a polarizable continuum representing toluene as solvent[S10] via single point calculations on the gas-phase geometries using the M06L, ωB97X-D[S11], B3LYP-D3BJ, PBE0-D3BJ[S12- 14] density functionals, employing this time the def2-tzvp basis set on the main group elements and the def2-qzvp basis set with associated ECP on Ir.[S15, 16] The enthalpy and Gibbs free energy terms were obtained from the gas phase calculations at 298.15 K and adjusted to 1 M.[S17, 18]Values differed among the different functionals, but similar trends were found for the entire range of alkanes investigated. The ^{iPr}PCP ligand can define several conformation; for practical considerations we limited the calculations to the conformer observed for [**1-H-COE(H_2O)**][BArF^{24}] in Figure 14.

S9. Calculated Thermodynamic Quantities

S9.a Thermodynamic quantities, Mechanism A

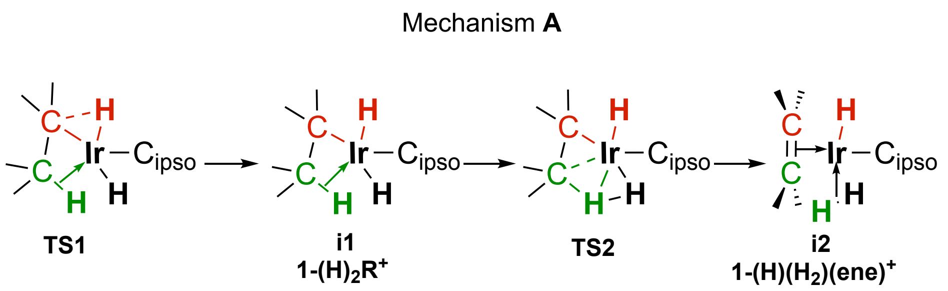

Table S26. M06L gas phase activation and reaction enthalpies for the species on mechanism A relative to the separated 1-H$^+$ and the alkane; at 298K in kcal/mol.

$\Delta H°$ or $\Delta H^‡$ M06L-gas	**TS1** **C-H addition**	**1-(H)$_2$R$^+$** **C-H addition prod.**	**TS2** **β-H-transfer**	**1-H(H$_2$)(alkene)$^+$** **β-H transfer prod.**
TBA	8.8	8.8	12.1	1.2
n-butane	8.6	7.9	9.3	-1.6
CPA	6.6	6.1	9.3	-3.3
CHxA (trans)	16.2	16.7	29.4	27.7
CHxA (cis)	13.7	12.3	13.6	3.2
CHpA	6.9	4.8	5.5	-6.1
COA	7.3	4.3	4.2	-2.8
CDA (cis)	9.2	7.9	9.7	-5.4
CDA (trans)	10.2	7.7	7.9	-4.3

Table S27. M06L gas phase activation and reaction entropies for the species on mechanism A relative to the separated 1-H$^+$ and the alkane; at 298K and adjusted to 1M; in kcal/mol.

$\Delta S°$ or $\Delta S^‡$ M06L-gas	**TS1** **C-H addition**	**1-(H)$_2$R$^+$** **C-H addition prod**	**TS2** **β-H-transfer**	**1-H(H$_2$)(alkene)$^+$** **β-H transfer prod**
TBA	-50.5	-49.5	-53.0	-50.6
n-butane	-48.8	-48.8	-52.5	-48.2
CPA	-58.8	-57.6	-58.3	-51.9
CHxA (trans)	-44.7	-42.6	-46.2	-41.8
CHxA (cis)	-45.8	-45.0	-48.5	-42.6
CHpA	-50.7	-50.9	-53.7	-48.6
COA	-51.6	-51.2	-53.3	-44.5
CDA (cis)	-52.2	-51.5	-50.6	-46.4
CDA (trans)	-51.8	-52.2	-50.4	-42.6

Table S28. M06L gas phase activation and reaction Gibbs free energies for species on mechanism A relative to the sep. 1-H$^+$ and the alkane; at 298K; adjusted to 1M; in kcal/mol.

$\Delta G°$ or $\Delta G^‡$ M06L-gas	**TS1 C-H addition**	**1-(H)$_2$R$^+$ C-H addition prod**	**TS2 β-H-transfer**	**1-H(H$_2$)(alkene)$^+$ β-H transfer prod**
TBA	23.9	23.6	27.9	16.3
n-butane	23.2	22.4	25.0	12.8
CPA	24.2	23.3	26.7	12.2
CHxA (trans)	29.5	29.4	43.2	40.2
CHxA (cis)	27.4	25.7	28.1	15.9
CHpA	22.0	20.0	21.5	8.4
COA	22.7	19.6	20.1	10.5
CDA (cis)	24.8	23.3	24.8	8.5
CDA (trans)	25.6	23.3	22.9	8.4

Table S29. M06L activation and reaction Gibbs free energies in Toluene continuum for species on mechanism A relative to sep 1-H$^+$ and alkane; at 298K; adjusted to 1M; in kcal/mol.

$\Delta G°$ or $\Delta G^‡$ M06L-sol	**TS1 C-H addition**	**1-(H)$_2$R$^+$ C-H addition prod**	**TS2 β-H-transfer**	**1-H(H$_2$)(alkene)$^+$ β-H transfer prod**
TBA	33.8	34.1	38.5	27.6
n-butane	33.3	33.1	36.4	23.5
CPA	35.1	35.0	38.8	23.2
CHxA (trans)	40.5	40.5	55.6	52.2
CHxA (cis)	38.5	37.8	40.4	27.0
CHpA	33.3	32.0	33.8	20.0
COA	34.2	32.0	32.6	21.7
CDA (cis)	36.7	35.8	37.8	20.2
CDA (trans)	37.5	35.8	35.4	20.1

Table S30. B3LYP-D3BJ activation and reaction Gibbs free energies in Toluene continuum for species on mechanism A relative to sep 1-H$^+$ and alkane; at 298K; adjusted to 1M; in kcal/mol.

$\Delta G°$ or $\Delta G^‡$ B3LYP-D3BJ-sol	**TS1 C-H addition**	**1-(H)$_2$R$^+$ C-H addition prod**	**TS2 β-H-transfer**	**1-H(H$_2$)(alkene)$^+$ β-H transfer prod**
TBA	28.0	27.8	31.2	20.8
n-butane	27.6	26.8	29.9	17.2
CPA	30.6	29.9	32.7	17.9
CHxA (trans)	35.6	35.5	49.5	46.0
CHxA (cis)	32.9	31.8	34.0	20.3
CHpA	26.8	25.2	26.8	12.9
COA	27.4	24.9	25.2	14.0
CDA (cis)	31.4	29.5	30.0	12.7
CDA (trans)	31.7	29.4	28.0	12.3

Table S31. ωB97X-D activation and reaction Gibbs free energies in Toluene continuum for species on mechanism A relative to sep 1-H$^+$ and alkane; at 298K; adjusted to 1M; in kcal/mol.

$\Delta G°$ or $\Delta G^‡$ ωB97X-D-sol	**TS1 C-H addition**	**1-(H)$_2$R$^+$ C-H addition prod**	**TS2 β-H-transfer**	**1-H(H$_2$)(alkene)$^+$ β-H transfer prod**
TBA	25.8	25.8	28.6	19.8
n-butane	25.5	24.9	27.1	16.1
CPA	28.6	28.3	30.8	17.8
CHxA (trans)	34.9	34.9	47.9	45.5
CHxA (cis)	31.2	30.1	31.7	19.9
CHpA	24.9	23.3	24.1	12.2
COA	25.4	22.9	22.5	14.4
CDA (cis)	28.8	27.1	27.4	12.6
CDA (trans)	28.6	26.7	24.3	12.5

Table S32. PBE0-D3BJ activation and reaction Gibbs free energies in Toluene continuum for species on mechanism A relative to sep 1-H$^+$ and alkane; at 298K; adjusted to 1M; in kcal/mol.

$\Delta G°$ or $\Delta G^‡$ PBE0-D3BJ-sol	**TS1 C-H addition**	**1-(H)$_2$R$^+$ C-H addition prod**	**TS2 β-H-transfer**	**1-H(H$_2$)(alkene)$^+$ β-H transfer prod**
TBA	23.0	23.1	25.2	18.9
n-butane	22.0	21.5	23.1	14.3
CPA	25.3	25.0	26.7	16.7
CHxA (trans)	31.3	31.4	43.0	41.8
CHxA (cis)	27.6	26.7	27.6	18.3
CHpA	21.5	20.2	20.6	11.0
COA	21.9	21.4	19.1	13.6
CDA (cis)	26.2	24.3	24.3	12.3
CDA (trans)	26.6	24.9	22.4	12.0

S9.b Thermodynamic quantities, Mechanism B

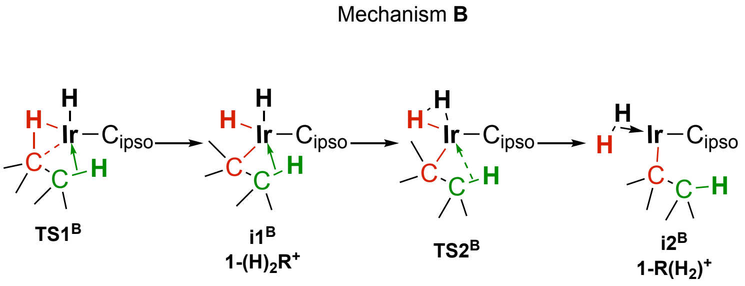

Table S33. M06L gas phase activation and reaction enthalpies for the species on mechanism B relative to the separated 1-H$^+$ and the alkane; at 298K in kcal/mol.

$\Delta H°$ or $\Delta H^‡$ M06L-gas	**TS1^B C-H addition**	**i1^B cis-1-(H)$_2$R$^+$**	**TS2^B (H-H-bond formation)**	**i2^B 1-(alkyl)(H$_2$)$^+$**
n-butane	10.1	10.6	20.1	5.7
CPA	11.2	11.6	19.5	6.0
COA	5.6	6.2	15.3	6.0
benzene	12.5	13.1	16.1	-0.9
toluene	8.8	7.9	15.5	1.9

Table S34. M06L gas phase activation and reaction entropies for the species on mechanism B relative to the separated 1-H$^+$ and the alkane; at 298K and adjusted to 1M; in kcal/mol.

$\Delta S°$ or $\Delta S^‡$ M06L-gas	**TS1^B C-H addition**	**i1^B cis-1-(H)$_2$R$^+$**	**TS2^B (H-H-bond formation)**	**i2^B 1-(alkyl)(H$_2$)$^+$**
n-butane	-49.2	-47.9	-43.6	-41.2
CPA	-56.3	-55.5	-51.0	-48.7
COA	-51.8	-50.2	-48.9	-45.8
benzene	-39.4	-36.4	-36.9	-37.7
toluene	-53.3	-53.1	-52.4	-48.5

Table S35. M06L gas phase activation and reaction Gibbs free energies for species on mechanism B relative to the sep. 1-H$^+$ and the alkane; at 298K; adjusted to 1M; in kcal/mol.

$\Delta G°$ or $\Delta G^‡$ M06L-gas	**TS1 C-H addition**	**1-(H)$_2$R$^+$ C-H addition prod**	**TS2 β-H-transfer**	**i2^B 1-(alkyl)(H$_2$)$^+$**
n-butane	24.8	24.9	**33.1**	18.0
CPA	28.0	28.2	34.7	20.5
COA	21.0	21.2	**29.9**	19.6
benzene	24.3	23.9	27.1	10.3
toluene	24.7	23.8	31.2	16.3

Table S36. M06L activation and reaction Gibbs free energies in Toluene continuum for species on mechanism B relative to sep 1-H^+ and alkane; at 298K; adjusted to 1M; in kcal/mol.

$\Delta G°$ or $\Delta G^‡$ M06L-sol	**$TS1^B$ C-H addition**	**$i1^B$ cis-1-$(H)_2R^+$**	**$TS2^B$ (H-H-bond formation)**	**$i2^B$ 1-(alkyl)$(H_2)^+$**
n-butane	35.8	36.2	42.4	25.4
CPA	39.3	39.8	44.8	29.2
COA	32.8	33.3	40.8	29.3
benzene	34.9	35.0	37.8	18.8
toluene	38.5	37.6	42.8	26.3

Table S37. B3LYP-D3BJ activation and reaction Gibbs free energies in Toluene continuum for species on mechanism A relative to sep 1-H^+ and alkane; at 298K; adjusted to 1M; in kcal/mol.

$\Delta G°$ or $\Delta G^‡$ B3LYP-D3BJ-sol	**$TS1^B$ C-H addition**	**$i1^B$ cis-1-$(H)_2R^+$**	**$TS2^B$ (H-H-bond formation)**	**$i2^B$ 1-(alkyl)$(H_2)^+$**
n-butane	29.5	29.7	36.4	21.3
CPA	33.0	33.2	39.0	24.7
COA	26.0	26.3	35.4	25.1
benzene	28.5	28.5	31.4	13.4
toluene	31.4	30.3	36.8	20.9

Table S38. ωB97X-D activation and reaction Gibbs free energies in Toluene continuum for species on mechanism B relative to sep 1-H^+ and alkane; at 298K; adjusted to 1M; in kcal/mol

$\Delta G°$ or $\Delta G^‡$ ωB97X-D-sol	**$TS1^B$ C-H addition**	**$i1^B$ cis-1-$(H)_2R^+$**	**$TS2^B$ (H-H-bond formation)**	**$i2^B$ 1-(alkyl)$(H_2)^+$**
n-butane	28.0	28.4	37.0	21.5
CPA	31.7	32.2	38.7	25.2
COA	24.3	24.9	35.3	25.8
benzene	29.7	29.9	32.8	14.9
toluene	32.7	31.7	39.0	23.0

Table S39. PBE0-D3BJ activation and reaction Gibbs free energies in Toluene continuum for species on mechanism B relative to sep 1-H^+ and alkane; at 298K; adjusted to 1M; in kcal/mol

$\Delta G°$ or $\Delta G^‡$ PBE0-D3BJ-sol	**$TS1^B$ C-H addition**	**$i1^B$ cis-1-$(H)_2R^+$**	**$TS2^B$ (H-H-bond formation)**	**$i2^B$ 1-(alkyl)$(H_2)^+$**
n-butane	24.8	22.0	29.5	17.8
CPA	28.7	29.1	36.8	23.6
COA	21.8	22.3	33.1	24.1
benzene	25.2	25.4	28.7	11.7
toluene	27.9	26.9	35.3	20.8

S.10 References

S1. Parihar, A.; Emge, T. J.; Chakravartula, S. V. S.; Goldman, A. S., Pincer-Ligated Iridium Complexes with Low-Field Ancillary Ligands: Complexes of (iPrPCP)IrCl2 and Comparison with (iPrPCP)IrHCl. *Organometallics* **2024,** *43* (11), 1317-1327.

S2. Punji, B.; Emge, T. J.; Goldman, A. S., A Highly Stable Adamantyl-Substituted Pincer-Ligated Iridium Catalyst for Alkane Dehydrogenation. *Organometallics* **2010,** *29* (12), 2702-2709.

S3. Nawara-Hultzsch, A. J.; Hackenberg, J. D.; Punji, B.; Supplee, C.; Emge, T. J.; Bailey, B. C.; Schrock, R. R.; Brookhart, M.; Goldman, A. S., Rational Design of Highly Active "Hybrid" Phosphine-Phosphinite Pincer Iridium Catalysts for Alkane Metathesis. *ACS Catal.* **2013,** *3* (11), 2505-2514.

S4. Moulton, C. J.; Shaw, B. L., Transition Metal-Carbon Bonds. Part XLII. Complexes of Nickel, Palladium, Platinum, Rhodium and Iridium with the Tridentate Ligand 2,6-bis[(di-tert-butylphosphino)methyl]phenyl. *J. Chem. Soc., Dalton Trans.* **1976,** (11), 1020-1024.

S5. Zhao, Y.; Truhlar, D. G., A new local density functional for main-group thermochemistry, transition metal bonding, thermochemical kinetics, and noncovalent interactions. *J. Chem. Phys.* **2006,** *125* (19), 194101/1-194101/18.

S6. Frisch, M. J.; Trucks, G. W.; Schlegel, H. B.; Scuseria, G. E.; Robb, M. A.; Cheeseman, J. R.; Scalmani, G.; Barone, V.; Petersson, G. A.; Nakatsuji, H.; Li, X.; Caricato, M.; Marenich, A. V.; Bloino, J.; Janesko, B. G.; Gomperts, R.; Mennucci, B.; Hratchian, H. P.; Ortiz, J. V.; Izmaylov, A. F.; Sonnenberg, J. L.; Williams-Young, D.; Ding, F.; Lipparini, F.; Egidi, F.; Goings, J.; Peng, B.; Petrone, A.; Henderson, T.; Ranasinghe, D.; Zakrzewski, V. G.; Gao, J.; Rega, N.; Zheng, G.; Liang, W.; Hada, M.; Ehara, M.; Toyota, K.; Fukuda, R.; Hasegawa, J.; Ishida, M.; Nakajima, T.; Honda, Y.; Kitao, O.; Nakai, H.; Vreven, T.; Throssell, K.; Montgomery, J. A., Jr.; Peralta, J. E.; Ogliaro, F.; Bearpark, M. J.; Heyd, J. J.; Brothers, E. N.; Kudin, K. N.; Staroverov, V. N.; Keith, T. A.; Kobayashi, R.; Normand, J.; Raghavachari, K.; Rendell, A. P.; Burant, J. C.; Iyengar, S. S.; Tomasi, J.; Cossi, M.; Millam, J. M.; Klene, M.; Adamo, C.; Cammi, R.; Ochterski, J. W.; Martin, R. L.; Morokuma, K.; Farkas, O.; Foresman, J. B.; Fox, D. J. *Gaussian 16, Revision D.01*, Gaussian, Inc.: Wallingford CT,, 2016.

S7. Krishnan, R.; Binkley, J. S.; Seeger, R.; Pople, J. A., Self-consistent molecular orbital methods. XX. A basis set for correlated wave functions. *J. Chem. Phys.* **1980,** *72* (1), 650-654.

S8. Andrae, D.; Haeussermann, U.; Dolg, M.; Stoll, H.; Preuss, H., Energy-adjusted ab initio pseudopotentials for the second and third row transition elements. *Theor. Chim. Acta* **1990,** *77* (2), 123-141.

S9. Ehlers, A. W.; Böhme, M.; Dapprich, S.; Gobbi, A.; Höllwarth, A.; Jonas, V.; Köhler, K. F.; Stegmann, R.; Veldkamp, A.; Frenking, G., A set of f-polarization functions for pseudo-potential basis sets of the transition metals Sc–Cu, Y–Ag and La–Au. *Chem. Phys. Lett.* **1993,** *208* (1), 111-114.

S10. Marenich, A. V.; Cramer, C. J.; Truhlar, D. G., Universal Solvation Model Based on Solute Electron Density and on a Continuum Model of the Solvent Defined by the Bulk Dielectric Constant and Atomic Surface Tensions. *J. Phys. Chem. B* **2009,** *113* (18), 6378-6396.

S11. Chai, J.-D.; Head-Gordon, M., Long-range corrected hybrid density functionals with damped atom–atom dispersion corrections. *Phys. Chem. Chem. Phys.* **2008,** *10* (44), 6615-6620.

S12. Lee, C.; Yang, W.; Parr, R. G., Development of the Colle-Salvetti correlation-energy formula into a functional of the electron density. *Phys. Rev. B* **1988,** *37* (2), 785-789.

S13. Adamo, C.; Barone, V., Toward reliable density functional methods without adjustable parameters: The PBE0 model. *J. Chem. Phys.* **1999,** *110* (13), 6158-6170.

S14. Grimme, S.; Antony, J.; Ehrlich, S.; Krieg, H., A consistent and accurate ab initio parametrization of density functional dispersion correction (DFT-D) for the 94 elements H-Pu. *J. Chem. Phys.* **2010,** *132* (15), 154104.

S15. Weigend, F.; Ahlrichs, R., Balanced basis sets of split valence, triple zeta valence and quadruple zeta valence quality for H to Rn: Design and assessment of accuracy. *Phys. Chem. Chem. Phys.* **2005,** *7* (18), 3297-3305.

S16. Weigend, F., Accurate Coulomb-fitting basis sets for H to Rn. *Phys. Chem. Chem. Phys.* **2006,** *8* (9), 1057-1065.

S17. Ochterski-Thermochemistry-Gaussian, Ochterski, J. W. Thermochemistry in Gaussian; page 13.

S18. Cramer, C. J., *Essentials of Computational Chemistry: Theories and Models, 2nd Edition*. Wiley: 2004.
